# Author Correction: Mapping local patterns of childhood overweight and wasting in low- and middle-income countries between 2000 and 2017

**DOI:** 10.1038/s41591-020-0972-7

**Published:** 2020-07-02

**Authors:** LBD Double Burden of Malnutrition Collaborators, Damaris K. Kinyoki, Jennifer M. Ross, Alice Lazzar-Atwood, Sandra B. Munro, Lauren E. Schaeffer, Mahdieh Abbasalizad-Farhangi, Masoumeh Abbasi, Hedayat Abbastabar, Ahmed Abdelalim, Amir Abdoli, Mohammad Abdollahi, Ibrahim Abdollahpour, Rizwan Suliankatchi Abdulkader, Nebiyu Dereje Abebe, Teshome Abuka Abebo, Kedir Hussein Abegaz, Hassan Abolhassani, Lucas Guimarães Abreu, Michael R. M. Abrigo, Abdelrahman I. Abushouk, Manfred Mario Kokou Accrombessi, Dilaram Acharya, Maryam Adabi, Akindele Olupelumi Adebiyi, Isaac Akinkunmi Adedeji, Victor Adekanmbi, Abiodun Moshood Adeoye, Olatunji O. Adetokunboh, Davoud Adham, Posi Emmanuel Aduroja, Shailesh M. Advani, Mohsen Afarideh, Mohammad Aghaali, Anurag Agrawal, Tauseef Ahmad, Keivan Ahmadi, Sepideh Ahmadi, Muktar Beshir Ahmed, Rushdia Ahmed, Olufemi Ajumobi, Chalachew Genet Akal, Temesgen Yihunie Akalu, Tomi Akinyemiju, Blessing Akombi, Ziyad Al-Aly, Samiah Alam, Genet Melak Alamene, Turki M. Alanzi, Jacqueline Elizabeth Alcalde Rabanal, Niguse Meles Alema, Beriwan Abdulqadir Ali, Muhammad Ali, Mehran Alijanzadeh, Cyrus Alinia, Vahid Alipour, Hesam Alizade, Syed Mohamed Aljunid, Afshin Almasi, Amir Almasi-Hashiani, Hesham M. Al-Mekhlafi, Rajaa M. Al-Raddadi, Khalid Altirkawi, Nelson Alvis-Guzman, Nelson J. Alvis-Zakzuk, Azmeraw T. Amare, Adeladza Kofi Amegah, Saeed Amini, Mostafa Amini Rarani, Fatemeh Amiri, Arianna Maever Loreche Amit, Nahla Hamed Anber, Catalina Liliana Andrei, Fereshteh Ansari, Alireza Ansari-Moghaddam, Zelalem Alamrew Anteneh, Carl Abelardo T. Antonio, Ernoiz Antriyandarti, Davood Anvari, Razique Anwer, Seth Christopher Yaw Appiah, Jalal Arabloo, Morteza Arab-Zozani, Ephrem Mebrahtu Araya, Zohreh Arefi, Olatunde Aremu, Johan Ärnlöv, Afsaneh Arzani, Mehran Asadi-Aliabadi, Ali A. Asadi-Pooya, Samaneh Asgari, Babak Asghari, Alebachew Fasil Ashagre, Anemaw A. Asrat, Bahar Ataeinia, Hagos Tasew Atalay, Desta Debalkie Atnafu, Maha Moh’d Wahbi Atout, Marcel Ausloos, Euripide F. G. A. Avokpaho, Ashish Awasthi, Beatriz Paulina Ayala Quintanilla, Martin Amogre Ayanore, Yared A. Asmare Aynalem, Abbas Azadmehr, Samad Azari, Ghasem Azarian, Zelalem Nigussie Azene, Ebrahim Babaee, Alaa Badawi, Ashish D. Badiye, Mohamad Amin Bahrami, Atif Amin A. Baig, Ahad Bakhtiari, Shankar M. Bakkannavar, Senthilkumar Balakrishnan, Ayele Geleto Bali, Maciej Banach, Palash Chandra Banik, Zahra Baradaran-Seyed, Adhanom Gebreegziabher Baraki, Miguel A. Barboza, Till Winfried Bärnighausen, Lingkan Barua, Huda Basaleem, Sanjay Basu, Mohsen Bayati, Mulat Tirfie Bayih, Habtamu Wondifraw Baynes, Neeraj Bedi, Masoud Behzadifar, Meysam Behzadifar, Yibeltal Alemu Bekele, Derrick A. Bennett, Dessalegn Ajema Berbada, Kidanemaryam Berhe, Abadi Kidanemariam Berhe, Adam E. Berman, Robert S. Bernstein, Reshmi Bhageerathy, Dinesh Bhandari, Pankaj Bharadwaj, Natalia V. Bhattacharjee, Krittika Bhattacharyya, Ali Bijani, Boris Bikbov, Ver Bilano, Nigus Bililign, Muhammad Shahdaat Bin Sayeed, Setognal Birara, Minuye Biniam Biniam Birhane, Minyichil Birhanu, Raaj Kishore Biswas, Zebenay Workneh Bitew, Kassawmar Angaw Bogale, Somayeh Bohlouli, Srinivasa Rao Bolla, Archith Boloor, Antonio M. Borzì, Shiva Borzouei, Oliver J. Brady, Nicola Luigi Bragazzi, Dejana Braithwaite, Nikolay Ivanovich Briko, Gabrielle Britton, Shyam S. Budhathoki, Sharath Burugina Nagaraja, Reinhard Busse, Zahid A. Butt, Lucero Cahuana-Hurtado, Luis Alberto Cámera, Ismael R. Campos-Nonato, Jorge Cano, Josip Car, Rosario Cárdenas, Juan J. Carrero, Félix Carvalho, João Mauricio Castaldelli-Maia, Carlos A. Castañeda-Orjuela, Franz Castro, Ester Cerin, Collins Chansa, Jaykaran Charan, Pranab Chatterjee, Vijay Kumar Chattu, Bal Govind Chauhan, Ali Reza Chavshin, Mohammad Chehrazi, Tesfaye Yitna Chichiabellu, Ken Lee Chin, Devasahayam J. Christopher, Dinh-Toi Chu, Flavia M. Cicuttini, Michael L. Collison, Michael A. Cork, Natalie Cormier, Paolo Angelo Cortesi, Vera M. Costa, Abel Fekadu Fekadu Dadi, Baye Dagnew, Saad M. A. Dahlawi, Giovanni Damiani, Amira Hamed Darwish, Ahmad Daryani, Jai K. Das, Rajat Das Gupta, Claudio Dávila-Cervantes, Nicole Davis Weaver, Diego De Leo, Jan-Walter De Neve, Feleke Mekonnen Demeke, Asmamaw Bizuneh Demis, Dereje Bayissa Demissie, Gebre Teklemariam Demoz, Edgar Denova-Gutiérrez, Kebede Deribe, Rupak Desai, Beruk Berhanu Desalegn, Assefa Desalew, Aniruddha Deshpande, Sagnik Dey, Samath Dhamminda Dharmaratne, Preeti Dhillon, Meghnath Dhimal, Govinda Prasad Dhungana, Mostafa Dianati Nasab, Daniel Diaz, Zahra Sadat Dibaji Forooshani, Girmaye Deye Dinsa, Isaac Oluwafemi Dipeolu, Shirin Djalalinia, Hoa Thi Do, Huyen Phuc Do, Paul Narh Doku, Fariba Dorostkar, Leila Doshmangir, Manisha Dubey, Bereket Duko Adema, Susanna J. Dunachie, Bruce B. Duncan, Ewerton Cousin, Andre R. Durães, Lucas Earl, Hamed Ebrahimzadeh Leylabadlo, Aziz Eftekhari, Iman El Sayed, Maysaa El Sayed Zaki, Maha El Tantawi, Iffat Elbarazi, Demelash Abewa Elemineh, Shaimaa I. El-Jaafary, Ziad El-Khatib, Aisha Elsharkawy, Yasser Mohamed El-Sherbiny, Iqbal R. F. Elyazar, Mohammad Hassan Emamian, Shymaa Enany, Daniel Adane Endalew, Melese Linger Endalifer, Khalil Eskandari, Sharareh Eskandarieh, Saman Esmaeilnejad, Alireza Esteghamati, Arash Etemadi, Atkilt Esaiyas Etisso, Jessica Fanzo, Mohammad Farahmand, Anwar Faraj, Sajjad Farashi, Mohammad Fareed, Andrea Farioli, Andre Faro, Farshad Farzadfar, Hossein Farzam, Syeda Sadia Fatima, Nazir Fattahi, Nelsensius Klau Fauk, Ali Akbar Fazaeli, Netsanet Fentahun, Tomas Y. Ferede, Seyed-Mohammad Fereshtehnejad, Eduarda Fernandes, João C. Fernandes, Garumma Tolu Feyissa, Irina Filip, Florian Fischer, Carsten Flohr, Nataliya A. Foigt, Morenike Oluwatoyin Folayan, Artem Alekseevich Fomenkov, Masoud Foroutan, Jana Förster, Joel Msafiri Francis, Takeshi Fukumoto, Reta Tsegaye Gayesa, Biniyam Sahiledengle Geberemariyam, Tsegaye Tewelde Gebrehiwot, Hadush Gebremariam, Kidane Tadesse Gebremariam, Ketema Bizuwork Bizuwork Gebremedhin, Gebreamlak Gebremedhn Gebremeskel, Assefa Ayalew Ayalew Gebreslassie, Gebretsadkan G. G. Gebretsadik, Getnet Azeze Gedefaw, Yilma Chisha Dea Geramo, Hailay Abrha Gesesew, Birhanu Geta, Agegnehu Bante Getenet, Kebede Embaye Gezae, Fatemeh Ghaffarifar, Mansour Ghafourifard, Alireza Ghajar, Mahsa Ghajarzadeh, Ahmad Ghashghaee, Hesam Ghiasvand, Asadollah Gholamian, Syed Amir Gilani, Tiffany K. Gill, Ibrahim Abdelmageed Ginawi, Srinivas Goli, Nelson G. M. Gomes, Sameer Vali Gopalani, Houman Goudarzi, Alessandra C. Goulart, Arunkumar Govindakarnavar, Ayman Grada, Michal Grivna, Rafael Alves Guimarães, Rashid Abdi Guled, Yuming Guo, Rahul Gupta, Rajeev Gupta, Nima Hafezi-Nejad, Michael Tamene Haile, Arvin Haj-Mirzaian, Arya Haj-Mirzaian, Brian J. Hall, Iman Halvaei, Randah R. Hamadeh, Yadollah Hamidi, Demelash Woldeyohannes Handiso, Graeme J. Hankey, Hamidreza Haririan, Ninuk Hariyani, Ahmed I. Hasaballah, Md. Mehedi Hasan, Milad Hasankhani, Edris Hasanpoor, Amir Hasanzadeh, Maryam Hashemian, Soheil Hassanipour, Hamid Yimam Hassen, Rasmus Havmoeller, Corinna Hawkes, Khezar Hayat, Desta Haftu Hayelom, Behnam Heidari, Reza Heidari-Soureshjani, Delia Hendrie, Andualem Henok, Nathaniel J. Henry, Mario Herrero, Claudiu Herteliu, Fatemeh Heydarpour, Hagos D. de Hidru, Chi Linh Hoang, Hans W. Hoek, Michael K. Hole, Ramesh Holla, Gillian Hollerich, Enayatollah Homaie Rad, Sung Hwi Hong, Praveen Hoogar, Masako Horino, Naznin Hossain, Mostafa Hosseini, Mehdi Hosseinzadeh, Mihaela Hostiuc, Sorin Hostiuc, Mowafa Househ, Mohamed Hsairi, Guoqing Hu, Tanvir M. Huda, Ayesha Humayun, Bing-Fang Hwang, Segun Emmanuel Ibitoye, Olayinka Stephen Ilesanmi, Milena D. Ilic, Mohammad Hasan Imani-Nasab, Leeberk Raja Inbaraj, Usman Iqbal, Seyed Sina Naghibi Irvani, Sheikh Mohammed Shariful Islam, Chidozie C. D. Iwu, Chinwe Juliana Iwu, Neda Izadi, Jalil Jaafari, Anelisa Jaca, Farhad Jadidi-Niaragh, Nader Jafari Balalami, Morteza Jafarinia, Mohammad Ali Jahani, Mihajlo Jakovljevic, Amir Jalali, Farzad Jalilian, Achala Upendra Jayatilleke, Panniyammakal Jeemon, Fyezah Jehan, Ensiyeh Jenabi, Ravi Prakash Jha, Vivekanand Jha, John S. Ji, Peng Jia, Oommen John, Yetunde O. John-Akinola, Kimberly B. Johnson, Jost B. Jonas, Nitin Joseph, Farahnaz Joukar, Jacek Jerzy Jozwiak, Suresh Banayya Jungari, Mikk Jürisson, Ali Kabir, Zubair Kabir, Amaha Kahsay, Molla Kahssay, Hamed Kalani, Leila L. Kalankesh, Rohollah Kalhor, Zahra Kamiab, Tanuj Kanchan, Umesh Kapil, Neeti Kapoor, Manoochehr Karami, Behzad Karami Matin, André Karch, Mohd A. Karim, Surendra Karki, Amir Kasaeian, Gebremicheal Gebreslassie Kasahun, Habtamu Kebebe Kasaye, Tesfaye Dessale Kassa, Hagazi Gebremedhin Kassaye, Nicholas J. Kassebaum, Ali Kazemi Karyani, Andre Pascal Kengne, Daniel Bekele Ketema, Yousef Saleh Khader, Morteza Abdullatif Khafaie, Mojtaba Khaksarian, Nauman Khalid, Ibrahim A. Khalil, Rovshan Khalilov, Asad Khan, Ejaz Ahmad Khan, Md Nuruzzaman Khan, Mohammad Saud Khan, Muhammad Shahzeb Khan, Khaled Khatab, Amir Khater, Mona M. Khater, Mahalqua Nazli Khatib, Maryam Khayamzadeh, Maryam Khazaei-Pool, Mohammad Khazaei, Salman Khazaei, Mohammad Taghi Khodayari, Mohammad Hossein Khosravi, Roba Khundkar, Ali Kiadaliri, Neda Kianipour, Daniel N. Kiirithio, Yun Jin Kim, Ruth W. Kimokoti, Adnan Kisa, Sezer Kisa, Tufa Kolola, Hamidreza Komaki, Shivakumar K. M. Kondlahalli, Ali Koolivand, Parvaiz A. Koul, Ai Koyanagi, Moritz U. G. Kraemer, Kewal Krishan, Kris J. Krohn, Nuworza Kugbey, Manasi Kumar, Pushpendra Kumar, Vivek Kumar, Om P. Kurmi, Oluwatosin Kuti, Carlo La Vecchia, Ben Lacey, Deepesh P. Lad, Aparna Lal, Dharmesh Kumar Lal, Faris Hasan Lami, Prabhat Lamichhane, Justin J. Lang, Van C. Lansingh, Savita Lasrado, Georgy Lebedev, Paul H. Lee, Shaun Wen Huey Lee, Mostafa Leili, Ian D. Letourneau, Sonia Lewycka, Shanshan Li, Lee-Ling Lim, Shai Linn, Shiwei Liu, Simin Liu, Rakesh Lodha, Joshua Longbottom, Jaifred Christian F. Lopez, Stefan Lorkowski, Erlyn Rachelle King Macarayan, Mohammed Madadin, Hassan Magdy Abd El Razek, Muhammed Magdy Abd El Razek, Dhaval P. Maghavani, Phetole Walter Mahasha, Narayan Bahadur Mahotra, Venkatesh Maled, Afshin Maleki, Shokofeh Maleki, Deborah Carvalho Malta, Ali Manafi, Farzad Manafi, Navid Manafi, Narendar Dawanu Manohar, Fariborz Mansour-Ghanaei, Borhan Mansouri, Mohammad Ali Mansournia, Chabila Christopher Mapoma, Dadi Marami, Laurie B. Marczak, Carlos Alberto Marrugo Arnedo, Francisco Rogerlândio Martins-Melo, Anthony Masaka, Benjamin Ballard Massenburg, Pallab K. Maulik, Benjamin K. Mayala, Mohsen Mazidi, Man Mohan Mehndiratta, Freshteh Mehri, Kala M. Mehta, Wahengbam Bigyananda Meitei, Fantahun Ayenew Mekonnen, Teferi Mekonnen, Gebrekiros Gebremichael Meles, Hagazi Gebre Meles, Addisu Melese, Walter Mendoza, Ritesh G. Menezes, Meresa Berwo Mengesha, George A. Mensah, Tuomo J. Meretoja, Tomasz Miazgowski, Neda Milevska Kostova, Ted R. Miller, Edward J. Mills, G. K. Mini, Seyed Mostafa Mir, Mohammad Miri, Hamed Mirjalali, Erkin M. Mirrakhimov, Hamed Mirzaei, Maryam Mirzaei, Roya Mirzaei, Mehdi Mirzaei-Alavijeh, Prasanna Mithra, Babak Moazen, Efat Mohamadi, Amjad Mohamadi-Bolbanabad, Karzan Abdulmuhsin Mohammad, Yousef Mohammad, Dara K. Mohammad, Aso Mohammad Darwesh, Naser Mohammad Gholi Mezerji, Abdollah Mohammadian-Hafshejani, Mousa Mohammadnia-Afrouzi, Milad Mohammadoo-Khorasani, Reza Mohammadpourhodki, Salahuddin Mohammed, Shafiu Mohammed, Jemal Abdu Mohammed, Ammas Siraj Mohammed, Farnam Mohebi, Amin Mokari, Ali H. Mokdad, Julio Cesar Montañez, Pablo A. Montero-Zamora, Yoshan Moodley, Maryam Moossavi, Ghobad Moradi, Masoud Moradi, Yousef Moradi, Mohammad Moradi-Joo, Maziar Moradi-Lakeh, Farhad Moradpour, Rahmatollah Moradzadeh, Paula Moraga, Shane Douglas Morrison, Abbas Mosapour, Jonathan F. Mosser, Simin Mouodi, Amin Mousavi Khaneghah, Dariush Mozaffarian, Ulrich Otto Mueller, Christopher J. L. Murray, G. V. S. Murthy, Kamarul Imran Musa, Ghulam Mustafa, Saravanan Muthupandian, Behnam Nabavizadeh, Mehdi Naderi, Girish N. Nadkarni, Ahamarshan Jayaraman Nagarajan, Mohsen Naghavi, Aliya Naheed, Gurudatta Naik, Farid Najafi, Jobert Richie Nansseu, K. M. Venkat Narayan, Bruno Ramos Nascimento, Vinod Nayak, Javad Nazari, Duduzile Edith Ndwandwe, Ionut Negoi, Ruxandra Irina Negoi, Josephine W. Ngunjiri, Cuong Tat Nguyen, Huong Lan Thi Nguyen, Dabere Nigatu, Yeshambel T. Nigatu, Rajan Nikbakhsh, Dina Nur Anggraini Ningrum, Chukwudi A. Nnaji, Vuong Minh Nong, Jean Jacques Noubiap, Christoph Nowak, Bogdan Oancea, Richard Ofori-Asenso, Onome Bright Oghenetega, In-Hwan Oh, Olanrewaju Oladimeji, Morteza Oladnabi, Andrew T. Olagunju, Tinuke O. Olagunju, Bolajoko Olubukunola Olusanya, Jacob Olusegun Olusanya, Mojisola Morenike Oluwasanu, Muktar Omer Omer, Obinna E. Onwujekwe, Kwaku Oppong Asante, Eyal Oren, Orish Ebere Orisakwe, Alberto Ortiz, Osayomwanbo Osarenotor, Aaron E. Osgood-Zimmerman, Mayowa Ojo Owolabi, Mahesh P. A., Jagadish Rao Padubidri, Keyvan Pakshir, Adrian Pana, Songhomitra Panda-Jonas, Hadi Parsian, Tahereh Pashaei, Deepak Kumar Pasupula, Sangram Kishor Patel, Ashish Pathak, Mona Pathak, Sanghamitra Pati, Ajay Patle, George C. Patton, Kebreab Paulos, Hamidreza Pazoki Toroudi, Veincent Christian Filipino Pepito, Norberto Perico, William A. Petri, Brandon V. Pickering, David M. Pigott, Majid Pirestani, Bakhtiar Piroozi, Meghdad Pirsaheb, Khem Narayan Pokhrel, Maarten J. Postma, Hadi Pourjafar, Farshad Pourmalek, Reza Pourmirza Kalhori, Akram Pourshams, Hossein Poustchi, Sergio I. Prada, Liliana Preotescu, Dimas Ria Angga Pribadi, Zahiruddin Quazi Syed, Mohammad Rabiee, Navid Rabiee, Amir Radfar, Alireza Rafiei, Fakher Rahim, Vafa Rahimi-Movaghar, Muhammad Aziz Rahman, Sajjad ur Rahman, Rajesh Kumar Rai, Ali Rajabpour-Sanati, Fatemeh Rajati, Kiana Ramezanzadeh, Saleem Muhammad Rana, Chhabi Lal Ranabhat, Sowmya J. Rao, Davide Rasella, Vahid Rashedi, Prateek Rastogi, Priya Rathi, Salman Rawaf, David Laith Rawaf, Lal Rawal, Sarah E. Ray, Giuseppe Remuzzi, Vishnu Renjith, Andre M. N. Renzaho, Serge Resnikoff, Nima Rezaei, Shahab Rezaeian, Mohammad Sadegh Rezai, Aziz Rezapour, Seyed Mohammad Riahi, Ana Isabel Ribeiro, Jennifer Rickard, Alina Rodriguez, Leonardo Roever, Elias Merdassa Roro, Gholamreza Roshandel, Ali Rostami, Enrico Rubagotti, Anas M. Saad, Seyedmohammad Saadatagah, Yogesh Damodar Sabde, Siamak Sabour, Ehsan Sadeghi, Masoumeh Sadeghi, Saeed Safari, Yahya Safari, Hamid Safarpour, Rajesh Sagar, Amirhossein Sahebkar, Mohammad Ali Sahraian, S. Mohammad Sajadi, Mohammad Reza Salahshoor, Nasir Salam, Farkhonde Salehi, Saleh Salehi Zahabi, Hosni Salem, Marwa R. Rashad Salem, Yahya Salimi, Hamideh Salimzadeh, Hossein Samadi Kafil, Evanson Zondani Sambala, Abdallah M. Samy, Itamar S. Santos, Bruno Piassi Sao Jose, Sivan Yegnanarayana Iyer Saraswathy, Abdur Razzaque Sarker, Benn Sartorius, Arash Sarveazad, Brijesh Sathian, Maheswar Satpathy, Sonia Saxena, Mehdi Sayyah, Alyssa N. Sbarra, Megan F. Schipp, Maria Inês Schmidt, Aletta Elisabeth Schutte, David C. Schwebel, Anbissa Muleta Senbeta, Subramanian Senthilkumaran, Seyedmojtaba Seyedmousavi, Faramarz Shaahmadi, Omid Shafaat, Saeed Shahabi, Masood Ali Shaikh, Ali S. Shalash, Mehran Shams-Beyranvand, Amir Shamshirian, Morteza Shamsizadeh, Mohammed Shannawaz, Kiomars Sharafi, Mehdi Sharif, Rajesh Sharma, Hatem Samir Shehata, Abbas Sheikhtaheri, Kenji Shibuya, Wondimeneh Shibabaw Shiferaw, Mika Shigematsu, Jae Il Shin, Rahman Shiri, Reza Shirkoohi, Ivy Shiue, Kerem Shuval, Soraya Siabani, Tariq J. Siddiqi, Inga Dora Sigfusdottir, Diego Augusto Santos Silva, Biagio Simonetti, Ambrish Singh, Pushpendra Singh, Virendra Singh, Jasvinder A. Singh, Pankaj Kumar Singh, Dhirendra Narain Sinha, Yitagesu Sintayehu, Malede Mequanent M. Sisay, Amin Soheili, Bija Soleymani, Farzaneh Soltani, Shahin Soltani, Joan B. Soriano, Muluken Bekele Sorrie, Sergey Soshnikov, Ireneous N. Soyiri, Adel Spotin, Chandrashekhar T. Sreeramareddy, Rajni Kant Kant Srivastava, Antonina Starodubova, Agus Sudaryanto, Mu’awiyyah Babale Sufiyan, Hafiz Ansar Rasul Suleria, Gerhard Sulo, Bruno F. Sunguya, Bryan L. Sykes, Rafael Tabarés-Seisdedos, Takahiro Tabuchi, Birkneh Tilahun Tadesse, Amir Taherkhani, Koku Sisay Tamirat, Segen Gebremeskel Tassew, Nuno Taveira, Berhane Fseha Teklehaimanot, Gebretsadkan Hintsa Tekulu, Mohamad-Hani Temsah, Abdullah Sulieman Terkawi, Zemenu Tadesse Tessema, Nihal Thomas, Mariya Vladimirovna Titova, Kenean Getaneh Tlaye, Hamid Reza Tohidinik, Marcello Tonelli, Marcos Roberto Tovani-Palone, Eugenio Traini, Khanh Bao Tran, Manjari Tripathi, Riaz Uddin, Irfan Ullah, Bhaskaran Unnikrishnan, Era Upadhyay, Ushotanefe Useh, Muhammad Shariq Usman, Olalekan A. Uthman, Marco Vacante, Masoud Vaezghasemi, Pascual R. Valdez, John VanderHeide, Elena Varavikova, Santosh Varughese, Tommi Juhani Vasankari, Yasser Vasseghian, Yousef Veisani, Srinivasaraghavan Venkatesh, Narayanaswamy Venketasubramanian, Madhur Verma, Simone Vidale, Francesco S. Violante, Vasily Vlassov, Sebastian Vollmer, Rade Vukovic, Yasir Waheed, Haidong Wang, Yafeng Wang, Yuan-Pang Wang, Girmay Teklay Weldesamuel, Andrea Werdecker, Taweewat Wiangkham, Kirsten E. Wiens, Tissa Wijeratne, Haileab Fekadu Wolde, Dawit Zewdu Wondafrash, Tewodros Eshete Wonde, Adam Belay Wondmieneh, Ai-Min Wu, Gelin Xu, Abbas Yadegar, Ali Yadollahpour, Seyed Hossein Yahyazadeh Jabbari, Tomohide Yamada, Yuichiro Yano, Sanni Yaya, Vahid Yazdi-Feyzabadi, Alex Yeshaneh, Yigizie Yeshaw, Yordanos Gizachew Yeshitila, Mekdes Tigistu Yilma, Paul Yip, Naohiro Yonemoto, Seok-Jun Yoon, Yoosik Youm, Mustafa Z. Younis, Zabihollah Yousefi, Hebat-Allah Salah A. Yousof, Chuanhua Yu, Hasan Yusefzadeh, Telma Zahirian Moghadam, Leila Zaki, Sojib Bin Zaman, Mohammad Zamani, Maryam Zamanian, Hamed Zandian, Hadi Zarafshan, Nejimu Biza Zepro, Taddese Alemu Zerfu, Taye Abuhay Zewale, Yunquan Zhang, Zhi-Jiang Zhang, Xiu-Ju Zhao, Sanjay Zodpey, Kamiar Zomorodian, Francis Bruno Zotor, Ashkan Afshin, Simon I. Hay

**Affiliations:** 1 Institute for Health Metrics and Evaluation, University of Washington, Seattle, WA USA; 2 Department of Health Metrics Sciences, School of Medicine, University of Washington, Seattle, WA USA; 3 Department of Global Health, University of Washington, Seattle, WA USA; 4 Department of Medicine, University of Washington, Seattle, WA USA; 5 School of Nutrition and Food Sciences, Tabriz University of Medical Sciences, Tabriz, Iran; 6 Kermanshah University of Medical Sciences, Kermanshah, Iran; 7 Advanced Diagnostic and Interventional Radiology Research Center, Tehran University of Medical Sciences, Tehran, Iran; 8 Department of Neurology, Cairo University, Cairo, Egypt; 9 Department of Parasitology and Mycology, Jahrom University of Medical Sciences, Jahrom, Iran; 10 The Institute of Pharmaceutical Sciences (TIPS), Toxicology and Diseases Group, Tehran University of Medical Sciences, Tehran, Iran; 11 Neuroscience Research Center, Isfahan University of Medical Sciences, Isfahan, Iran; 12 Department of Public Health, Ministry of Health, Riyadh, Saudi Arabia; 13 School of Public Health, Addis Ababa University, Addis Ababa, Ethiopia; 14 Public Health, Wachemo University, Hosanna, Ethiopia; 15 College of Medicine and Health Sciences, Hawassa University, Hawassa, Ethiopia; 16 Biostatistics and Health Informatics, Madda Walabu University, Bale Robe, Ethiopia; 17 Radiotherapy Center, Addis Ababa University, Addis Ababa, Ethiopia; 18 LABMED, Karolinska University Hospital, Huddinge, Sweden; 19 Research Center for Immunodeficiencies, Tehran University of Medical Sciences, Tehran, Iran; 20 Department of Pediatric Dentistry, Federal University of Minas Gerais, Belo Horizonte, Brazil; 21 Research Department, Philippine Institute for Development Studies, Quezon City, Philippines; 22 Cardiovascular Medicine, Ain Shams University, Abbasia, Egypt; 23 Bénin Clinical Research Institute (IRCB), Cotonou, Benin; 24 Department of Preventive Medicine, Dongguk University, Gyeongju, South Korea; 25 Department of Community Medicine, Kathmandu University, Devdaha, Nepal; 26 Hamadan University of Medical Sciences, Hamadan, Iran; 27 Department of Community Medicine, University of Ibadan, Ibadan, Nigeria; 28 Department of Community Medicine, University College Hospital, Ibadan, Ibadan, Nigeria; 29 Department of Sociology, Olabisi Onabanjo University, Ago-Iwoye, Nigeria; 30 School of Medicine, Cardiff University, Cardiff, UK; 31 College of Medicine, University of Ibadan, Ibadan, Nigeria; 32 Community Cardiovascular Research Unit, Elyon Heart Rehabilitation Center, Ibadan, Nigeria; 33 Department of Global Health, Stellenbosch University, Stellenbosch, South Africa; 34 Cochrane South Africa, South African Medical Research Council, Cape Town, South Africa; 35 School of Health, Ardabil University of Medical Science, Ardabil, Iran; 36 Department of Health Promotion and Education, University of Ibadan, Ibadan, Nigeria; 37 Social Behavioral Research Branch, National Institute of Health, Bethesda, MD USA; 38 Cancer Prevention and Control, Georgetown University, Washington, DC USA; 39 Endocrinology and Metabolism Research Center (EMRC), Tehran University of Medical Sciences, Tehran, Iran; 40 Epidemiology, Qom University of Medical Sciences, Qom, Iran; 41 Research Area for Informatics and Big Data, CSIR Institute of Genomics and Integrative Biology, Delhi, India; 42 Department of Internal Medicine, Baylor College of Medicine, Houston, TX USA; 43 Department of Epidemiology and Health Statistics, School of Public Health, Southeast University Nanjing, Nanjing, China; 44 Microbiology Department, Hazara University Mansehra, Mansehra, Pakistan; 45 Lincoln Medical School, Universities of Nottingham & Lincoln, Lincoln, UK; 46 School of Advanced Technologies in Medicine, Shahid Beheshti University of Medical Sciences, Tehran, Iran; 47 Department of Epidemiology, Jimma University, Jimma, Ethiopia; 48 James P Grant School of Public Health, BRAC University, Dhaka, Bangladesh; 49 Health Systems and Population Studies Division, International Centre for Diarrhoeal Disease Research, Bangladesh, Dhaka, Bangladesh; 50 School of Community Health Sciences, University of Nevada, Reno, NV USA; 51 National Malaria Elimination Program, Federal Ministry of Health, Abuja, Nigeria; 52 Department of Medical Laboratory Sciences, Bahir Dar University, Bahir Dar, Ethiopia; 53 Department of Epidemiology and Biostatistics, University of Gondar, Gondar, Ethiopia; 54 Department of Population Health Sciences, Duke University, Durham, NC USA; 55 Duke Global Health Institute, Duke University, Durham, NC USA; 56 School of Public Health and Community Medicine, University of New South Wales, Sydney, New South Wales Australia; 57 John T. Milliken Department of Internal Medicine, Washington University in St. Louis, St Louis, MO USA; 58 Clinical Epidemiology Center, VA Saint Louis Health Care System, Department of Veterans Affairs, St Louis, MO USA; 59 Department of Medicine, Dalhousie University, Halifax, NS Canada; 60 School of Health Sciences, Madda Walabu University, Bale Goba, Ethiopia; 61 Department of Health Information Management and Technology, Imam Abdulrahman Bin Faisal University, Dammam, Saudi Arabia; 62 Centre of Health System Research, National Institute of Public Health, Cuernavaca, Mexico; 63 Department of Pharmacy, Adigrat University, Adigrat, Ethiopia; 64 Medical Technical Institute, Erbil Polytechnic University, Erbil, Iraq; 65 Ishik University, Erbil, Iraq; 66 Department of Biotechnology, Quaid-i-Azam University Islamabad, Islamabad, Pakistan; 67 Social Determinants of Health Research Center, Qazvin University of Medical Sciences, Qazvin, Iran; 68 Department of Health Care Management and Economics, Urmia University of Medical Science, Urmia, Iran; 69 Health Management and Economics Research Center, Iran University of Medical Sciences, Tehran, Iran; 70 Health Economics Department, Iran University of Medical Sciences, Tehran, Iran; 71 Department of Microbiology, Kerman University of Medical Sciences, Kerman, Iran; 72 Department of Microbiology, Hormozgan University of Medical Sciences, Bandar Abbas, Iran; 73 Department of Health Policy and Management, Kuwait University, Safat, Kuwait; 74 International Centre for Casemix and Clinical Coding, National University of Malaysia, Bandar Tun Razak, Malaysia; 75 Research Center for Environmental Determinants of Health (RCEDH), Kermanshah University of Medical Sciences, Kermanshah, Iran; 76 Department of Epidemiology, Arak University of Medical Sciences, Arak, Iran; 77 Medical Research Center, Jazan University, Jazan, Saudi Arabia; 78 Department of Medical Parasitology, Sana’a University, Sana’a, Yemen; 79 Department of Family and Community Medicine, King Abdulaziz University, Jeddah, Saudi Arabia; 80 King Saud University, Riyadh, Saudi Arabia; 81 Research Group in Health Economics, University of Cartagena, Cartagena, Colombia; 82 Research Group in Hospital Management and Health Policies, University of the Coast, Barranquilla, Colombia; 83 Departamento de Ciencias Económicas, Universidad de la Costa, Barranquilla, Colombia; 84 Observatorio Nacional de Salud, National Institute of Health, Bogotá, Colombia; 85 Sansom Institute, South Australian Health and Medical Research Institute, Adelaide, South Australia Australia; 86 Bahir Dar University, Bahir Dar, Ethiopia; 87 Biomedical Science, University of Cape Coast, Cape Coast, Ghana; 88 Health Services Management Department, Arak University of Medical Sciences, Arak, Iran; 89 Health Services Management, Isfahan University of Medical Sciences, Isfahan, Iran; 90 Department of Radiology, Kermanshah University of Medical Sciences, Kermanshah, Iran; 91 Department of Epidemiology and Biostatistics, University of the Philippines Manila, Manila, Philippines; 92 Online Programs for Applied Learning, Johns Hopkins University, Baltimore, MD USA; 93 Mansoura University, Mansoura, Egypt; 94 Carol Davila University of Medicine and Pharmacy, Bucharest, Romania; 95 Research Center for Evidence Based Medicine-Health Management and Safety Promotion Research Institute, Tabriz University of Medical Sciences, Tabriz, Iran; 96 Razi Vaccine and Serum Research Institute, Agricultural Research Education and Extension Organization (AREEO), Tehran, Iran; 97 Department of Epidemiology and Biostatistics, Health Promotion Research Center, Zahedan, Iran; 98 Department of Epidemiology and Biostatistics, Bahir Dar University, Bahir Dar, Ethiopia; 99 Department of Health Policy and Administration, University of the Philippines Manila, Manila, Philippines; 100 Department of Applied Social Sciences, Hong Kong Polytechnic University, Hong Kong, China; 101 Department of Agribusiness, Universitas Sebelas Maret, Surakarta, Indonesia; 102 Department of Parasitology, Mazandaran University of Medical Sciences, Sari, Iran; 103 Department of Microbiology and Immunology, Iranshahr University of Medical Sciences, Iranshahr, Iran; 104 Department of Pathology, Al-Imam Mohammad Ibn Saud Islamic University, Riyadh, Saudi Arabia; 105 Department of Sociology and Social Work, Kwame Nkrumah University of Science and Technology, Kumasi, Ghana; 106 Center for International Health, Ludwig Maximilians University, Munich, Germany; 107 Social Determinants of Health Research Center, Birjand University of Medical Sciences, Birjand, Iran; 108 Department of Health Promotion and Education, Tehran University of Medical Sciences, Tehran, Iran; 109 School of Health Sciences, Birmingham City University, Birmingham, UK; 110 Department of Neurobiology, Karolinska Institutet, Stockholm, Sweden; 111 School of Health and Social Studies, Dalarna University, Falun, Sweden; 112 School of Nursing and Midwife, Babol University of Medical Sciences, Babol, Iran; 113 Babol University of Medical Sciences, Babol, Iran; 114 Preventive Medicine and Public Health Research Center, Iran University of Medical Sciences, Tehran, Iran; 115 Neurology, Shiraz University of Medical Sciences, Shiraz, Iran; 116 Prevention of Metabolic Disorders Research Center, Shahid Beheshti University of Medical Sciences, Tehran, Iran; 117 Department of Microbiology, Hamedan University of Medical Sciences, Hamedan, Iran; 118 Department of Clinical Chemistry, University of Gondar, Gondar, Ethiopia; 119 Non-Communicable Diseases Research Center, Tehran University of Medical Sciences, Tehran, Iran; 120 Department of Nursing, Aksum University, Aksum, Ethiopia; 121 Department of Health System and Health Economics, Bahir Dar University, Bahir Dar City, Ethiopia; 122 School of Nursing, , University of Nottingham, Amman, Jordan; 123 School of Business, University of Leicester, Leicester, UK; 124 Department of Statistics and Econometrics, Bucharest University of Economic Studies, Bucharest, Romania; 125 Bénin Clinical Research Institute (IRCB), Abomey-Calavi, Benin; 126 Contrôle des Maladies Infectieuses, Laboratory of Studies and Research-Action in Health, Porto Novo, Benin; 127 Indian Institute of Public Health, Public Health Foundation of India, Gurugram, India; 128 The Judith Lumley Centre, La Trobe University, Melbourne, Victoria Australia; 129 General Office for Research and Technological Transfer, Peruvian National Institute of Health, Lima, Peru; 130 Department of Health Policy Planning and Management, University of Health and Allied Sciences, Ho, Ghana; 131 Department of Nursing, Debre Berhan University, Debre Berhan, Ethiopia; 132 Cellular and Molecular Biology Research Center, Babol University of Medical Sciences, Babol, Iran; 133 Department of Environmental Health Engineering, Hamadan University of Medical Sciences, Hamadan, Iran; 134 Department of Reproductive Health, University of Gondar, Gondar, Ethiopia; 135 Public Health Risk Sciences Division, Public Health Agency of Canada, Toronto, Ontario Canada; 136 Department of Nutritional Sciences, University of Toronto, Toronto, Ontario Canada; 137 Department of Forensic Science, Government Institute of Forensic Science, Nagpur, India; 138 Healthcare Management Department, Shiraz University of Medical Sciences, Shiraz, Iran; 139 Biochemistry Unit, Universiti Sultan Zainal Abidin, Kuala Terengganu, Malaysia; 140 Biomedicine Department, Universiti Sultan Zainal Abidin Gongbedak, Kuala Terengganu, Malaysia; 141 Health Policy and Management Department, Tehran University of Medical Sciences, Tehran, Iran; 142 Department of Forensic Medicine and Toxicology, , Manipal Academy of Higher Education, Manipal, India; 143 Department of Medical Laboratory Science, Haramaya University, Harar, Ethiopia; 144 School of Public Health, Haramaya University, Harar, Ethiopia; 145 Department of Hypertension, Medical University of Lodz, Lodz, Poland; 146 Polish Mothers’ Memorial Hospital Research Institute, Lodz, Poland; 147 Department of Noncommunicable Diseases, Bangladesh University of Health Sciences (BUHS), Dhaka, Bangladesh; 148 Department of Animal Pathology and Epidemiology, Razi Vaccine and Serum Research Institute, Karaj, Iran; 149 Department of Neurosciences, Costa Rican Department of Social Security, San Jose, Costa Rica; 150 School of Medicine, University of Costa Rica, San Pedro, Costa Rica; 151 Heidelberg Institute of Global Health (HIGH), Heidelberg University, Heidelberg, Germany; 152 T.H. Chan School of Public Health, Harvard University, Boston, MA USA; 153 University of Aden, Aden, Yemen; 154 School of Public Health, Imperial College London, London, UK; 155 Health Human Resources Research Center, Shiraz University of Medical Sciences, Shiraz, Iran; 156 Department of Applied Human Nutrition, Bahir Dar University, Bahir Dar, Ethiopia; 157 University of Gondar, Gondar, Ethiopia; 158 Department of Community Medicine, Gandhi Medical College Bhopal, Bhopal, India; 159 Jazan University, Jazan, Saudi Arabia; 160 Social Determinants of Health Research Center, Lorestan University of Medical Sciences, Khorramabad, Iran; 161 Department of Epidemiology and Biostatistics, Lorestan University of Medical Sciences, Khorramabad, Iran; 162 Department of Reproductive Health and Population Studies, Bahir Dar University, Bahir Dar, Ethiopia; 163 Nuffield Department of Population Health, University of Oxford, Oxford, UK; 164 Department of Public Health, Arba Minch University, Arba Minch, Ethiopia; 165 Department of Nutrition and Dietetics, Mekelle University, Mekelle, Ethiopia; 166 Adigrat University, Adigrat, Ethiopia; 167 School of Public Health, Wolaita Sodo University, Addis Ababa, Ethiopia; 168 Department of Medicine, Medical College of Georgia at Augusta University, Augusta, GA USA; 169 Hubert Department of Global Health, Emory University, Atlanta, GA USA; 170 Department of Global Health, University of South Florida, Tampa, FL USA; 171 Department of Health Information Management, Manipal Academy of Higher Education, Manipal, Manipal, India; 172 School of Public Health, University of Adelaide, Adelaide, South Australia Australia; 173 Public Health Research Laboratory, Institute of Medicine, Tribhuvan University, Kathmandu, Nepal; 174 Department of Community Medicine and Family Medicine, All India Institute of Medical Sciences, Jodhpur, India; 175 Department of Community Medicine, Datta Meghe Institute of Medical Sciences, Deemed University, Wardha, India; 176 Department of Statistical and Computational Genomics, National Institute of Biomedical Genomics, Kalyani, India; 177 Department of Statistics, University of Calcutta, Kolkata, India; 178 Social Determinants of Health Research Center, Babol University of Medical Sciences, Babol, Iran; 179 Istituto di Ricerche Farmacologiche Mario Negri IRCCS, Ranica, Italy; 180 Health Economics & Outcomes Research, Creativ-Ceutical (Huntsworth Health), London, UK; 181 Woldia University, Woldia, Ethiopia; 182 National Centre for Epidemiology and Population Health, Australian National University, Canberra, Australian Capital Territory Australia; 183 Department of Clinical Pharmacy and Pharmacology, University of Dhaka, Dhaka, Bangladesh; 184 Department of Public Health, Samara University, Samara, Ethiopia; 185 Debretabor University, Addis Ababa University, Debretabor, Ethiopia; 186 Department of Pediatrics and Child Health Nursing, Bahir Dar University, Bahir Dar, Ethiopia; 187 Transport and Road Safety (TARS) Research Center, , University of New South Wales, Sydney, New South Wales Australia; 188 School of Health Sciences, Swinburne University of Technology, Melbourne, Victoria Australia; 189 Department of Nutrition, Saint Paul’s Hospital Millennium Medical College, Addis Ababa, Ethiopia; 190 Department of Veterinary Medicine, Islamic Azad University, Kermanshah, Iran; 191 Department of Biomedical Sciences, Nazarbayev University, Nur-Sultan City, Kazakhstan; 192 Department of Internal Medicine, Manipal Academy of Higher Education, Mangalore, India; 193 Department of General Surgery and Medical-Surgical Specialties, University of Catania, Catania, Italy; 194 School of Medicine, Hamadan University of Medical Sciences, Hamadan, Iran; 195 Department of Infectious Disease Epidemiology, London School of Hygiene & Tropical Medicine, London, UK; 196 University of Genoa, Genoa, Italy; 197 Division of Hematology and Oncology, Georgetown University, Washington, DC USA; 198 Epidemiology and Evidence Based Medicine, I.M. Sechenov First Moscow State Medical University, Moscow, Russia; 199 Gorgas Memorial Institute for Health Studies, Panama City, Panama; 200 Department of Research, Golden Community, Kathmandu, Nepal; 201 Department of Community Medicine, Employees’ State Insurance Model Hospital, Bangalore, India; 202 Department for Health Care Management, Technical University of Berlin, Berlin, Germany; 203 School of Public Health and Health Systems, University of Waterloo, Waterloo, Ontario Canada; 204 Al Shifa School of Public Health, Al Shifa Trust Eye Hospital, Rawalpindi, Pakistan; 205 Internal Medicine Department, Hospital Italiano de Buenos Aires, Ciudad Autónoma de Buenos Aires, Buenos Aires, Argentina; 206 Comisión Directiva, Argentine Society of Medicine, Ciudad Autónoma de Buenos Aires, Buenos Aires, Argentina; 207 National Institute of Public Health, Cuernavaca, Mexico; 208 Department of Disease Control, London School of Hygiene & Tropical Medicine, London, UK; 209 Centre for Population Health Sciences, Nanyang Technological University, Singapore, Singapore; 210 Global eHealth Unit, Imperial College London, London, UK; 211 Department of Population and Health, Metropolitan Autonomous University, Mexico City, Mexico; 212 Department of Medical Epidemiology and Biostatistics, Karolinska Institutet, Stockholm, Sweden; 213 Research Unit on Applied Molecular Biosciences (UCIBIO), University of Porto, Porto, Portugal; 214 Department of Psychiatry, University of São Paulo, São Paulo, Brazil; 215 Colombian National Health Observatory, National Institute of Health, Bogota, Colombia; 216 Epidemiology and Public Health Evaluation Group, National University of Colombia, Bogota, Colombia; 217 Mary MacKillop Institute for Health Research, Australian Catholic University, Melbourne, Victoria Australia; 218 School of Public Health, University of Hong Kong, Hong Kong, China; 219 Health, Nutrition and Population, World Bank, Lusaka, Zambia; 220 Institute for Global Health, Heidelberg University, Heidelberg, Germany; 221 Department of Pharmacology, All India Institute of Medical Sciences, Jodhpur, India; 222 Division of Epidemiology, National Institute of Cholera and Enteric Diseases, Kolkata, India; 223 Department of Medicine, University of Toronto, Toronto, Ontario Canada; 224 Population Research Centre, Gokhale Institute of Politics and Economics, Pune, India; 225 International Institute for Population Sciences, Mumbai, India; 226 Department of Medical Entomology and Vector Control, Urmia University of Medical Science, Urmia, Iran; 227 Department of Biostatistics and Epidemiology, Babol University of Medical Sciences, Babol, Iran; 228 Epidemiology Research Center, Royan Institute, Tehran, Iran; 229 Department of Nursing, Wolaita Sodo University, Sodo, Ethiopia; 230 Department of Epidemiology and Preventive Medicine, Monash University, Melbourne, Victoria Australia; 231 Department of Pulmonary Medicine, Christian Medical College and Hospital (CMC), Vellore, India; 232 Hanoi National University of Education, Hanoi, Vietnam; 233 School of Public Health and Preventive Medicine, Monash University, Melbourne, Victoria Australia; 234 School of Medicine and Surgery, University of Milan Bicocca, Monza, Italy; 235 Institute of Public Health, University of Gondar, Gondar, Ethiopia; 236 Discipline of Public Health, Flinders University, Adelaide, South Australia Australia; 237 Department of Human Physiology, University of Gondar, Gondar, Ethiopia; 238 Department of Environmental Health, Imam Abdulrahman Bin Faisal University, Dammam, Saudi Arabia; 239 Department of Dermatology, Case Western Reserve University, Cleveland, OH USA; 240 Department of Dermatology, University of Milan, Milan, Italy; 241 Department of Pediatrics, Tanta University, Tanta, Egypt; 242 Toxoplasmosis Research Center, Mazandaran University of Medical Sciences, Sari, Iran; 243 Division of Women and Child Health, Aga Khan University, Karachi, Pakistan; 244 Department of Epidemiology and Biostatistics, Arnold School of Public Health, University of South Carolina, Columbia, SC USA; 245 Population and Development, Facultad Latinoamericana de Ciencias Sociales Mexico, Mexico City, Mexico; 246 Australian Institute for Suicide Research and Prevention, Griffith University, Mount Gravatt, Queensland Australia; 247 Department of Nursing, Woldia University, Woldia, Ethiopia; 248 Department of Nursing, Jimma University, Jimma, Ethiopia; 249 Department of Neonatal Nursing, St. Paul’s Hospital Millennium Medical College, Addis Ababa, Ethiopia; 250 Ambo University, Ambo, Ethiopia; 251 School of Pharmacy, Aksum University, Aksum, Ethiopia; 252 Addis Ababa University,, Addis Ababa, Ethiopia; 253 Center for Nutrition and Health Research, National Institute of Public Health, Cuernavaca, Mexico; 254 Department of Global Health and Infection, Brighton and Sussex Medical School, Brighton, UK; 255 Division of Cardiology, Atlanta Veterans Affairs Medical Center, Decatur, GA USA; 256 School of Nutrition, Food Science and Technology, Hawassa University, Hawassa, Ethiopia; 257 School of Nursing and Midwifery, Haramaya University, Harar, Ethiopia; 258 Centre for Atmospheric Sciences, Indian Institute of Technology Delhi, New Delhi, India; 259 Department of Community Medicine, University of Peradeniya, Peradeniya, Sri Lanka; 260 Mathematical Demography and Statistics, International Institute for Population Sciences, Mumbai, India; 261 Health Research Section, Nepal Health Research Council, Kathmandu, Nepal; 262 Department of Microbiology, Far Western University, Mahendranagar, Nepal; 263 Department of Epidemiology, Shiraz University of Medical Sciences, Shiraz, Iran; 264 Center of Complexity Sciences, National Autonomous University of Mexico, Mexico City, Mexico; 265 Facultad de Medicina Veterinaria y Zootecnia, Autonomous University of Sinaloa, Culiacan, Mexico; 266 Department of Nursing, Bank Melli, Tehran, Iran; 267 Fenot Project, Harvard University, Addis Ababa, Ethiopia; 268 Ministry of Health and Medical Education, Tehran, Iran; 269 Center of Excellence in Public Health Nutrition, Nguyen Tat Thanh University, Ho Chi Minh, Vietnam; 270 Center of Excellence in Behavioral Medicine, Nguyen Tat Thanh University, Ho Chi Minh City, Vietnam; 271 School of Nursing and Midwifery, University of Cape Coast, Cape Coast, Ghana; 272 Iran University of Medical Sciences, Tehran, Iran; 273 Department of Health Policy and Economy, Tabriz University of Medical Sciences, Tabriz, Iran; 274 World Food Programme, New Delhi, India; 275 Public Health Department, Hawassa University, Hawassa, Ethiopia; 276 Curtin University, Perth, Western Australia Australia; 277 Centre for Tropical Medicine and Global Health, University of Oxford, Oxford, UK; 278 Mahidol-Oxford Tropical Medicine Research Unit, Bangkok, Thailand; 279 Postgraduate Program in Epidemiology, Federal University of Rio Grande do Sul, Porto Alegre, Brazil; 280 School of Medicine, Federal University of Bahia, Salvador, Brazil; 281 Medicina Interna, Escola Bahiana de Medicina e Saúde Pública, Salvador, Brazil; 282 Department of Bacteriology and Virology, Tabriz University of Medical Sciences, Tabriz, Iran; 283 Department of Pharmacology and Toxicology, Maragheh University of Medical Sciences, Maragheh, Iran; 284 Department of Pharmacology and Toxicology, Tabriz University of Medical Sciences, Tabriz, Iran; 285 Biomedical Informatics and Medical Statistics, Alexandria University, Alexandria, Egypt; 286 Department of Clinical Pathology, Mansoura University, Mansoura, Egypt; 287 Pediatric Dentistry and Dental Public Health, Alexandria University, Alexandria, Egypt; 288 Institute of Public Health, United Arab Emirates University, Al Ain, United Arab Emirates; 289 Department of Statistics, Debre Markos University, Debre Markos, Ethiopia; 290 Department of Public Health Sciences, Karolinska Institutet, Stockholm, Sweden; 291 World Health Programme, Université du Québec en Abitibi-Témiscamingue, Rouyn-Noranda, Quebec Canada; 292 Endemic Medicine and Hepatogastroentrology Department, Cairo University, Cairo, Egypt; 293 Department of Biosciences, Nottingham Trent University, Nottingham, UK; 294 Eijkman-Oxford Clinical Research Unit, Eijkman Institute for Molecular Biology, Jakarta, Indonesia; 295 Ophthalmic Epidemiology Research Center, Shahroud University of Medical Sciences, Shahroud, Iran; 296 Department of Microbiology and Immunology, Suez Canal University, Ismailia, Egypt; 297 Department of Midwifery, Wolkite University, Wolkite, Ethiopia; 298 Department of Midwifery, Woldia University, Woldia, Ethiopia; 299 Department of Medicinal Chemistry, Kerman University of Medical Sciences, Kerman, Iran; 300 Pharmaceutics Research Center, Kerman University of Medical Sciences, Kerman, Iran; 301 Multiple Sclerosis Research Center, Tehran University of Medical Sciences, Tehran, Iran; 302 Department of Physiology, Tarbiat Modares University, Tehran, Iran; 303 Division of Cancer Epidemiology and Genetics, National Cancer Institute, Bethesda, MD USA; 304 Tehran University of Medical Sciences, Tehran, Iran; 305 Unit of Medical Physiology, Hawassa University, Hawassa, Ethiopia; 306 Berman Institute of Bioethics, Johns Hopkins University, Baltimore, MD USA; 307 Nutrition and Food Systems Division, Food and Agriculture Organization of the United Nations, Rome, Italy; 308 School of Public Health, Tehran University of Medical Sciences, Tehran, Iran; 309 Department of Political Science, University of Human Development, Sulaimaniyah, Iraq; 310 Deputy of Research and Technology, Hamadan University of Medical Sciences, Hamadan, Iran; 311 College of Medicine, Imam Mohammad Ibn Saud Islamic University, Riyadh, Saudi Arabia; 312 Department of Medical and Surgical Sciences, University of Bologna, Bologna, Italy; 313 Department of Psychology, Federal University of Sergipe, Sao Cristovao, Brazil; 314 Department of Biological and Biomedical Sciences, Aga Khan University, Karachi, Pakistan; 315 College of Medicine and Public Health, Flinders University, Adelaide, South Australia Australia; 316 Institute of Resource Governance and Social Change, Kupang, Indonesia; 317 Social Determinants of Health Research Center, Hamadan University of Medical Sciences, Hamadan, Iran; 318 Department of Public Health Nutrition, Bahir Dar University, Bahir Dar, Ethiopia; 319 School of Nursing and Midwifery, Hawassa University, Hawassa, Ethiopia; 320 Division of Neurology, University of Ottawa, Ottawa, Ontario Canada; 321 REQUIMTE/LAQV - Network of Chemistry and Technology, University of Porto, Porto, Portugal; 322 Center for Biotechnology and Fine Chemistry, Catholic University of Portugal, Porto, Portugal; 323 Department of Health Education & Behavioral Sciences, Jimma University, Jimma, Ethiopia; 324 Jimma University, Jimma, Ethiopia; 325 Psychiatry Department, Kaiser Permanente, Fontana, CA USA; 326 School of Health Sciences, A.T. Still University, Mesa, AZ USA; 327 Department of Population Medicine and Health Services Research, Bielefeld University, Bielefeld, Germany; 328 Unit for Population-Based Dermatology Research, King’s College London, London, UK; 329 Institute of Gerontology, National Academy of Medical Sciences of Ukraine, Kyiv, Ukraine; 330 Department of Child Dental Health, Obafemi Awolowo University, Ile-Ife, Nigeria; 331 Timiryazev Institute of Plant Physiology (IPPRAS), Russian Academy of Sciences, Moscow, Russia; 332 Abadan School of Medical Sciences, Abadan University of Medical Sciences, Abadan, Iran; 333 Department of Research, Center for Population and Health, Wiesbaden, Germany; 334 Department of Family Medicine and Primary Care, University of the Witwatersrand, Johannesburg, South Africa; 335 Department of Dermatology, Kobe University, Kobe, Japan; 336 Gene Expression & Regulation Program, The Wistar Institute, Philadelphia, PA USA; 337 School of Nursing and Midwifery, Wollega University, Nekemte, Ethiopia; 338 Public Health Department, Madda Walabu University, Bale-Robe, Ethiopia; 339 School of Public Health, Mekelle University, Mekelle, Ethiopia; 340 Department of Nursing and Midwifery, Addis Ababa University, Addis Ababa, Ethiopia; 341 Nursing Department, Mekelle University, Mekelle, Ethiopia; 342 Haramaya University, Dire Dawa, Ethiopia; 343 Pharmacy, Wollo University, Dessie, Ethiopia; 344 Department of Nursing, Arba Minch University, Arba Minch, Ethiopia; 345 Department of Biostatistics, Mekelle University, Mekelle, Ethiopia; 346 Department of Parasitology and Entomology, Tarbiat Modares University, Tehran, Iran; 347 Department of Medical Surgery, Tabriz University of Medical Sciences, Tabriz, Iran; 348 Department of Medicine, Massachusetts General Hospital, Boston, MA USA; 349 Neuroscience Institute, Academy of Medical Science, Tehran, Iran; 350 Department of Health Services Management, Iran University of Medical Sciences, Tehran, Iran; 351 Social Determinants of Health Research Center, University of Social Welfare and Rehabilitation Sciences, Tehran, Iran; 352 Science and Research Branch, Islamic Azad University, Tehran, Iran; 353 Young Researchers and Elite Club, Islamic Azad University, Rasht, Iran; 354 University of Lahore, Lahore, Pakistan; 355 Afro-Asian Institute, Lahore, Pakistan; 356 Adelaide Medical School, University of Adelaide, Adelaide, South Australia Australia; 357 Department of Family and Community Medicine, University of Hail, Hail, Saudi Arabia; 358 Center for the Study of Regional Development, Jawahar Lal Nehru University, New Delhi, India; 359 Department of Chemistry, University of Porto, Porto, Portugal; 360 Department of Biostatistics and Epidemiology, University of Oklahoma, Oklahoma City, OK USA; 361 Department of Health and Social Affairs, Government of the Federated States of Micronesia, Palikir, Federated States of Micronesia; 362 Department of Respiratory Medicine, Hokkaido University, Sapporo, Japan; 363 Center for Environmental and Health Sciences, Hokkaido University, Sapporo, Japan; 364 Center for Clinical and Epidemiological Research, University of São Paulo, Sao Paulo, Brazil; 365 Internal Medicine Department, University of São Paulo, São Paulo, Brazil; 366 Manipal Institute of Virology, Manipal Academy of Higher Education, Manipal, India; 367 Department of Dermatology, Boston University, Boston, MA USA; 368 Instituto de Patologia Tropical e Saúde Pública, Federal University of Goiás, Goiânia, Brazil; 369 College of Medicine and Health Science, Jigjiga University, Jigjiga, Ethiopia; 370 Department of Epidemiology and Biostatistics, Zhengzhou University, Zhengzhou, China; 371 March of Dimes, Arlington, VA USA; 372 School of Public Health, West Virginia University Morgantown, Morgantown, WV USA; 373 Academics and Research Department, Rajasthan University of Health Sciences, Jaipur, India; 374 Department of Medicine, Mahatma Gandhi University of Medical Sciences & Technology, Jaipur, India; 375 Department of Radiology and Radiological Sciences, Johns Hopkins University, Baltimore, MD USA; 376 School of Medicine, Tehran University of Medical Sciences, Tehran, Iran; 377 Department of Nursing, St. Paul’s Hospital Millennium Medical College, Addis Ababa, Ethiopia; 378 Department of Pharmacology, Tehran University of Medical Sciences, Tehran, Iran; 379 Obesity Research Center, Shahid Beheshti University of Medical Sciences, Tehran, Iran; 380 Global and Community Mental Health Research Group, University of Macau, Macao, China; 381 Department of Anatomical Sciences, Tarbiat Modares University, Tehran, Iran; 382 Department of Family and Community Medicine, Arabian Gulf University, Manama, Bahrain; 383 Department of Health Management and Economics, Hamadan University of Medical Sciences, Hamadan, Iran; 384 School of Medicine, University of Western Australia, Perth, Western Australia Australia; 385 Neurology Department, Sir Charles Gairdner Hospital, Perth, Western Australia Australia; 386 Tabriz University of Medical Sciences, Tabriz, Iran; 387 Department of Dental Public Health, Universitas Airlangga Indonesia, Surabaya, Indonesia; 388 Australian Research Centre for Population Oral Health, University of Adelaide, Adelaide, South Australia Australia; 389 Department of Zoology, Al-Azhar University, Cairo, Egypt; 390 Institute for Social Science Research, The University of Queensland, Indooroopilly, Queensland Australia; 391 Department of Healthcare Management, Maragheh University of Medical Sciences, Maragheh, Iran; 392 Department of Microbiology, Maragheh University of Medical Sciences, Maragheh, Iran; 393 Department of Microbiology, Tehran University of Medical Sciences, Tehran, Iran; 394 Department of Biology, Utica College, Utica, NY USA; 395 Gastrointestinal and Liver Disease Research Center, Guilan University of Medical Sciences, Rasht, Iran; 396 Guilan University of Medical Sciences, Rasht, Iran; 397 Department of Public Health, Mizan-Tepi University, Tepi, Ethiopia; 398 Unit of Epidemiology and Social Medicine, University Hospital Antwerp, Wilrijk, Belgium; 399 Department of Clinical Sciences, Karolinska University Hospital, Stockholm, Sweden; 400 School of Health Sciences, City University of London, London, UK; 401 Institute of Pharmaceutical Sciences, University of Veterinary and Animal Sciences, Lahore, Pakistan; 402 Department of Pharmacy Administration and Clinical Pharmacy, Xian Jiaotong University, Xian, China; 403 Shahrekord University of Medical Sciences, Shahrekord, Iran; 404 School of Public Health, Curtin University, Perth, Western Australia Australia; 405 Agriculture and Food, Commonwealth Scientific and Industrial Research Organisation, St. Lucia, Queensland Australia; 406 Medical Biology Research Center, Kermanshah University of Medical Sciences, Kermanshah, Iran; 407 Department of Biostatistics and Epidemiology, Adigrat University, Adigrat, Ethiopia; 408 Department of Psychiatry, University Medical Center Groningen, Groningen, the Netherlands; 409 Department of Epidemiology, Columbia University, New York, NY USA; 410 Department of Pediatrics, Dell Medical School, University of Texas Austin, Austin, TX USA; 411 Kasturba Medical College, Manipal Academy of Higher Education, Manipal, India; 412 Guilan Road Trauma Research Center, Guilan University of Medical Sciences, Rasht, Iran; 413 Social Determinants of Health Research Center, Guilan University of Medical Sciences, Rasht, Iran; 414 Department of Pediatrics, Yonsei University, Seoul, South Korea; 415 Research Department, Electronic Medical Records for the Developing World, York, UK; 416 Transdisciplinary Centre for Qualitative Methods, Manipal Academy of Higher Education, Manipal, India; 417 Nevada Division of Public and Behavioral Health, Carson City, NV USA; 418 Department of Pharmacology and Therapeutics, Dhaka Medical College, Dhaka, Bangladesh; 419 Department of Pharmacology, Bangladesh Industrial Gases Limited, Tangail, Bangladesh; 420 Department of Epidemiology and Biostatistics, Tehran University of Medical Sciences, Tehran, Iran; 421 Department of Computer Engineering, Islamic Azad University, Tehran, Iran; 422 Computer Science Department, University of Human Development, Sulaymaniyah, Iraq; 423 Department of General Surgery, Carol Davila University of Medicine and Pharmacy, Bucharest, Romania; 424 Department of Internal Medicine, Bucharest Emergency Hospital, Bucharest, Romania; 425 Faculty of Dentistry, Department of Legal Medicine and Bioethics, Carol Davila University of Medicine and Pharmacy, Bucharest, Romania; 426 Clinical Legal Medicine Department, National Institute of Legal Medicine, Bucharest, Romania; 427 College of Science and Engineering, Hamad Bin Khalifa University, Doha, Qatar; 428 Medicine School of Tunis, Baab Saadoun, Tunisia; 429 Department of Epidemiology and Health Statistics, Central South University, Changsha, China; 430 School of Public Health, University of Sydney, Sydney, New South Wales Australia; 431 Maternal and Child Health Division, International Centre for Diarrhoeal Disease Research, Dhaka, Bangladesh; 432 Department of Public Health and Community Medicine, Shaikh Khalifa Bin Zayed Al-Nahyan Medical College at Shaikh Zayed Medical Complex, Lahore, Pakistan; 433 Department of Occupational Safety and Health, China Medical University, Taichung, Taiwan; 434 Department of Epidemiology, University of Kragujevac, Kragujevac, Serbia; 435 Department of Public Health, Lorestan University of Medical Sciences, Khorramabad, Iran; 436 Department of Family Medicine, Bangalore Baptist Hospital, Bangalore, India; 437 Global Health and Development Department, Taipei Medical University, Taipei City, Taiwan; 438 Research Institute for Endocrine Sciences, Shahid Beheshti University of Medical Sciences, Tehran, Iran; 439 Institute for Physical Activity and Nutrition, Deakin University, Burwood, Victoria Australia; 440 Sydney Medical School, University of Sydney, Sydney, New South Wales Australia; 441 School of Health Systems and Public Health, University of Pretoria, Hatfield, South Africa; 442 Cochrane Center, South African Medical Research Council, Parow Valley, South Africa; 443 Health Systems and Public Health, Stellenbosch University, Cape Town, South Africa; 444 Department of Epidemiology, Shahid Beheshti University of Medical Sciences, Tehran, Iran; 445 Department of Environmental Health Engineering, Guilan University of Medical Sciences, Rasht, Iran; 446 Medical Research Council South Africa, Cape Town, South Africa; 447 Centre for Evidence Based Health Care, Stellenbosch University, Cape Town, South Africa; 448 Department of Immunology, Tabriz University of Medical Sciences, Tabriz, Iran; 449 Department of Psychosis, Babol Noshirvani University of Technology, Babol, Iran; 450 Department of Immunology, Isfahan University of Medical Sciences, Isfahan, Iran; 451 Department for Health Care and Public Health, Sechenov First Moscow State Medical University, Moscow, Russia; 452 Department of Psychiatry, Kermanshah University of Medical Sciences, Kermanshah, Iran; 453 Social Development & Health Promotion Research Center, Kermanshah University of Medical Sciences, Kermanshah, Iran; 454 Kermanshah University of Medical Sciences, Kermanshah, Iran; 455 Institute of Medicine, University of Colombo, Colombo, Sri Lanka; 456 University of Colombo, Colombo, Sri Lanka; 457 Achutha Menon Centre for Health Science Studies, Sree Chitra Tirunal Institute for Medical Sciences and Technology, Trivandrum, India; 458 Department of Pediatrics & Child Health, Aga Khan University, Karachi, Pakistan; 459 Autism Spectrum Disorders Research Center, Hamadan University of Medical Sciences, Hamadan, Iran; 460 Department of Community Medicine, Banaras Hindu University, Varanasi, India; 461 Manipal Academy of Higher Education, Manipal, India; 462 The George Institute for Global Health, University of New South Wales, New Delhi, India; 463 Environmental Research Center, Duke Kunshan University, Kunshan, China; 464 Nicholas School of the Environment, Duke University, Durham, NC USA; 465 Department of Earth Observation Science, University of Twente, Enschede, the Netherlands; 466 Department of Ophthalmology, Heidelberg University, Mannheim, Germany; 467 Beijing Ophthalmology & Visual Science Key Laboratory, Beijing Tongren Hospital, Beijing, China; 468 Department of Community Medicine, Manipal Academy of Higher Education, Mangalore, India; 469 Department of Family Medicine and Public Health, University of Opole, Opole, Poland; 470 School of Health Sciences, Savitribai Phule Pune University, Pune, India; 471 Institute of Family Medicine and Public Health, University of Tartu, Tartu, Estonia; 472 Minimally Invasive Surgery Research Center, Iran University of Medical Sciences, Tehran, Iran; 473 School of Public Health, University College Cork, Cork, UK; 474 Infectious Diseases Research Center, Golestan University of Medical Sciences, Gorgan, Iran; 475 Department of Medical Informatics, Tabriz University of Medical Sciences, Tabriz, Iran; 476 Health Services Management Department, School of Health Qazvin University of Medical Sciences Qazvin, Qazvin, Iran; 477 Community Medicine Department, Rafsanjan University of Medical Sciences, Iran, Rafsanjan, Iran; 478 Department of Forensic Medicine and Toxicology, All India Institute of Medical Sciences, Jodhpur, India; 479 All India Institute of Medical Sciences, New Delhi, India; 480 Department of Epidemiology, Hamadan University of Medical Sciences, Hamadan, Iran; 481 Institute for Epidemiology and Social Medicine, University of Münster, Münster, Germany; 482 Research and Development, Australian Red Cross Blood Service, Sydney, New South Wales Australia; 483 Hematology-Oncology and Stem Cell Transplantation Research Center, Tehran University of Medical Sciences, Tehran, Iran; 484 Pars Advanced and Minimally Invasive Medical Manners Research Center, Iran University of Medical Sciences, Tehran, Iran; 485 Clinical Pharmacy Unit, Mekelle University, Mekelle, Ethiopia; 486 Department of Anesthesiology & Pain Medicine, University of Washington, Seattle, WA USA; 487 Department of Public Health, Kermanshah University of Medical Sciences, Kermanshah, Iran; 488 Non-Communicable Diseases Research Unit, Medical Research Council South Africa, Cape Town, South Africa; 489 Department of Medicine, University of Cape Town, Cape Town, South Africa; 490 Department of Public Health, Debre Markos University, Debre Markos, Ethiopia; 491 Department of Public Health, Jordan University of Science and Technology, Irbid, Jordan; 492 Social Determinants of Health Research Center, Ahvaz Jundishapur University of Medical Sciences, Ahvaz, Iran; 493 Department of Physiology, Lorestan University of Medical Sciences, Khorramabad, Iran; 494 School of Food and Agricultural Sciences, University of Management and Technology, Lahore, Pakistan; 495 Department of Physiology, Baku State University, Baku, Azerbaijan; 496 School of Health and Rehabilitation Sciences, The University of Queensland, Brisbane, Queensland Australia; 497 Epidemiology and Biostatistics Department, Health Services Academy, Islamabad, Pakistan; 498 Department of Population Sciences, Jatiya Kabi Kazi Nazrul Islam University, Mymensingh, Bangladesh; 499 Department of Public Health, University of Newcastle, Newcastle, New South Wales Australia; 500 Department of Hospital Medicine, Miriam Hospital, Brown University, Providence, RI USA; 501 Department of Internal Medicine, John H. Stroger, Jr. Hospital of Cook County, Chicago, IL USA; 502 Department of Internal Medicine, Dow University of Health Sciences, Karachi, Pakistan; 503 Faculty of Health and Wellbeing, Sheffield Hallam University, Sheffield, UK; 504 College of Arts and Sciences, Ohio University, Zanesville, OH USA; 505 Internal Medicine and Gastroenterology Department, National Hepatology and Tropical Research Institute, Cairo, Egypt; 506 Department of Medical Parasitology, Cairo University, Cairo, Egypt; 507 Division of Evidence Synthesis, Datta Meghe Institute of Medical Sciences, Wardha, India; 508 Cancer Research Center, Shahid Beheshti University of Medical Sciences, Tehran, Iran; 509 Academy of Medical Science, Tehran, Iran; 510 Department of Public Health, Mazandaran University of Medical Sciences, Sari, Iran; 511 Department of Biostatistics, Shahid Beheshti University of Medical Sciences, Tehran, Iran; 512 Department of Neurosurgery, Iran University of Medical Sciences, Tehran, Iran; 513 Oxford University Global Surgery Group, University of Oxford, Oxford, UK; 514 Clinical Epidemiology Unit, Lund University, Lund, Sweden; 515 Research and Data Solutions, Synotech Consultant, Nairobi, Kenya; 516 School of Medicine, Xiamen University Malaysia, Sepang, Malaysia; 517 Department of Nutrition, Simmons University, Boston, MA USA; 518 School of Health Sciences, Kristiania University College, Oslo, Norway; 519 Department of Nursing and Health Promotion, Oslo Metropolitan University, Oslo, Norway; 520 Department of Public Health, Ambo University, Ambo, Ethiopia; 521 Neurophysiology Research Center, Hamadan University of Medical Sciences, Hamadan, Iran; 522 Brain Engineering Research Center, Institute for Research in Fundamental Sciences, Tehran, Iran; 523 Department of Public Health Dentistry, Deemed University, Karad, India; 524 Department of Environmental Health Engineering, Arak University of Medical Sciences, Arak, Iran; 525 Department of Internal and Pulmonary Medicine, , Sheri Kashmir Institute of Medical Sciences, Srinagar, India; 526 CIBERSAM, San Juan de Dios Sanitary Park, Sant Boi de Llobregat, Spain; 527 Department of Zoology, University of Oxford, Oxford, UK; 528 Harvard Medical School, Harvard University, Boston, MA USA; 529 Department of Anthropology, Panjab University, Chandigarh, India; 530 Department of Family and Community Health, University of Health and Allied Sciences, Ho, Ghana; 531 Department of Psychology and Health Promotion, University of KwaZulu-Natal, Durban, South Africa; 532 Department of Psychiatry, University of Nairobi, Nairobi, Kenya; 533 Division of Psychology and Language Sciences, University College London, London, UK; 534 Department of Medicine Brigham and Women’s Hospital, Harvard University, Boston, MA USA; 535 Department of Pathology and Molecular Medicine, McMaster University, Hamilton, Ontario Canada; 536 Institute of Occupational and Environmental Medicine, University of Birmingham, Birmingham, UK; 537 Health and Nutrition Section, United Nations Childrens’ Fund (UNICEF), Accra, Ghana; 538 Clinical Medicine and Community Health, University of Milan, Milano, Italy; 539 National Institute for Health Research (NIHR), Oxford Biomedical Research Centre, Oxford, UK; 540 Department of Internal Medicine, Post Graduate Institute of Medical Education and Research, Chandigarh, India; 541 Public Health Foundation of India, Gurugram, India; 542 Department of Community and Family Medicine, University of Baghdad, Baghdad, Iraq; 543 School of Medicine, Deakin University, Geelong, Victoria Australia; 544 Health Promotion and Chronic Disease Prevention Branch, Public Health Agency of Canada, Ottawa, Ontario Canada; 545 HelpMeSee, New York, NY USA; 546 International Relations, Mexican Institute of Ophthalmology, Queretaro, Mexico; 547 Department of Otorhinolaryngology (ENT) & Head and Neck Surgery, Father Muller Medical College, Mangalore, India; 548 Department of Information and Internet Technologies, I.M. Sechenov First Moscow State Medical University, Moscow, Russia; 549 Federal Research Institute for Health Organization and Informatics of the Ministry of Health (FRIHOI), Moscow, Russia; 550 School of Nursing, Hong Kong Polytechnic University, Hong Kong, China; 551 School of Pharmacy, Monash University, Bandar Sunway, Malaysia; 552 School of Pharmacy, Taylor’s University Lakeside Campus, Subang Jaya, Malaysia; 553 Oxford University Clinical Research Unit, Wellcome Trust Asia Programme, Hanoi, Vietnam; 554 Department of Medicine, University of Malaya, Kuala Lumpur, Malaysia; 555 Department of Medicine and Therapeutics, The Chinese University of Hong Kong, Hong Kong, China; 556 School of Public Health, University of Haifa, Haifa, Israel; 557 Centre for Chronic Disease Control, Beijing, China; 558 Department of Epidemiology, Brown University, Providence, RI USA; 559 Department of Paediatrics, All India Institute of Medical Sciences, New Delhi, India; 560 Vector Biology, Liverpool School of Tropical Medicine, Liverpool, UK; 561 Department of Nutrition, University of the Philippines Manila, Manila, Philippines; 562 Alliance for Improving Health Outcomes, Inc., Quezon City, Philippines; 563 Institute of Nutrition, Friedrich Schiller University Jena, Jena, Germany; 564 Competence Cluster for Nutrition and Cardiovascular Health (nutriCARD), Jena, Germany; 565 Ariadne Labs, Harvard University, Boston, MA USA; 566 Development and Communication Studies, University of the Philippines Los Baños, Laguna, Philippines; 567 Pathology Department, Imam Abdulrahman Bin Faisal University, Dammam, Saudi Arabia; 568 Radiology Department, Mansoura University Hospital, Mansoura, Egypt; 569 Ophthalmology Department, Aswan Faculty of Medicine, Aswan, Egypt; 570 Department of Internal Medicine, Grant Medical College & Sir J.J. Group of Hospitals, Mumbai, India; 571 Institute of Medicine, Tribhuvan University, Kathmandu, Nepal; 572 Health Education and Research Department, SDM College of Medical Sciences & Hospital, Dharwad, India; 573 Health University, Rajiv Gandhi University of Health Sciences, Bangalore, India; 574 Environmental Health Research Center, Kurdistan University of Medical Sciences, Sanandaj, Iran; 575 Clinical Research Development Center, Kermanshah University of Medical Sciences, Kermanshah, Iran; 576 Department of Maternal and Child Nursing and Public Health, Federal University of Minas Gerais, Belo Horizonte, Brazil; 577 Plastic Surgery Department, Iran University of Medical Sciences, Tehran, Iran; 578 Joint Centre for Bioethics, University of Toronto, Toronto, Ontario Canada; 579 Ophthalmology Department, Iran University of Medical Sciences, Tehran, Iran; 580 Ophthalmology Department, University of Manitoba, Winnipeg, Manitoba Canada; 581 School of Science and Health, Western Sydney University, Sydney, New South Wales Australia; 582 Substance Abuse Prevention Research Center, Kermanshah University of Medical Sciences, Kermanshah, Iran; 583 Department of Population Studies, University of Zambia, Lusaka, Zambia; 584 Research Department, Grupo de Investigación Fundovida - Fundovida IPS, Cartagena, Colombia; 585 Grupo de Investigación en Economía de la Salud, University of Cartagena, Cartagena, Colombia; 586 Campus Caucaia, Federal Institute of Education, Science and Technology of Ceará, Caucaia, Brazil; 587 Public Health Department, Botho University-Botswana, Gaborone, Botswana; 588 Division of Plastic Surgery, University of Washington, Seattle, WA USA; 589 Research Department, The George Institute for Global Health, New Delhi, India; 590 School of Medicine, University of New South Wales, Sydney, New South Wales Australia; 591 ICF International, DHS Program, Rockville, MD USA; 592 Department of Twin Research and Genetic Epidemiology, King’s College London, London, UK; 593 Neurology Department, Janakpuri Super Specialty Hospital Society, New Delhi, India; 594 Neurology Department, Govind Ballabh Institute of Medical Education and Research, New Delhi, India; 595 Pharmacology and Toxicology, Hamadan University of Medical Sciences, Hamadan, Iran; 596 Department of Epidemiology and Biostatistics, University of California San Francisco, San Francisco, CA USA; 597 Public Health and Mortality, International Institute for Population Sciences, Mumbai, India; 598 Department of Nutrition, University of Oslo, Oslo, Norway; 599 Mekelle University, Mekelle, Ethiopia; 600 Peru Country Office, United Nations Population Fund (UNFPA), Lima, Peru; 601 Forensic Medicine Division, Imam Abdulrahman Bin Faisal University, Dammam, Saudi Arabia; 602 Department of Midwifery, Adigrat University, Adigrat, Ethiopia; 603 Center for Translation Research and Implementation Science, National Institutes of Health, Bethesda, MD USA; 604 Breast Surgery Unit, Helsinki University Hospital, Helsinki, Finland; 605 University of Helsinki, Helsinki, Finland; 606 Department of Propedeutics of Internal Diseases & Arterial Hypertension, Pomeranian Medical University, Szczecin, Poland; 607 Health Policy and Management, Centre for Regional Policy Research and Cooperation ‘Studiorum’, Skopje, Macedonia; 608 Pacific Institute for Research & Evaluation, Calverton, MD USA; 609 Department of Health Research Methods, Evidence and Impact, McMaster University, Hamilton, Ontario Canada; 610 Global Institute of Public Health (GIPH), Ananthapuri Hospitals and Research Centre, Trivandrum, India; 611 Department of Clinical Biochemistry, Babol University of Medical Sciences, Babol, Iran; 612 Golestan University of Medical Sciences, Gorgan, Iran; 613 Department of Environmental Health, Sabzevar University of Medical Sciences, Sabzevar, Iran; 614 Foodborne and Waterborne Diseases Research Center, Research Institute for Gastroenterology and Liver Diseases, Shahid Beheshti University of Medical Sciences, Tehran, Iran; 615 Kyrgyz State Medical Academy, Bishkek, Kyrgyzstan; 616 Department of Atherosclerosis and Coronary Heart Disease, National Center of Cardiology and Internal Disease, Bishkek, Kyrgyzstan; 617 Research Center for Biochemistry and Nutrition in Metabolic Diseases, Kashan University of Medical Sciences, Kashan, Iran; 618 Department of Rehabilitation and Sports Medicine, Kermanshah University of Medical Sciences, Kermanshah, Iran; 619 Deputy of Social Health, Iran University of Medical Sciences, Tehran, Iran; 620 Health Equity Research Center, Tehran University of Medical Sciences, Tehran, Iran; 621 Social Determinants of Health Research Center, Kurdistan University of Medical Sciences, Sanandaj, Iran; 622 Research Center, Salahaddin University, Erbil, Iraq; 623 Internal Medicine Department, King Saud University, Riyadh, Saudi Arabia; 624 Department of Food Technology, Salahaddin University, Erbil, Iraq; 625 Department of Medicine, Karolinska Institutet, Stockholm, Sweden; 626 Department of Information Technology, University of Human Development, Sulaymaniyah, Iraq; 627 Department of Biostatistics, Hamadan University of Medical Sciences, Hamadan, Iran; 628 Department of Epidemiology and Biostatistics, Shahrekord University of Medical Sciences, Shahrekord, Iran; 629 Department of Immunology, Babol University of Medical Sciences, Babol, Iran; 630 Clinical Biochemistry, Tarbiat Modares University, Tehran, Iran; 631 Department of Nursing, Shahroud University of Medical Sciences, Shahroud, Iran; 632 Department of Biomolecular Sciences, University of Mississippi, Oxford, MS USA; 633 Department of Pharmacy, Mizan-Tepi University, Mizan, Ethiopia; 634 Health Systems and Policy Research Unit, Ahmadu Bello University, Zaria, Nigeria; 635 School of Pharmacy, Haramaya University, Harar, Ethiopia; 636 Iran National Institute of Health Research, Tehran University of Medical Sciences, Tehran, Iran; 637 Community Nutrition, Shahid Beheshti University of Medical Sciences, Tehran, Iran; 638 Health Systems Research Center, National Health Research Institutes, Cuernavaca, Mexico; 639 Department of Public Health Sciences, University of Miami, Miami, FL USA; 640 Department of Public Health Medicine, University of KwaZulu-Natal, Durban, South Africa; 641 Department of Molecular Medicine, Birjand University of Medical Sciences, Birjand, Iran; 642 Department of Epidemiology and Biostatistics, Kurdistan University of Medical Sciences, Sanandaj, Iran; 643 Department of Epidemiology, Iran University of Medical Sciences, Tehran, Iran; 644 Department of Economics and Management Sciences for Health, Tehran University of Medical Sciences, Tehran, Iran; 645 Department of Mathematical Sciences, University of Bath, Bath, UK; 646 Department of Surgery, University of Washington, Seattle, WA USA; 647 Department of Clinical Biochemistry, Tarbiat Modares University, Tehran, Iran; 648 Food Science, University of Campinas, Campinas, Brazil; 649 Friedman School of Nutrition Science and Policy, Tufts University, Boston, MA USA; 650 Federal Institute for Population Research, Wiesbaden, Germany; 651 Center for Population and Health, Wiesbaden, Germany; 652 Indian Institute of Public Health - Hyderabad, Public Health Foundation of India, Hyderabad, India; 653 School of Medical Sciences, Science University of Malaysia, Kubang Kerian, Malaysia; 654 Department of Pediatric Medicine, Nishtar Medical University, Multan, Pakistan; 655 Department of Pediatrics & Pediatric Pulmonology, Institute of Mother & Child Care, Multan, Pakistan; 656 Department of Microbiology and Immunology, Mekelle University, Mekelle, Ethiopia; 657 Department of Urology, Tehran University of Medical Sciences, Tehran, Iran; 658 Department of Medicine, Icahn School of Medicine at Mount Sinai, New York, NY USA; 659 Research and Analytics, Initiative for Financing Health and Human Development, Chennai, India; 660 Research and Analytics, Bioinsilico Technologies, Chennai, India; 661 Initiative for Non Communicable Diseases, International Centre for Diarrhoeal Disease Research, Dhaka, Bangladesh; 662 Comprehensive Cancer Center, University of Alabama at Birmingham, Birmingham, AL USA; 663 Department of Epidemiology & Biostatistics, Kermanshah University of Medical Sciences, Kermanshah, Iran; 664 Department of Disease, Epidemics, and Pandemics Control, Ministry of Public Health, Yaoundé, Cameroon; 665 Department of Public Heath, University of Yaoundé I, Yaoundé, Cameroon; 666 Hospital of the Federal University of Minas Gerais, Federal University of Minas Gerais, Belo Horizonte, Brazil; 667 Department of Pediatrics, Arak University of Medical Sciences, Arak, Iran; 668 Iranian Ministry of Health and Medical Education, Tehran, Iran; 669 General Surgery, Emergency Hospital of Bucharest, Bucharest, Romania; 670 Anatomy and Embryology, Carol Davila University of Medicine and Pharmacy, Bucharest, Romania; 671 Cardiology, Cardio-Aid, Bucharest, Romania; 672 Department of Biological Sciences, University of Embu, Embu, Kenya; 673 Institute for Global Health Innovations, Duy Tan University, Hanoi, Vietnam; 674 Institute of Mental Health Research, University of Ottawa, Ottawa, Ontario Canada; 675 Department of Clinical Epidemiology, Institute for Clinical Evaluative Sciences, Ottawa, Ontario Canada; 676 Department of Pharmacology of Tehran University of Medical Sciences, Shahid Beheshti University of Medical Sciences, Tehran, Iran; 677 Heidelberg University Hospital, Heidelberg, Germany; 678 Public Health Department, Universitas Negeri Semarang, Kota Semarang, Indonesia; 679 Graduate Institute of Biomedical Informatics, Taipei Medical University, Taipei City, Taiwan; 680 School of Public Health and Family Medicine, University of Cape Town, Cape Town, South Africa; 681 Department of Neurobiology, Care Sciences and Society (NVS), H1, Division of Family Medicine and Primary Care, Karolinska Institutet, Huddinge, Sweden; 682 Administrative and Economic Sciences, University of Bucharest, Bucharest, Romania; 683 Centre of Cardiovascular Research and Education in Therapeutics, Monash University, Melbourne, Victoria Australia; 684 Independent Consultant, Accra, Ghana; 685 Department Obstetrics and Gynecology, University of Ibadan, Ibadan, Nigeria; 686 Department of Preventive Medicine, Kyung Hee University, Dongdaemun-gu, South Korea; 687 HAST, Human Sciences Research Council, Durban, South Africa; 688 School of Public Health, University of Namibia, Osakhati, Namibia; 689 Department of Medical Genetics, School of Advanced Technologies in Medicine, Golestan University of Medical Sciences, Gorgan, Iran; 690 Department of Psychiatry and Behavioural Neurosciences, McMaster University, Hamilton, Ontario Canada; 691 Department of Psychiatry, University of Lagos, Lagos, Nigeria; 692 Centre for Healthy Start Initiative, Lagos, Nigeria; 693 Centre for Healthy Start Initiative, Phonics Hearing Centre, Lagos, Nigeria; 694 Public Health and School of Graduates Studies, Jigjiga University, Jig-Jiga, Ethiopia; 695 Department of Pharmacology and Therapeutics, University of Nigeria Nsukka, Enugu, Nigeria; 696 Department of Psychology, University of Ghana, Accra, Ghana; 697 Graduate School of Public Health, San Diego State University, San Diego, CA USA; 698 University of Washington, Seattle, WA USA; 699 University of Port Harcourt, Port Harcourt, Nigeria; 700 School of Medicine, Autonomous University of Madrid, Madrid, Spain; 701 Department of Nephrology and Hypertension, The Institute for Health Research Foundation Jiménez Díaz University Hospital, Madrid, Spain; 702 Department of Environmental Management and Toxicology, University of Benin, Benin City, Nigeria; 703 Institute for Advanced Medical Research and Training, University of Ibadan, Ibadan, Nigeria; 704 Department of Respiratory Medicine, Jagadguru Sri Shivarathreeshwara Academy of Health Education and Research, Mysore, India; 705 Department of Forensic Medicine and Toxicology, Manipal Academy of Higher Education, Mangalore, India; 706 Department of Medical Mycology and Parasitology, Shiraz University of Medical Sciences, Shiraz, Iran; 707 Center for Health Outcomes & Evaluation, Bucharest, Romania; 708 Augenpraxis Jonas, Heidelberg University, Heidelberg, Germany; 709 Internal Medicine, University of Pittsburgh Medical Center, Pittsburgh, PA USA; 710 Research and Evaluation, Population Council, New Delhi, India; 711 Indian Institute of Health Management Research University, Jaipur, India; 712 Department of Pediatircs, RD Gardi Medical College, Ujjain, India; 713 Public Health Sciences, Karolinska Institutet, Stockholm, Sweden; 714 Research & Publication Cell, Kalinga Institute of Medical Sciences, Bhubaneswar, Bhubaneswar, India; 715 Regional Medical Research Centre, Indian Council of Medical Research, Bhubaneswar, India; 716 Department of Population Studies, International Institute for Population Sciences, Mumbai, India; 717 International Institute of Health Management Research, New Delhi, India; 718 Department of Paediatrics, University of Melbourne, Melbourne, Victoria Australia; 719 Population Health, Murdoch Childrens Research Institute, Melbourne, Victoria Australia; 720 Wolaita Sodo University, Sodo, Ethiopia; 721 Department of Physiology, Iran University of Medical Sciences, Tehran, Iran; 722 Center for Research and Innovation, Ateneo De Manila University, Pasig City, Philippines; 723 Istituto di Ricerche Farmacologiche Mario Negri IRCCS, Bergamo, Italy; 724 School of Medicine, University of Virginia, Charlottesville, VA USA; 725 HIV and Mental Health Department, Integrated Development Foundation Nepal, Kathmandu, Nepal; 726 University Medical Center Groningen, University of Groningen, Groningen, the Netherlands; 727 Faculty of Economics and Business, University of Groningen, Groningen, the Netherlands; 728 Department of Public Health, Maragheh University of Medical Sciences, Maragheh, Iran; 729 Department of Nutrition and Food Sciences, Maragheh University of Medical Sciences, Maragheh, Iran; 730 School of Population and Public Health, University of British Columbia, Vancouver, British Columbia Canada; 731 Paramedic Department, Kermanshah University of Medical Sciences, Kermanshah, Iran; 732 Digestive Diseases Research Institute, Tehran University of Medical Sciences, Tehran, Iran; 733 Fundación Valle del Lili, Cali, Colombia; 734 Infectious Diseases, National Institute of Infectious Diseases, Bucuresti, Romania; 735 Department of Infectious Diseases, Carol Davila University of Medicine and Pharmacy, Bucharest, Romania; 736 Health Sciences Department, Muhammadiyah University of Surakarta, Sukoharjo, Indonesia; 737 Biomedical Engineering Department, Amirkabir University of Technology, Tehran, Iran; 738 Department of Chemistry, Sharif University of Technology, Tehran, Iran; 739 College of Medicine, University of Central Florida, Orlando, FL USA; 740 College of Graduate Health Sciences, A.T. Still University, Mesa, AZ USA; 741 Department of Immunology, Mazandaran University of Medical Sciences, Sari, Iran; 742 Molecular and Cell Biology Research Center, Mazandaran University of Medical Sciences, Sari, Iran; 743 Thalassemia and Hemoglobinopathy Research Center, Ahvaz Jundishapur University of Medical Sciences, Ahvaz, Iran; 744 Metabolomics and Genomics Research Center, Tehran University of Medical Sciences, Tehran, Iran; 745 Sina Trauma and Surgery Research Center, Tehran University of Medical Sciences, Tehran, Iran; 746 School of Nursing and Healthcare Professions, Federation University, Heidelberg, Victoria Australia; 747 National Centre for Farmer Health, Deakin University, Waurn Ponds, Victoria Australia; 748 Department of Clinical Pediatrics, Sweidi Hospital, Riyadh, Saudi Arabia; 749 Department of Pediatrics, North-West University, Peshawar, Pakistan; 750 Society for Health and Demographic Surveillance, Suri, India; 751 Department of Economics, University of Göttingen, Göttingen, Germany; 752 Birjand University of Medical Sciences, Birjand, Iran; 753 Department of Pharmacology, Shahid Beheshti University of Medical Sciences, Tehran, Iran; 754 University Institute of Public Health, University of Lahore, Lahore, Pakistan; 755 Public Health Department, University of Health Sciences, Lahore, Pakistan; 756 Policy Research Institute, Kathmandu, Nepal; 757 Institute for Poverty Alleviation and International Development, Yonsei University, Wonju, South Korea; 758 Department of Oral Pathology, Srinivas Institute of Dental Sciences, Mangalore, India; 759 Gonçalo Moniz Institute, Oswaldo Cruz Foundation, Salvador, Brazil; 760 Institute of Public Health, Federal University of Bahia, Salvador, Brazil; 761 School of Behavioral Sciences and Mental Health, Tehran Institute of Psychiatry, Tehran, Iran; 762 Kasturba Medical College, Manipal Academy of Higher Education, Mangalore, India; 763 Department of Primary Care and Public Health, Imperial College London, London, UK; 764 Academic Public Health Department, Public Health England, London, UK; 765 WHO Collaborating Centre for Public Health Education and Training, Imperial College London, London, UK; 766 University College London Hospitals, London, UK; 767 School of Health, Medical and Applied Sciences, Central Queensland University, Sydney, New South Wales Australia; 768 Neurology Department, Sree Chitra Tirunal Institute for Medical Sciences and Technology, Thiruvananthapuram, India; 769 School of Social Sciences and Psychology, Western Sydney University, Penrith, New South Wales Australia; 770 Translational Health Research Institute, Western Sydney University, Penrith, New South Wales Australia; 771 Brien Holden Vision Institute, Sydney, New South Wales Australia; 772 Organization for the Prevention of Blindness, Paris, France; 773 Network of Immunity in Infection, Malignancy and Autoimmunity (NIIMA), Universal Scientific Education and Research Network (USERN), Tehran, Iran; 774 Pediatric Infectious Diseases Research Center, Mazandaran University of Medical Sciences, Sari, Iran; 775 Department of Epidemiology, Birjand University of Medical Sciences, Birjand, Iran; 776 EPIUnit - Public Health Institute University Porto (ISPUP), University of Porto, Porto, Portugal; 777 Surgery Department, University of Minnesota, Minneapolis, MN USA; 778 Surgery Department, University Teaching Hospital of Kigali, Kigali, Rwanda; 779 School of Psychology, University of Lincoln, Lincoln, UK; 780 Department of Epidemiology and Biostatistics, Imperial College London, London, UK; 781 Department of Clinical Research, Federal University of Uberlândia, Uberlândia, Brazil; 782 Department of Public Health, Wollega University, Nekemte, Ethiopia; 783 Public Health Department, Addis Ababa University, Addis Ababa, Ethiopia; 784 Golestan Research Center of Gastroenterology and Hepatology, Golestan University of Medical Sciences, Gorgan, Iran; 785 Infectious Diseases and Tropical Medicine Research Center, Babol University of Medical Sciences, Babol, Iran; 786 Centro de Investigación Palmira, Agrosavia, Palmira, Colombia; 787 Department of Ocean Science and Engineering, Southern University of Science and Technology, Shenzhen, China; 788 Ain Shams University, Cairo, Egypt; 789 Department of Cardiology, Tehran University of Medical Sciences, Tehran, Iran; 790 National Institute for Research in Environmental Health, Indian Council of Medical Research, Bhopal, India; 791 Cardiovascular Research Institute, Isfahan University of Medical Sciences, Isfahan, Iran; 792 Emergency Department, Shahid Beheshti University of Medical Sciences, Tehran, Iran; 793 Department of Health in Disasters and Emergencies, Shahid Beheshti University of Medical Sciences, Tehran, Iran; 794 Department of Psychiatry, All India Institute of Medical Sciences, New Delhi, India; 795 Halal Research Center of IRI, FDA, Tehran, Iran; 796 Neurogenic Inflammation Research Center, Mashhad University of Medical Sciences, Mashhad, Iran; 797 Nanobiotechnology Center, Soran University, Soran, Iraq; 798 Department of Anatomical Sciences, Kermanshah University of Medical Sciences, Kermanshah, Iran; 799 Department of Pathology, Imam Mohammad Ibn Saud Islamic University, Riyadh, Saudi Arabia; 800 Taleghani Hospital, Kermanshah University of Medical Sciences, Kermanshah, Iran; 801 Radiology and Nuclear Medicine Department, Kermanshah University of Medical Sciences, Kermanshah, Iran; 802 Taleghani Hospital, Kermanshah, Iran; 803 Urology Department, Cairo University, Cairo, Egypt; 804 Public Health and Community Medicine, Cairo University, Giza, Egypt; 805 Drug Applied Research Center, Tabriz University of Medical Sciences, Tabriz, Iran; 806 Department of Entomology, Ain Shams University, Cairo, Egypt; 807 Department of Internal Medicine, University of São Paulo, São Paulo, Brazil; 808 Department of Infectious Diseases and Tropical Medicine, Federal University of Minas Gerais, Belo Horizonte, Brazil; 809 Department of Community Medicine, PSG Institute of Medical Sciences and Research, Coimbatore, India; 810 PSG-FAIMER, South Asia Regional Institute, Coimbatore, India; 811 Health Economics and Financing Research Group, International Centre for Diarrhoeal Disease Research, Bangladesh, Dhaka, Bangladesh; 812 Faculty of Infectious and Tropical Diseases, London School of Hygiene & Tropical Medicine, London, UK; 813 Colorectal Research Center, Iran University of Medical Sciences, Tehran, Iran; 814 Surgery Department, Hamad General Hospital, Hamad Medical Corporation, Doha, Qatar; 815 Faculty of Health & Social Sciences, Bournemouth University, Bournemouth, UK; 816 UGC Centre of Advanced Study in Psychology, Utkal University, Bhubaneswar, India; 817 Udyam-Global Association for Sustainable Development, Bhubaneswar, India; 818 Hypertension in Africa Research Team (HART), North-West University, Potchefstroom, South Africa; 819 Unit for Hypertension and Cardiovascular Disease, South African Medical Research Council, Cape Town, South Africa; 820 Department of Psychology, University of Alabama at Birmingham, Birmingham, AL USA; 821 Department of Food Science and Nutrition, Jigjiga University, Jigjiga, Ethiopia; 822 Emergency Department, Manian Medical Centre, Erode, India; 823 Microbiology Service, National Institutes of Health, Bethesda, MD USA; 824 Department of Health Promotion and Education, Alborz University of Medical Sciences, Karaj, Iran; 825 Health Policy Research Center, Shiraz University of Medical Sciences, Shiraz, Iran; 826 Independent Consultant, Karachi, Pakistan; 827 Department of Neuropsychiatry, Ain Shams University, Cairo, Egypt; 828 School of Medicine, Alborz University of Medical Sciences, Karaj, Iran; 829 Medical Laboratory Sciences, Mazandaran University of Medical Sciences, Sari, Iran; 830 Chronic Diseases (Home Care) Research Center, , Hamadan University of Medical Sciences, Hamadan, Iran; 831 Department of Development Studies, International Institute for Population Sciences, Mumbai, India; 832 Department of Basic Sciences, Islamic Azad University, Sari, Iran; 833 Department of Laboratory Sciences, Islamic Azad University, Sari, Iran; 834 University School of Management and Entrepreneurship, Delhi Technological University, New Delhi, India; 835 Department of Health Information Management and Informatics, Iran University of Medical Sciences, Tehran, Iran; 836 Institute for Population Health, King’s College London, London, UK; 837 National Institute of Infectious Diseases, Tokyo, Japan; 838 College of Medicine, Yonsei University, Seodaemun-gu, South Korea; 839 Division of Cardiology, Emory University, Atlanta, GA USA; 840 Finnish Institute of Occupational Health, Helsinki, Finland; 841 Cancer Research Institute, Tehran University of Medical Sciences, Tehran, Iran; 842 Cancer Biology Research Center, Tehran University of Medical Sciences, Tehran, Iran; 843 Institute of Medical Epidemiology, Martin Luther University Halle-Wittenberg, Halle, Germany; 844 Department of Health Education & Promotion, Kermanshah University of Medical Sciences, Kermanshah, Iran; 845 School of Health, University of Technology Sydney, Sydney, New South Wales Australia; 846 Department of Psychology, Reykjavik University, Reykjavik, Iceland; 847 Department of Health and Behavior Studies, Columbia University, New York, NY USA; 848 Department of Physical Education, Federal University of Santa Catarina, Florianopolis, Brazil; 849 Department of Law, Economics, Management and Quantitative Methods, University of Sannio, Benevento, Italy; 850 Menzies Institute for Medical Research, University of Tasmania, Hobart, Tasmania Australia; 851 Global Patient Outcome and Real World Evidence, Eli Lilly and Company, Indianapolis, IN USA; 852 Department of Humanities and Social Sciences, Indian Institute of Technology, Roorkee, Roorkee, India; 853 Department of Pulmonary Medicine, Asthma Bhawan, Jaipur, India; 854 Department of Medicine, University of Alabama at Birmingham, Birmingham, AL USA; 855 Medicine Service, US Department of Veterans Affairs, Birmingham, AL USA; 856 Department of Forensic Medicine, Kathmandu University, Dhulikhel, Nepal; 857 Department of Epidemiology, School of Preventive Oncology, Patna, India; 858 Department of Epidemiology, Healis Sekhsaria Institute for Public Health, Mumbai, India; 859 Department of Midwifery, Haramaya University, Harar, Ethiopia; 860 Department of Physiotherapy and Occupational Therapy, Næstved-Slagelse-Ringsted Hospitals, Slagelse, Denmark; 861 Medical Surgical Nursing Department, Urmia University of Medical Science, Urmia, Iran; 862 Emergency Nursing Department, Semnan University of Medical Sciences, Semnan, Iran; 863 Midwifery Department, Hamadan University of Medical Sciences, Hamadan, Iran; 864 Research Center for Environmental Determinants of Health, Academy of Medical Science, Kermanshah, Iran; 865 Hospital Universitario de la Princesa, Autonomous University of Madrid, Madrid, Spain; 866 Centro de Investigación Biomédica en Red Enfermedades Respiratorias (CIBERES), Madrid, Spain; 867 Department of Research Development, Federal Research Institute for Health Organization and Informatics of the Ministry of Health (FRIHOI), Moscow, Russia; 868 Laboratory of Public Health Indicators Analysis and Health Digitalization, Moscow Institute of Physics and Technology, Moscow, Russia; 869 Hull York Medical School, University of Hull, Hull City, UK; 870 Usher Institute of Population Health Sciences and Informatics, University of Edinburgh, Edinburgh, UK; 871 Department of Parasitology and Mycology, Tabriz University of Medical Sciences, Tabriz, Iran; 872 Division of Community Medicine, International Medical University, Kuala Lumpur, Malaysia; 873 Research Management, Policy, Planning and Coordination, Indian Council of Medical Research, New Delhi, India; 874 Clinical Department, Nutrition and Dietetics Department, Federal Research Institute of Nutrition, Biotechnology and Food Safety, Moscow, Russia; 875 Department of Internal Disease, Pirogov Russian National Research Medical University, Moscow, Russia; 876 Department of Nursing, Muhammadiyah University of Surakarta, Surakarta, Indonesia; 877 Department of Public Health, China Medical University, Taichung City, Taiwan; 878 Department of Community Medicine, Ahmadu Bello University, Zaria, Nigeria; 879 Department of Agriculture and Food Systems, University of Melbourne, Melbourne, Victoria Australia; 880 Norwegian Institute of Public Health, Bergen, Norway; 881 Department of Community Health, Muhimbili University of Health and Allied Sciences, Dar Es Salaam, Tanzania; 882 Muhimbili University of Health and Allied Sciences, Dar Es Salaam, Tanzania; 883 Department of Criminology, Law and Society, University of California Irvine, Irvine, CA USA; 884 Department of Medicine, University of Valencia, Valencia, Spain; 885 Carlos III Health Institute, Biomedical Research Networking Center for Mental Health Network (CiberSAM), Madrid, Spain; 886 Cancer Control Center, Osaka International Cancer Institute, Osaka, Japan; 887 Department of Pediatrics, Hawassa University, Hawassa, Ethiopia; 888 International Vaccine Institute, Seoul, South Korea; 889 Research Center for Molecular Medicine, Hamadan University of Medical Sciences, Hamadan, Iran; 890 School of Pharmacy, Mekelle University, Mekelle, Ethiopia; 891 University Institute ‘Egas Moniz’, Monte da Caparica, Portugal; 892 Research Institute for Medicines, University of Lisbon, Lisbon, Portugal; 893 Department of Public Health, Adigrat University, Adigrat, Ethiopia; 894 Pharmacognosy, Mekelle University, Mekelle, Ethiopia; 895 Department of Pediatrics, King Saud University, Riyadh, Saudi Arabia; 896 College of Medicine, Alfaisal University, Riyadh, Saudi Arabia; 897 Department of Anesthesiology, Perioperative, and Pain Medicine, Stanford University, Standford, CA USA; 898 Department of Anesthesiology, King Fahad Medical City, Riyadh, Saudi Arabia; 899 Department of Endocrinology, Christian Medical College and Hospital (CMC), Vellore, India; 900 Biology Department, Moscow State University, Moscow, Russia; 901 HIV/STI Surveillance Research Center, and WHO Collaborating Center for HIV Surveillance, Kerman University of Medical Sciences, Kerman, Iran; 902 Department of Medicine, University of Calgary, Calgary, Alberta Canada; 903 Department of Pathology and Legal Medicine, University of São Paulo, Ribeirão Preto, Brazil; 904 Clinical Epidemiology and Public Health Research Unit, Burlo Garofolo Institute for Maternal and Child Health, Trieste, Italy; 905 Molecular Medicine and Pathology, University of Auckland, Auckland, New Zealand; 906 Clinical Hematology and Toxicology, Military Medical University, Hanoi, Vietnam; 907 Department of Neurology, All India Institute of Medical Sciences, Delhi, India; 908 Department of Pharmacy, Stamford University Bangladesh, Dhaka, Bangladesh; 909 Gomal Center of Biochemistry and Biotechnology, Gomal University, Dera Ismail Khan, Pakistan; 910 TB Culture Laboratory, Mufti Mehmood Memorial Teaching Hospital Dera Ismail Khan, Dera Ismail Khan, Pakistan; 911 Amity Institute of Biotechnology, Amity University Rajasthan, Jaipur, India; 912 Lifestyle Diseases Research Entity, North-West University, Mmabatho, South Africa; 913 Division of Health Sciences, University of Warwick, Coventry, UK; 914 Department of Epidemiology and Biostatistics, Umeå University, Umeå, Sweden; 915 Argentine Society of Medicine, Buenos Aires, Argentina; 916 Velez Sarsfield Hospital, Buenos Aires, Argentina; 917 Central Research Institute of Cytology and Genetics, Federal Research Institute for Health Organization and Informatics of the Ministry of Health (FRIHOI), Moscow, Russia; 918 Christian Medical College and Hospital (CMC), Vellore, India; 919 UKK Institute, Tampere, Finland; 920 Psychosocial Injuries Research Center, Ilam University of Medical Sciences, Ilam, Iran; 921 National AIDS Control Organisation, Ministry of Health, New Delhi, India; 922 Raffles Neuroscience Centre, Raffles Hospital, Singapore, Singapore; 923 Yong Loo Lin School of Medicine, National University of Singapore, Singapore, Singapore; 924 Community & Family Medicine, All India Institute of Medical Sciences, Bathinda, India; 925 Department of Neurology & Stroke Unit, Sant’Anna Hospital, Como, Italy; 926 Occupational Health Unit, Sant’Orsola Malpighi Hospital, Bologna, Italy; 927 Department of Health Care Administration and Economics, National Research University Higher School of Economics, Moscow, Russia; 928 Department of Global Health and Population, Harvard University, Boston, MA USA; 929 School of Medicine, University of Belgrade, Belgrade, Serbia; 930 Department of Pediatric Endocrinology, Mother and Child Healthcare Institute of Serbia ‘Dr Vukan Cupic’, Belgrade, Serbia; 931 Foundation University Medical College, Foundation University, Islamabad, Pakistan; 932 Department of Epidemiology and Biostatistics, Wuhan University, Wuhan, China; 933 Demographic Change and Ageing Research Area, Federal Institute for Population Research, Wiesbaden, Germany; 934 Department of Physical Therapy, Naresuan University, Meung District, Thailand; 935 Department of Psychology and Counselling, University of Melbourne, Melbourne, Victoria Australia; 936 Department of Medicine, University of Melbourne, St Albans, Victoria Australia; 937 Department of Pharmacology and Toxicology, Mekelle University, Mekelle, Ethiopia; 938 Department of Pharmacology, Addis Ababa University, Addis Ababa, Ethiopia; 939 Department of Nursing, Wollo University, Dessie, Ethiopia; 940 Department of Orthopaedics, Wenzhou Medical University, Wenzhou, China; 941 School of Medicine, Nanjing University, Nanjing, China; 942 Medical Physics Department, Ahvaz Jundishapur University of Medical Sciences, Ahvaz, Iran; 943 Clinical Cancer Research Center, Milad General Hospital, Tehran, Iran; 944 Department of Diabetes and Metabolic Diseases, University of Tokyo, Tokyo, Japan; 945 Department of Preventive Medicine, Northwestern University, Chicago, IL USA; 946 School of International Development and Global Studies, University of Ottawa, Ottawa, Ontario Canada; 947 Health Services Management Research Center, Kerman University of Medical Sciences, Kerman, Iran; 948 Department of Health Management, Policy and Economics, Kerman University of Medical Sciences, Kerman, Iran; 949 Wolkite University, Wolkite, Ethiopia; 950 Centre for Suicide Research and Prevention, University of Hong Kong, Hong Kong, China; 951 Department of Social Work and Social Administration, University of Hong Kong, Hong Kong, China; 952 Department of Psychopharmacology, National Center of Neurology and Psychiatry, Tokyo, Japan; 953 Department of Preventive Medicine, Korea University, Seoul, South Korea; 954 Department of Sociology, Yonsei University, Seoul, South Korea; 955 Department of Health Policy & Management, Jackson State University, Jackson, MS USA; 956 School of Medicine, Tsinghua University, Beijing, China; 957 Department of Environmental Health, Mazandaran University of Medical Sciences, Sari, Iran; 958 Environmental Health, Academy of Medical Science, Sari, Iran; 959 Global Health Institute, Wuhan University, Wuhan, China; 960 Social Determinants of Health Research Center, Ardabil University of Medical Science, Ardabil, Iran; 961 Department of Medicine, Monash University, Melbourne, Victoria Australia; 962 Student Research Committee, Babol University of Medical Sciences, Babol, Iran; 963 Department of Community Medicine, Ardabil University of Medical Science, Ardabil, Iran; 964 Psychiatry and Psychology Research Center, Tehran University of Medical Sciences, Tehran, Iran; 965 Maternal and Child Wellbeing Unit, African Population Health Research Centre, Nairobi, Kenya; 966 Public Health Department, Dilla University, Dilla, Ethiopia; 967 School of Public Health, Wuhan University of Science and Technology, Wuhan, China; 968 Hubei Province Key Laboratory of Occupational Hazard Identification and Control, Wuhan University of Science and Technology, Wuhan, China; 969 Department of Preventive Medicine, Wuhan University, Wuhan, China; 970 School of Biology and Pharmaceutical Engineering, Wuhan Polytechnic University, Wuhan, China

Correction to: *Nature Medicine* 10.1038/s41591-020-0807-6, published online 20 April 2020.

In the version of this article initially published, an author’s name was incorrect (Yesdhambel T. Nigatu). The correct name is ‘Yeshambel T. Nigatu’. Another author’s name and affiliation were incorrect (Mowafa Housseh; Division of Information and Computing Technology, Hamad Bin Khalifa University, Doha, Qatar). The correct name and affiliation are ‘Mowafa Househ’ and ‘College of Science and Engineering, Hamad Bin Khalifa University, Doha, Qatar’. Also, author Ewerton Cousin (affiliation, Postgraduate Program in Epidemiology, Federal University of Rio Grande do Sul, Porto Alegre, Brazil) was not listed. This author has now been included. The errors have been corrected in the HTML and PDF versions of the article.

