# Supplementary material for: Mapping local patterns of childhood overweight and wasting in low- and middle-income countries between 2000 and 2017
Source: Nat Med. 2020 Apr 20;26(5):750–9. doi: 10.1038/s41591-020-0807-6 (PMC7220891; doi:10.1038/s41591-020-0807-6)
Supplement: Supplementary file 1 — Supplementary Tables 1–18; full list of author contributions [file 41591_2020_807_MOESM1_ESM.pdf]

nature medicine

SUPPLEMENTARY INFORMATION

https://doi.org/10.1038/s41591-020-0807-6

In the format provided by the authors and unedited.

OPEN

Mapping local patterns of childhood overweight and wasting in low- and middle-income countries between 2000 and 2017

LBD Double Burden of Malnutrition Collaborators*

*A list of authors and their affiliations appears online. ✉e-mail: sihay@uw.edu

Mapping local patterns of childhood overweight and wasting in low- and middle-income countries between 2000 and 2017

Supplementary Information

Table of Contents

List of Supplementary Tables 2

1.0 Guidelines for Accurate and Transparent Health Estimates Reporting (GATHER) Compliance 3

2.0 Data 4

2.1 DBM Indicator Definitions, Sociodemographic Index (SDI) Classification 4
2.2 Data Sources 8
2.3 Covariates 33

3.0 Model Results 39

4.0 Model Validation 47

4.1 Overweight validation metrics 47
4.2 Wasting validation metrics 48

5.0 Supplementary References 49

6.0 Author Contributions 49

List of Supplementary Tables

Supplementary Table 1: Guidelines for Accurate and Transparent Health Estimates Reporting (GATHER) checklist[1]. 3
Supplementary Table 2: Countries included in model. 5
Supplementary Table 3: Household surveys used in mapping. 8
Supplementary Table 4: Survey Reports added to model. 26
Supplementary Table 5: Data excluded from model. 29
Supplementary Table 6: Covariates used in mapping. 33
Supplementary Table 7: Model prior sensitivity analyses 37
Supplementary Table 8: Spatial hyperparameter priors by region. 38
Supplementary Table 9: Overweight fitted parameters. 39
Supplementary Table 10: Wasting fitted parameters. 40
Supplementary Table 11: Change of overweight and wasting from 2000 to 2017 at the first administrative level in all LMICs. 42
Supplementary Table 12: Change of overweight and wasting from 2000 to 2017 at the second administrative level in all LMICs. 44
Supplementary Table 13: Predictive metrics for overweight aggregated to admin 0. 47
Supplementary Table 14: Predictive metrics for overweight aggregated to admin 1. 47
Supplementary Table 15: Predictive metrics for overweight aggregated to admin 2. 47
Supplementary Table 16: Predictive metrics for wasting aggregated to admin 0. 48
Supplementary Table 17: Predictive metrics for wasting aggregated to admin 1. 48
Supplementary Table 18: Predictive metrics for wasting aggregated to admin 2. 48

1.0 Guidelines for Accurate and Transparent Health Estimates Reporting (GATHER) Compliance

Supplementary Table 1: Guidelines for Accurate and Transparent Health Estimates Reporting (GATHER) checklist[1].

Item #	Checklist item	Reported
**Objectives and funding**		
**1**	Define the indicator(s), populations (including age, sex, and geographic entities), and time period(s) for which estimates were made.	Main text: Introduction, Methods (Data)
**2**	List the funding sources for the work.	Main text: Acknowledgements
**Data Inputs**		
For all data inputs from multiple sources that are synthesized as part of the study:		
**3**	Describe how the data were identified and how the data were accessed.	Main text: Methods (Data: Surveys and child anthropometry data), Supplementary Information: 2.0 Data
**4**	Specify the inclusion and exclusion criteria. Identify all ad-hoc exclusions.	Main text: Methods (Overview; Data: Surveys and child anthropometry data), Supplementary Information: 2.2 Data Sources; Supplementary Table 5
**5**	Provide information on all included data sources and their main characteristics. For each data source used, report reference information or contact name/institution, population represented, data collection method, year(s) of data collection, sex and age range, diagnostic criteria or measurement method, and sample size, as relevant.	Supplementary Information: 2.0 Data
**6**	Identify and describe any categories of input data that have potentially important biases (e.g., based on characteristics listed in item 5).	Main text: Limitations; Supplementary Table 5
For data inputs that contribute to the analysis but were not synthesized as part of the study:		
**7**	Describe and give sources for any other data inputs.	Main text: Methods (Data: Spatial covariates)
For all data inputs:		
**8**	Provide all data inputs in a file format from which data can be efficiently extracted (e.g., a spreadsheet rather than a PDF), including all relevant meta-data listed in item 5. For any data inputs that cannot be shared because of ethical or legal reasons, such as third-party ownership, provide a contact name or the name of the institution that retains the right to the data.	Available at http://ghdx.healthdata.org/record/africa-child-growth-failure-geospatial-estimates–2000–2015 Supplementary Information: 2.0 Data

**Data analysis**		
**9**	Provide a conceptual overview of the data analysis method. A diagram may be helpful.	Main text: Methods (citation to previously published diagram included).
**10**	Provide a detailed description of all steps of the analysis, including mathematical formulae. This description should cover, as relevant, data cleaning, data pre-processing, data adjustments and weighting of data sources, and mathematical or statistical model(s).	Main text: Methods
**11**	Describe how candidate models were evaluated and how the final model(s) were selected.	Main text: Methods
**12**	Provide the results of an evaluation of model performance, if done, as well as the results of any relevant sensitivity analysis.	Main text: Methods (Analysis: Model validation); Supplementary Information: 4.0 Model Validation
**13**	Describe methods for calculating uncertainty of the estimates. State which sources of uncertainty were, and were not, accounted for in the uncertainty analysis.	Main text: Methods (Analysis: Geostatistical model)
**14**	State how analytic or statistical source code used to generate estimates can be accessed.	Available at http://ghdx.healthdata.org/record/africa-child-growth-failure-geospatial-estimates–2000–2015
**Results and Discussion**		
**15**	Provide published estimates in a file format from which data can be efficiently extracted.	Raster files for spatial data and CSVs of estimates available at http://ghdx.healthdata.org/record/africa-child-growth-failure-geospatial-estimates–2000–2015
**16**	Report a quantitative measure of the uncertainty of the estimates (e.g. uncertainty intervals).	Main text: Methods; Figs 1d, 2d; Extended Data Fig 1d
**17**	Interpret results in light of existing evidence. If updating a previous set of estimates, describe the reasons for changes in estimates.	Main text: Global and local variation in malnutrition trends; Prospects for reaching 2025 targets
**18**	Discuss limitations of the estimates. Include a discussion of any modelling assumptions or data limitations that affect interpretation of the estimates.	Main text: Discussion

2.0 Data

2.1 DBM Indicator Definitions, Sociodemographic Index (SDI) Classification

We modelled the prevalence of double burden of malnutrition (DBM) indicators (overweight and wasting) in 105 low- and middle-income countries (LMICs) from 2000 to 2017. These countries were determined by their Sociodemographic Index (SDI), a summary measure of development that combines education, fertility, and poverty, as described by the Global Burden of Disease

(GBD) study[2]. Countries were assigned stages based on their SDI quintile, as well as their geographic continuity. The 105 LMICs (low and middle income countries) modelled in this study are described below, along with their SDI and SDI quintile (e.g., low, low-middle, middle) (Supplementary Table 2). French Guiana and Western Sahara were not modelled by GBD and therefore do not have a calculated SDI, but were included in this study for geographic continuity. China, Iran, Libya, and Malaysia were included despite high-middle SDI status for geographic continuity. Albania and Moldova were excluded despite their middle SDI status due to geographic discontinuity with other included countries and lack of available survey data. We did not estimate for the island nations of American Samoa, Federated States of Micronesia, Fiji, Kiribati, Marshall Islands, North Korea, Samoa, Solomon Islands, or Tonga, where no survey data could be sourced.

Supplementary Table 2: Countries included in model.

Country	Stage	SDI	SDI Quintile
Afghanistan	2	0.2903	Low SDI
Algeria	1	0.6958	Middle SDI
Angola	1	0.4605	Low-middle SDI
Bangladesh	2	0.4580	Low SDI
Belize	2	0.6022	Low-middle SDI
Benin	1	0.3734	Low SDI
Bhutan	2	0.5699	Low-middle SDI
Bolivia	2	0.5874	Low-middle SDI
Botswana	1	0.6632	Middle SDI
Brazil	2	0.6633	Middle SDI
Burkina Faso	1	0.2839	Low SDI
Burundi	1	0.3097	Low SDI
Cambodia	2	0.4816	Low-middle SDI
Cameroon	1	0.4820	Low-middle SDI
Cape Verde	1	0.5491	Low-middle SDI
Central African Republic	1	0.3344	Low SDI
Chad	1	0.2529	Low SDI
China	2	0.7073	High-middle SDI
Colombia	2	0.6337	Middle SDI
Comoros	1	0.4343	Low SDI
Costa Rica	2	0.6621	Middle SDI
Côte d'Ivoire	1	0.4121	Low SDI
Cuba	2	0.6877	Middle SDI
Democratic Republic of the Congo	1	0.3645	Low SDI
Djibouti	1	0.4848	Low-middle SDI
Dominican Republic	2	0.5926	Low-middle SDI
Ecuador	2	0.6356	Middle SDI
Egypt	1	0.6043	Low-middle SDI

Country	Stage	SDI	SDI Quintile
El Salvador	2	0.5931	Low-middle SDI
Equatorial Guinea	1	0.6252	Middle SDI
Eritrea	1	0.4088	Low SDI
Ethiopia	1	0.3342	Low SDI
French Guiana	2	NA	NA
Gabon	1	0.6506	Middle SDI
Ghana	1	0.5370	Low-middle SDI
Guatemala	2	0.5242	Low-middle SDI
Guinea	1	0.3247	Low SDI
Guinea-Bissau	1	0.3490	Low SDI
Guyana	2	0.5837	Low-middle SDI
Haiti	2	0.4417	Low SDI
Honduras	2	0.5123	Low-middle SDI
India	2	0.5502	Low-middle SDI
Indonesia	2	0.6476	Middle SDI
Iran	2	0.7001	High-middle SDI
Iraq	2	0.5848	Low-middle SDI
Jamaica	2	0.6785	Middle SDI
Jordan	2	0.6968	Middle SDI
Kenya	1	0.4995	Low-middle SDI
Kyrgyzstan	2	0.6066	Low-middle SDI
Laos	2	0.5188	Low-middle SDI
Lesotho	1	0.4934	Low-middle SDI
Liberia	1	0.3284	Low SDI
Libya	1	0.7609	High-middle SDI
Madagascar	1	0.3308	Low SDI
Malawi	1	0.3493	Low SDI
Malaysia	2	0.7592	High-middle SDI
Mali	1	0.2669	Low SDI
Mauritania	1	0.4706	Low-middle SDI
Mexico	2	0.6284	Middle SDI
Mongolia	2	0.6619	Middle SDI
Morocco	1	0.5792	Low-middle SDI
Mozambique	1	0.3405	Low SDI
Myanmar	2	0.5558	Low-middle SDI
Namibia	1	0.6158	Middle SDI
Nepal	2	0.4285	Low SDI
Nicaragua	2	0.5296	Low-middle SDI
Niger	1	0.1906	Low SDI
Nigeria	1	0.4934	Low-middle SDI
Pakistan	2	0.4922	Low-middle SDI
Palestine	2	0.5414	Low-middle SDI

Country	Stage	SDI	SDI Quintile
Panama	2	0.6770	Middle SDI
Papua New Guinea	2	0.4190	Low SDI
Paraguay	2	0.6188	Middle SDI
Peru	2	0.6358	Middle SDI
Philippines	2	0.6172	Middle SDI
Republic of the Congo	1	0.5741	Low-middle SDI
Rwanda	1	0.4074	Low SDI
São Tomé and Príncipe	1	0.4883	Low-middle SDI
Senegal	1	0.3730	Low SDI
Sierra Leone	1	0.3572	Low SDI
Somalia	1	0.2348	Low SDI
South Africa	1	0.6765	Middle SDI
South Sudan	1	0.2747	Low SDI
Sri Lanka	2	0.6797	Middle SDI
Sudan	1	0.4779	Low-middle SDI
Suriname	2	0.6410	Middle SDI
Swaziland	1	0.5777	Low-middle SDI
Syria	2	0.6111	Middle SDI
Tajikistan	2	0.5226	Low-middle SDI
Tanzania	1	0.4122	Low SDI
Thailand	2	0.6843	Middle SDI
The Gambia	1	0.4048	Low SDI
Timor-Leste	2	0.5048	Low-middle SDI
Togo	1	0.4133	Low SDI
Trinidad and Tobago	2	0.6984	Middle SDI
Tunisia	1	0.6754	Middle SDI
Turkmenistan	2	0.6964	Middle SDI
Uganda	1	0.3877	Low SDI
Uzbekistan	2	0.6295	Middle SDI
Venezuela	2	0.6554	Middle SDI
Vietnam	2	0.6068	Middle SDI
Western Sahara	1	NA	NA
Yemen	2	0.4295	Low SDI
Zambia	1	0.4722	Low-middle SDI
Zimbabwe	1	0.4632	Low-middle SDI

2.2 Data Sources

The data sources used to model overweight and wasting indicators are described below. Information on survey locations, years, source, and number of individuals, polygons, and/or geo-positioned clusters can be found in Supplementary Tables 3 and 4. Excluded datasets and reasons for their exclusion from our analysis are detailed in Supplementary Table 5.

Supplementary Table 3: Household surveys used in mapping.

Number identification (NID) can be used to locate a particular data source in the Global Health Data Exchange (GHDx) at http://ghdx.healthdata.org/.

†Data source is not publicly available due to restrictions by the data provider and was used under license for the current study.

All data sources included in this study are cited unless prohibited by original data holder.

Country	Survey year(s)	Survey Name	Number of Individuals	Number of geo-positioned clusters	Number of polygons (areal)	GHDx NID
Algeria	2002–2003	Algeria Family Health Survey 2002–2003	4754	0	47	627†
Algeria	2012–2013	Algeria Multiple Indicator Cluster Survey 2012–2013	13909	0	7	210614
Angola	2001	Angola Multiple Indicator Cluster Survey 2001	5461	0	18	687
Angola	2015–2016	Angola Demographic and Health Survey 2015–2016	6583	625	0	218555
Bangladesh	1999–2000	Bangladesh Demographic and Health Survey 1999–2000	5970	341	0	26826
Bangladesh	2004	Bangladesh Demographic and Health Survey 2004	6186	359	0	18902
Bangladesh	2007	Bangladesh Demographic and Health Survey 2007	5531	361	0	18913
Bangladesh	2011–2012	Bangladesh Demographic and Health Survey 2011–2012	7992	600	0	55956
Bangladesh	2012–2013	Bangladesh Multiple Indicator Cluster Survey 2012–2013	19397	2625	0	151086
Bangladesh	2014	Bangladesh Demographic and Health Survey 2014	7341	0	7	157021
Belize	2006	Belize Multiple Indicator Cluster Survey 2006	770	0	6	1089
Belize	2011	Belize Multiple Indicator Cluster Survey 2011	1831	0	7	76699
Belize	2015–2016	Belize Multiple Indicator Cluster Survey 2015–2016	2448	0	6	264910
Benin	2001	Benin Demographic and Health Survey 2001	4533	247	0	18950
Benin	2006	Benin Demographic and Health Survey 2006	13517	0	12	18959

Country	Survey year(s)	Survey Name	Number of Individuals	Number of geo-positioned clusters	Number of polygons (areal)	GHDx NID
Benin	2014	Benin Multiple Indicator Cluster Survey 2014	12113	0	12	206075
Benin	2017–2018	Benin Demographic and Health Survey 2017–2018	11832	540	0	218565
Bhutan	2010	Bhutan Multiple Indicator Cluster Survey 2010	6171	0	20	40028
Bolivia	2003–2004	Bolivia Demographic and Health Survey 2003–2004	9396	0	8	19001
Bolivia	2008	Bolivia Demographic and Health Survey 2008	7880	986	0	19016
Bolivia	2012	Bolivia Health and Nutrition Assessment Survey 2012	10941	7320	0	285880
Botswana	2000	Botswana Multiple Indicator Cluster Survey 2000	2877	0	14	1404
Botswana	2007–2008	Botswana Family Health Survey 2007–2008	2167	0	323	22125†
Brazil	2002–2003	Brazil Consumer Expenditure Survey 2002–2003	17411	0	27	33019
Brazil	2006–2007	Brazil National Demographic and Health Survey of Children and Women 2006–2007	4549	0	5	141948
Burkina Faso	2003	Burkina Faso Core Welfare Indicators Questionnaire Survey 2003	7797	0	232	1855†
Burkina Faso	2003	Burkina Faso Demographic and Health Survey 2003	8808	397	0	19088
Burkina Faso	2006	Burkina Faso Multiple Indicator Cluster Survey 2006	5101	195	0	1927
Burkina Faso	2007	Burkina Faso Core Welfare Indicators Questionnaire Survey 2007	4417	0	13	18499†
Burkina Faso	2010–2011	Burkina Faso Demographic and Health Survey 2010–2011	6381	540	0	19133
Burkina Faso	2014	Burkina Faso Continuous Multisectoral Survey 2014	11007	0	13	236156
Burundi	2000	Burundi Multiple Indicator Cluster Survey 2000	2689	0	17	1994
Burundi	2010–2011	Burundi Demographic and Health Survey 2010–2011	3520	376	0	30431
Burundi	2016–2017	Burundi Demographic and Health Survey 2016–2017	6059	552	0	286766
Cambodia	2000	Cambodia Demographic and Health Survey 2000	3785	467	0	19156
Cambodia	2003–2005	Cambodia Socio-Economic Survey 2003–2005	2656	0	14	30963†
Cambodia	2005–2006	Cambodia Demographic and Health Survey 2005–2006	3673	546	0	19167
Cambodia	2006–2007	Cambodia Socio-Economic Survey 2006–2007	1593	27	260	31050†
Cambodia	2008	Cambodia Anthropometric Survey 2008 - National Institute of Statistics	8580	0	709	135773†
Cambodia	2009	Cambodia Socio-Economic Survey 2009	5456	0	517	31143†

Country	Survey year(s)	Survey Name	Number of Individuals	Number of geo-positioned clusters	Number of polygons (areal)	GHDx NID
Cambodia	2010–2011	Cambodia Demographic and Health Survey 2010–2011	3816	604	0	30379
Cambodia	2014	Cambodia Demographic and Health Survey 2014	4466	608	0	157024
Cameroon	2004	Cameroon Demographic and Health Survey 2004	3352	446	0	19211
Cameroon	2006	Cameroon Multiple Indicator Cluster Survey 2006	6178	0	191	2063
Cameroon	2011	Cameroon Demographic and Health Survey 2011	5211	574	0	19274
Cameroon	2014	Cameroon Multiple Indicator Cluster Survey 2014	6758	0	206	244455
Central African Republic	2000	Central African Republic Multiple Indicator Cluster Survey 2000	13722	0	17	2209
Central African Republic	2006	Central African Republic Multiple Indicator Cluster Survey 2006	9240	0	16	2223
Central African Republic	2010–2011	Central African Republic Multiple Indicator Cluster Survey 2010–2011	10344	0	17	82832
Chad	2000	Chad Multiple Indicator Cluster Survey 2000	5314	0	15	2244
Chad	2004	Chad Demographic and Health Survey 2004	4725	0	9	19315
Chad	2010	Chad Multiple Indicator Cluster Survey 2010	15641	0	60	76701
Chad	2014–2015	Chad Demographic and Health Survey 2014–2015	10524	623	0	157025
China	1989–2011	China Health and Nutrition Survey 1989–2011	5207	0	9	200838
China	2016	China Family Dynamics Survey 2016	1577	0	301	399041†
Colombia	2000	Colombia Demographic and Health Survey 2000	4264	0	23	19359
Colombia	2004–2005	Colombia Demographic and Health Survey 2004–2005	12574	0	33	19324
Colombia	2009–2010	Colombia Demographic and Health Survey 2009–2010	15798	4279	0	21281
Comoros	2000	Comoros Multiple Indicator Cluster Survey 2000	4623	0	3	3114
Comoros	2012–2013	Comoros Demographic and Health Survey 2012–2013	2657	241	0	76850
Côte d'Ivoire	2006	Côte d'Ivoire Multiple Indicator Cluster Survey 2006	8621	0	52	26433
Côte d'Ivoire	2011–2012	Côte d'Ivoire Demographic and Health Survey 2011–2012	3228	341	0	18533

Country	Survey year(s)	Survey Name	Number of Individuals	Number of geo-positioned clusters	Number of polygons (areal)	GHDx NID
Côte d'Ivoire	2016	Cote d'Ivoire Multiple Indicator Cluster Survey 2016	9032	0	11	218611
Cuba	2014	Cuba Multiple Indicator Cluster Survey 2014	5397	0	4	169975
Democratic Republic of the Congo	2001	Democratic Republic of the Congo Multiple Indicator Cluster Survey 2001	10073	0	11	3161
Democratic Republic of the Congo	2007	Democratic Republic of the Congo Demographic and Health Survey 2007	3647	293	0	19381
Democratic Republic of the Congo	2010	Democratic Republic of the Congo Multiple Indicator Cluster Survey 2010	10233	357	0	26998
Democratic Republic of the Congo	2013	Democratic Republic of the Congo Demographic and Health Survey 2013–2014	7765	492	0	76878
Djibouti	2006	Djibouti Multiple Indicator Cluster Survey 2006	2193	35	1	3404
Djibouti	2012	Djibouti Family Health Survey 2012	3549	0	6	218035
Dominican Republic	2000	Dominican Republic Multiple Indicator Cluster Survey 2000	1918	0	30	27069
Dominican Republic	2002	Dominican Republic Demographic and Health Survey 2002	9613	0	32	19444
Dominican Republic	2006	Dominican Republic National Multipurpose Household Survey 2006	3788	0	32	3455†
Dominican Republic	2007	Dominican Republic Demographic and Health Survey 2007	9559	1397	0	19456
Dominican Republic	2007	Dominican Republic Special Demographic and Health Survey 2007	811	0	9	21198
Dominican Republic	2013	Dominican Republic Demographic and Health Survey 2013	3251	513	0	77819

Country	Survey year(s)	Survey Name	Number of Individuals	Number of geo-positioned clusters	Number of polygons (areal)	GHDx NID
Dominican Republic	2013	Dominican Republic Special Demographic and Health Survey 2013	794	111	0	165645
Ecuador	2004	Ecuador Reproductive Health Survey 2004	5297	680	0	27630
Ecuador	2005–2006	Ecuador Living Conditions Survey 2005–2006	5614	283	88	46924
Ecuador	2012	Ecuador National Health and Nutrition Survey 2012	6151	0	480	153674
Egypt	2000	Egypt Demographic and Health Survey 2000	10743	985	0	19511
Egypt	2003	Egypt Interim Demographic and Health Survey 2003	5425	876	0	19529
Egypt	2005	Egypt Demographic and Health Survey 2005	12639	1288	0	19521
Egypt	2008	Egypt Demographic and Health Survey 2008	10331	1221	0	26842
Egypt	2014	Egypt Demographic and Health Survey 2014	15153	1736	0	154897
Egypt	2013–2014	Egypt IPHN Rural Districts Multiple Indicator Cluster Survey 2013–2014	5090	0	6	159617
El Salvador	2002–2003	El Salvador Reproductive Health Survey 2002–2003	5328	0	14	27599
El Salvador	2008	El Salvador Reproductive Health Survey 2008	4651	0	14	27606
El Salvador	2014	El Salvador Multiple Indicator Cluster Survey 2014	7288	0	14	200636
Equatorial Guinea	2000	Equatorial Guinea Multiple Indicator Cluster Survey 2000	2424	0	7	3655
Eritrea	2002	Eritrea Demographic and Health Survey 2002	5727	0	6	19539†
Ethiopia	2017	Spatial heterogeneity and risk factors for stunting among children under age five in Ethiopia: A Bayesian geo-statistical model	3974	3035	0	319322†
Ethiopia	2000	Ethiopia Demographic and Health Survey 2000	9062	533	0	19571
Ethiopia	2005	Ethiopia Demographic and Health Survey 2005	4196	520	0	19557
Ethiopia	2010–2011	Ethiopia Demographic and Health Survey 2010–2011	9639	571	0	21301
Ethiopia	2011–2012	Ethiopia Rural Socioeconomic Survey 2011–2012	2474	332	0	93848
Ethiopia	2014	Ethiopia Mini Demographic and Health Survey 2014	4888	0	11	153507†
Ethiopia	2013–2014	Ethiopia Socioeconomic Survey 2013–2014	2792	434	0	235215
Ethiopia	2016	Ethiopia Demographic and Health Survey 2016	8779	619	0	218568

Country	Survey year(s)	Survey Name	Number of Individuals	Number of geo-positioned clusters	Number of polygons (areal)	GHDx NID
Ethiopia	2015–2016	Ethiopia Socioeconomic Survey 2015–2016	2749	462	0	286657
Ethiopia	2015–2016	Ethiopia Welfare Monitoring Survey 2015–2016	10292	7463	2	365281†
Gabon	2000–2001	Gabon Demographic and Health Survey 2000–2001	3585	0	40	19579
Gabon	2012	Gabon Demographic and Health Survey 2012	3500	324	0	76706
Ghana	2003	Ghana Demographic and Health Survey 2003	3270	407	0	19627
Ghana	2003	Ghana Core Welfare Indicators Questionnaire Survey 2003	24011	0	110	23017†
Ghana	2006	Ghana Multiple Indicator Cluster Survey 2006	3413	0	10	4694
Ghana	2008	Ghana Demographic and Health Survey 2008	2529	400	0	21188
Ghana	2007–2008	Ghana District Multiple Indicator Cluster Survey 2007–2008	8415	0	4	160576†
Ghana	2010–2011	Ghana - Accra Multiple Indicator Cluster Survey 2010–2011	439	5	0	56241
Ghana	2011	Ghana Multiple Indicator Cluster Survey 2011	6902	738	0	63993
Ghana	2014	Ghana Demographic and Health Survey 2014	2721	414	0	157027
Guatemala	2000	Guatemala Living Standards Measurement Survey 2000	5708	0	8	45718
Guatemala	2002	Guatemala Reproductive Health Survey 2002	6538	370	373	27563
Guatemala	2008–2009	Guatemala Reproductive Health Survey 2008–2009	8299	0	22	4779
Guatemala	2014–2015	Guatemala Demographic and Health Survey 2014–2015	11761	851	0	157031
Guinea	2005	Guinea Demographic and Health Survey 2005	2718	290	0	19683
Guinea	2012	Guinea Demographic and Health Survey 2012	3232	300	0	69761
Guinea	2016	Guinea Multiple Indicator Cluster Survey 2016	6756	0	8	303458
Guinea-Bissau	2000	Guinea-Bissau Multiple Indicator Cluster Survey 2000	5728	0	9	4808
Guinea-Bissau	2006	Guinea-Bissau Multiple Indicator Cluster Survey 2006	5670	0	9	4818
Guinea-Bissau	2014	Guinea-Bissau Multiple Indicator Cluster Survey 2014	7579	0	9	174049
Guyana	2000	Guyana Multiple Indicator Cluster Survey 2000	2581	0	10	4916
Guyana	2006–2007	Guyana Multiple Indicator Cluster Survey 2006–2007	2381	0	10	4926
Guyana	2009	Guyana Demographic and Health Survey 2009	1676	307	0	21348
Guyana	2014	Guyana Multiple Indicator Cluster Survey 2014	3110	250	0	200598
Haiti	2000	Haiti Demographic and Health Survey 2000	5660	317	0	19708

Country	Survey year(s)	Survey Name	Number of Individuals	Number of geo-positioned clusters	Number of polygons (areal)	GHDx NID
Haiti	2005–2006	Haiti Demographic and Health Survey 2005–2006	2529	331	0	19720
Haiti	2012	Haiti Demographic and Health Survey 2012	4002	435	0	65118
Haiti	2016–2017	Haiti Demographic and Health Survey 2016–2017	5654	449	10	218574
Honduras	2001	Honduras Reproductive Health Survey 2001	5690	0	16	27551
Honduras	2004	Honduras Survey of Living Conditions 2004	4834	0	18	5009†
Honduras	2005–2006	Honduras Demographic and Health Survey 2005–2006	9427	0	16	19728
Honduras	2011–2012	Honduras Demographic and Health Survey 2011–2012	9920	1119	0	95440
India	1998–2000	India Demographic and Health Survey 1998–1999	27937	0	438	19950†
India	2000–2001	India Rural Survey of Diet and Nutritional Status 2000–2001	9262	0	9	129913†
India	2004–2005	India Human Development Survey 2004–2005	14265	0	363	26919
India	2005–2006	India Demographic and Health Survey 2005–2006	44061	0	29	19963
India	2004–2006	India Rural Survey of Diet and Nutritional Status 2004–2006	6670	0	9	129905†
India	2007–2011	India - Kolkata Global Enteric Multicenter Study 2007–2011	2014	1	0	224240†
India	2011–2012	India Rural Third Repeat Survey of Diet and Nutritional Status 2011–2012	10300	1125	3	129770†
India	2011–2013	India - Kolkata Global Enteric Multicenter Study 2011–2013	1172	1	0	224854†
India	2012–2014	India District Level Household Survey 2012–2014	73542	0	271	165390
India	2014	India Clinical, Anthropometric and Bio-chemical Survey 2014	108693	0	277	233917
India	2015–2016	India Demographic and Health Survey 2015–2016	237528	28118	0	157050
India	2015–2016	India Urban Nutrition Survey 2015–2016	12027	564	0	334953†
Indonesia	2000	Indonesia Family Life Survey 2000	3984	0	16	6111
Indonesia	2007	Indonesia Family Life Survey 2007–2008	4776	0	15	6464
Indonesia	2012	Indonesia Family Life Survey East 2012	1288	0	7	219201
Indonesia	2014–2015	Indonesia Family Life Survey 2014–2015	5354	0	1149	264956
Iran	2004	Iran Anthropometric Nutritional Indicators Survey 2004	29434	0	265	159873†
Iraq	2000	Iraq Multiple Indicator Cluster Survey 2000	14378	0	18	7054
Iraq	2004	Iraq Multiple Indicator Rapid Assessment 2004	17585	2027	0	23565†

Country	Survey year(s)	Survey Name	Number of Individuals	Number of geo-positioned clusters	Number of polygons (areal)	GHDx NID
Iraq	2006	Iraq Multiple Indicator Cluster Survey 2006	16473	0	18	7028
Iraq	2011	Iraq Multiple Indicator Cluster Survey 2011	35847	0	48	76707
Iraq	2012–2013	Iraq Household Socioeconomic Survey 2012–2013	25777	14018	18	235348
Iraq	2018	Iraq Multiple Indicator Cluster Survey 2018	16582	0	18	385708
Jamaica	2000	Jamaica Survey of Living Conditions 2000	562	0	14	45856†
Jamaica	2001	Jamaica Survey of Living Conditions 2001	452	0	14	7222†
Jamaica	2002	Jamaica Survey of Living Conditions 2002	1901	0	14	80626†
Jamaica	2004	Jamaica Survey of Living Conditions 2004	571	0	14	141572†
Jordan	2002	Jordan Demographic and Health Survey 2002	5014	492	0	20073
Jordan	2007	Jordan Demographic and Health Survey 2007	4803	462	0	20083
Jordan	2009	Jordan Interim Demographic and Health Survey 2009	4470	0	12	21206
Jordan	2012	Jordan Demographic and Health Survey 2012	6410	798	0	77517
Kenya	2000	Kenya Multiple Indicator Cluster Survey 2000	6709	802	0	7387
Kenya	2003	Kenya Demographic and Health Survey 2003	4957	397	0	20145
Kenya	2005–2006	Kenya Integrated Household Budget Survey 2005–2006	7096	1284	0	7375†
Kenya	2007	Kenya - North Eastern Province Multiple Indicator Cluster Survey 2007	920	76	0	155335
Kenya	2008	Kenya - Eastern Province Multiple Indicator Cluster Survey 2008	12300	590	0	7401
Kenya	2008–2009	Kenya Demographic and Health Survey 2008–2009	5373	397	0	21365
Kenya	2009	Kenya - Coast Multiple Indicator Cluster Survey 2009	446	0	1	56420
Kenya	2011	Kenya - Nyanza Province Multiple Indicator Cluster Survey 2011	4844	289	0	135416
Kenya	2014	Kenya Demographic and Health Survey 2014	18967	1583	0	157057
Kenya	2013–2014	Kenya - Bungoma County Multiple Indicator Survey 2013–2014	812	40	0	203654†
Kenya	2013–2014	Kenya - Kakamega County Multiple Indicator Survey 2013–2014	738	48	0	203663†
Kenya	2013–2014	Kenya - Turkana County Multiple Indicator Survey 2013–2014	1032	50	0	203664†
Kyrgyzstan	2005–2006	Kyrgyzstan Multiple Indicator Cluster Survey 2005–2006	2855	185	2	7540
Kyrgyzstan	2012	Kyrgyzstan Demographic and Health Survey 2012	4067	313	0	77518

Country	Survey year(s)	Survey Name	Number of Individuals	Number of geo-positioned clusters	Number of polygons (areal)	GHDx NID
Kyrgyzstan	2014	Kyrgyzstan Multiple Indicator Cluster Survey 2014	4364	243	2	162283
Kyrgyzstan	2018	Kyrgyzstan Multiple Indicator Cluster Survey 2018	3480	0	5	408226
Laos	2000	Laos Multiple Indicator Cluster Survey 2000	1554	86	0	7618
Laos	2006	Laos Multiple Indicator Cluster Survey 2006	4050	300	0	7629
Laos	2011–2012	Laos Multiple Indicator Cluster Survey 2011–2012	10887	0	17	103973
Laos	2017	Laos Multiple Indicator Cluster Survey 2017	11603	0	18	375362
Lesotho	2004–2005	Lesotho Demographic and Health Survey 2004–2005	1399	353	0	20167
Lesotho	2009–2010	Lesotho Demographic and Health Survey 2009–2010	1664	383	0	21382
Lesotho	2014	Lesotho Demographic and Health Survey 2014	1360	369	0	157058
Liberia	2006–2007	Liberia Demographic and Health Survey 2006–2007	4550	290	0	20191
Liberia	2013	Liberia Demographic and Health Survey 2013	3290	322	0	77385
Madagascar	2003–2004	Madagascar Demographic and Health Survey 2003–2004	4765	0	6	20223
Malawi	2000	Malawi Demographic and Health Survey 2000	9883	559	0	20252
Malawi	2004–2005	Malawi Demographic and Health Survey 2004–2005	8971	520	0	20263
Malawi	2004–2005	Malawi Living Standards Measurement Survey 2004–2005	6801	0	26	46317
Malawi	2006	Malawi Multiple Indicator Cluster Survey 2006	22573	0	26	7919
Malawi	2010	Malawi Demographic and Health Survey 2010	4838	813	0	21393
Malawi	2010–2011	Malawi Integrated Household Survey 2010–2011	7750	768	0	93806
Malawi	2010–2011	Malawi Integrated Household Panel Survey, Short-Term Panel, 2010–2013	4683	549	0	336401
Malawi	2013	Malawi Integrated Household Survey 2013	2503	547	0	224223†
Malawi	2013	Malawi Integrated Household Panel Survey, Short-Term Panel, 2010–2013	4683	549	0	336401
Malawi	2013–2014	Malawi Multiple Indicator Cluster Survey 2013–2014	18673	0	31	161662
Malawi	2015–2016	Malawi Demographic and Health Survey 2015–2016	5284	850	0	218581
Malawi	2016–2017	Malawi Integrated Household Survey 2016–2017	6306	0	32	327852

Country	Survey year(s)	Survey Name	Number of Individuals	Number of geo-positioned clusters	Number of polygons (areal)	GHDx NID
Malawi	2016–2017	Malawi Integrated Household Panel Survey, Long-Term Panel, 2010–2016	1396	0	28	327857
Mali	2001	Mali Demographic and Health Survey 2001	10043	399	0	20315
Mali	2006	Mali Demographic and Health Survey 2006	11677	405	0	20274
Mali	2009–2010	Mali Multiple Indicator Cluster Survey 2009–2010	23082	0	50	270627
Mali	2012–2013	Mali Demographic and Health Survey 2012–2013	4660	412	0	77388
Mali	2015	Mali Multiple Indicator Cluster Survey 2015	15881	0	8	248224
Mauritania	2000–2001	Mauritania Demographic and Health Survey 2000–2001	4042	0	13	20322
Mauritania	2007	Mauritania Multiple Indicator Cluster Survey 2007	8283	0	196	8115
Mauritania	2011	Mauritania Multiple Indicator Cluster Survey 2011	8977	0	194	152783
Mauritania	2015	Mauritania Multiple Indicator Cluster Survey 2015	10285	0	13	267343
Mexico	2005–2006	Mexico National Survey of Health and Nutrition 2005–2006	8454	0	576	8618
Mexico	2011–2012	Mexico National Survey of Health and Nutrition 2011–2012	8951	0	2	81748
Mexico	2008–2013	Mexico Family Life Survey 2008–2013	3312	0	197	160781
Mexico	2016	Mexico National Survey of Health and Nutrition Mid-way 2016	2082	440	0	316736
Mongolia	2000	Mongolia Multiple Indicator Cluster Survey 2000	5958	0	17	8788
Mongolia	2005	Mongolia Multiple Indicator Cluster Survey 2005	3377	0	22	8777
Mongolia	2012	Mongolia - Khövsgöl Multiple Indicator Cluster Survey 2012	745	0	23	189045
Mongolia	2012	Mongolia - Nalaikh District Multiple Cluster Indicator Survey 2012	427	0	1	189048
Mongolia	2013	Mongolia Multiple Indicator Cluster Survey 2013	5771	0	527	150866
Mongolia	2016	Mongolia - Khövsgöl Multiple Indicator Cluster Survey 2016	1084	0	82	335994
Mongolia	2016	Mongolia - Nalaikh District Multiple Indicator Cluster Survey 2016	368	0	7	336042†
Morocco	2003–2004	Morocco Demographic and Health Survey 2003–2004	5711	479	0	20361
Morocco	2010–2011	Morocco National Survey on Population and Family Health 2010–2011	6420	0	59	126909†
Mozambique	2003	Mozambique Demographic and Health Survey 2003–2004	8367	0	11	20394
Mozambique	2008–2009	Mozambique Multiple Indicator Cluster Survey 2008–2009	10948	67	618	27031

Country	Survey year(s)	Survey Name	Number of Individuals	Number of geo-positioned clusters	Number of polygons (areal)	GHDx NID
Mozambique	2011	Mozambique Demographic and Health Survey 2011	9753	609	0	55975
Myanmar	2000	Myanmar Multiple Indicator Cluster Survey 2000	8592	0	16	8932
Myanmar	2003	Myanmar Multiple Indicator Cluster Survey 2003	5991	0	17	141910†
Myanmar	2009–2010	Myanmar Multiple Indicator Cluster Survey 2009–2010	15545	0	17	90696†
Myanmar	2015–2016	Myanmar Demographic and Health Survey 2015–2016	4320	439	0	157061
Namibia	2000	Namibia Demographic and Health Survey 2000	3075	256	0	20417
Namibia	2006–2007	Namibia Demographic and Health Survey 2006–2007	3809	484	0	20428
Namibia	2009–2010	Namibia Household Income and Expenditure Survey 2009–2010	5696	0	13	134371
Namibia	2013	Namibia Demographic and Health Survey 2013	1882	504	0	150382
Nepal	2001	Nepal Demographic and Health Survey 2001	6295	248	0	20450
Nepal	2006	Nepal Demographic and Health Survey 2006	5319	260	0	20462
Nepal	2011	Nepal Demographic and Health Survey 2011	2365	288	0	21240
Nepal	2014	Nepal Multiple Indicator Cluster Survey 2014	5170	508	0	162317
Nepal	2016	Nepal Household Risk and Vulnerability Survey 2016, Wave 1	1826	0	50	400219
Nepal	2016–2017	Nepal Demographic and Health Survey 2016–2017	2382	375	73	286782
Nicaragua	2001	Nicaragua Living Standards Measurement Survey 2001	2538	0	117	9422
Nicaragua	2001	Nicaragua Demographic and Health Survey 2001	6219	0	133	20487
Nicaragua	2005	Nicaragua Living Standards Measurement Survey 2005	3583	0	134	44645
Nicaragua	2006–2007	Nicaragua Reproductive Health Survey 2006–2007	6286	0	141	9270
Niger	2000	Niger Multiple Indicator Cluster Survey 2000	4929	0	8	9439
Niger	2006	Niger Demographic and Health Survey 2006	3901	0	8	20499
Niger	2012	Niger Demographic and Health Survey 2012	5208	0	8	74393
Nigeria	2003	Nigeria Demographic and Health Survey 2003	4813	359	0	20567
Nigeria	2008	Nigeria Demographic and Health Survey 2008	23492	886	0	21433
Nigeria	2011	Nigeria Multiple Indicator Cluster Survey 2011	24478	0	37	76703
Nigeria	2013	Nigeria Demographic and Health Survey 2013	26945	888	0	77390
Nigeria	2012–2013	Nigeria General Household Survey 2012–2013	2801	487	0	151797

Country	Survey year(s)	Survey Name	Number of Individuals	Number of geo-positioned clusters	Number of polygons (areal)	GHDx NID
Nigeria	2015–2016	Nigeria General Household Survey 2015–2016	2898	512	0	274160
Nigeria	2016–2017	Nigeria Multiple Indicator Cluster Survey with National Immunization Coverage Survey Supplement 2016–2017	26948	2136	0	218613
Pakistan	2011	Pakistan National Nutrition Survey 2011	28603	1001	0	141521†
Pakistan	2012–2013	Pakistan Demographic and Health Survey 2012–2013	3712	0	6	77521
Pakistan	2014	Pakistan - Sindh Multiple Indicator Cluster Survey 2014	16086	0	28	232763
Pakistan	2014	Pakistan - Punjab Multiple Indicator Cluster Survey 2014	26900	0	36	236266
Pakistan	2017–2018	Pakistan Demographic and Health Survey 2017–2018	4271	553	0	286783
Palestine	2000	Palestine - West Bank and Gaza Strip Multiple Indicator Cluster Survey 2000	6029	0	2	10001†
Palestine	2002	Palestine Nutrition Survey 2002	3347	0	2	9989†
Palestine	2004	Palestine Demographic and Health Survey 2004	4691	0	2	20596†
Palestine	2006–2007	Palestine Family Health Survey 2006–2007	9480	0	16	9999†
Palestine	2010	Palestine Multiple Indicator Cluster Survey 2010	9408	0	16	125591
Palestine	2014	Palestine Multiple Indicator Cluster Survey 2014	7256	0	16	161590
Panama	2003	Panama Living Standard Measurement Survey 2003	2931	0	12	10224
Panama	2008	Panama Living Standard Measurement Survey 2008	2519	0	12	46517
Paraguay	2016	Paraguay Multiple Indicator Cluster Survey 2016	4463	0	9	324470
Peru	2000	Peru National Living Standards Measurement Survey 2000	1854	0	225	10460
Peru	2000	Peru Demographic and Health Survey 2000	11897	1383	0	20649
Peru	2003–2008	Peru Continuous Demographic and Health Survey 2003–2008	7022	825	0	275090
Peru	2007–2008	Peru Monitoring of Nutritional Indicators in the National Household Survey 2007–2008	4175	0	604	359163†
Peru	2009	Peru Continuous Demographic and Health Survey 2009	9468	1121	0	270404
Peru	2010	Peru Continuous Demographic and Health Survey 2010	8854	0	24	270469
Peru	2009–2010	Peru Monitoring of Nutritional Indicators in the National Household Survey 2009–2011	6062	0	833	359146†

Country	Survey year(s)	Survey Name	Number of Individuals	Number of geo-positioned clusters	Number of polygons (areal)	GHDx NID
Peru	2011	Peru Continuous Demographic and Health Survey 2011	8806	0	24	270470
Peru	2012	Peru Continuous Demographic and Health Survey 2012	9266	0	24	270471
Peru	2013	Peru Continuous Demographic and Health Survey 2013	9345	0	24	146860
Peru	2014	Peru Continuous Demographic and Health Survey 2014	9932	0	24	209930
Peru	2015	Peru Demographic and Family Health Survey 2015	23242	1614	0	303663
Peru	2016	Peru Demographic and Family Health Survey 2016	20606	1916	0	303664
Peru	2017	Peru Demographic and Family Health Survey 2017	22334	1878	0	358824
Peru	2018	Peru Demographic and Family Health Survey 2018	23992	3815	0	407869
Republic of the Congo	2005	Congo Demographic and Health Survey 2005	4118	0	12	19391
Republic of the Congo	2011–2012	Congo Demographic and Health Survey 2011–2012	4549	0	12	56151
Republic of the Congo	2014–2015	Congo Multiple Indicator Cluster Survey 2014–2015	8893	0	11	234733
Rwanda	2000	Rwanda Demographic and Health Survey 2000	6429	0	12	20722
Rwanda	2000	Rwanda Multiple Indicator Cluster Survey 2000	2848	0	12	26930
Rwanda	2001	Rwanda Integrated Household Living Conditions Survey 1999–2001	4075	0	12	11319
Rwanda	2005	Rwanda Demographic and Health Survey 2005	3775	455	0	20740
Rwanda	2006	Rwanda Comprehensive Food Security and Vulnerability Assessment 2006	1869	0	281	58185
Rwanda	2010–2011	Rwanda Demographic and Health Survey 2010–2011	4145	492	0	56040
Rwanda	2012	Rwanda Comprehensive Food Security and Vulnerability Assessment and Nutrition Survey 2012	4446	0	743	151436
Rwanda	2014–2015	Rwanda Demographic and Health Survey 2014–2015	3617	491	0	157063
Sao Tome and Principe	2000	Sao Tome and Principe Multiple Indicator Cluster Survey 2000	1842	0	4	27055

Country	Survey year(s)	Survey Name	Number of Individuals	Number of geo-positioned clusters	Number of polygons (areal)	GHDx NID
Sao Tome and Principe	2008–2009	Sao Tome and Principe Demographic and Health Survey 2008–2009	1719	0	7	26866
Sao Tome and Principe	2014	Sao Tome and Principe Multiple Indicator Cluster Survey 2014	1965	0	7	214640
Senegal	2000	Senegal Multiple Indicator Cluster Survey 2000	8703	0	10	27044
Senegal	2005	Senegal Demographic and Health Survey 2005	2867	360	0	26855
Senegal	2010–2011	Senegal Demographic and Health Survey 2010–2011	3872	385	0	56063
Senegal	2012–2013	Senegal Continuous Demographic and Health Survey 2012–2013	6100	200	0	111432
Senegal	2014	Senegal Continuous Demographic and Health Survey 2014	6025	196	0	191270
Senegal	2015	Senegal Continuous Demographic and Health Survey 2015	6257	214	0	218592
Senegal	2016	Senegal Continuous Demographic and Health Survey 2016	6085	214	0	286772
Senegal	2015–2016	Senegal - Dakar Urban Multiple Indicator Cluster Survey 2015–2016	4234	0	4	287639
Senegal	2017	Senegal Continuous Demographic and Health Survey 2017	10841	0	14	353526
Sierra Leone	2000	Sierra Leone Multiple Indicator Cluster Survey 2000	2480	0	4	11639
Sierra Leone	2005	Sierra Leone Multiple Indicator Cluster Survey 2005	5229	0	14	11649
Sierra Leone	2008	Sierra Leone Demographic and Health Survey 2008	2289	340	0	21258
Sierra Leone	2010	Sierra Leone Multiple Indicator Cluster Survey 2010	8422	0	14	76700
Sierra Leone	2013	Sierra Leone Demographic and Health Survey 2013	4806	434	0	131467
Sierra Leone	2017	Sierra Leone Multiple Indicator Cluster Survey 2017	11785	0	14	218619
Somalia	2006	Somalia Multiple Indicator Cluster Survey 2006	5867	0	18	11774
South Africa	2002	South Africa - Agincourt Integrated Family Survey 2002	197	0	1	135825
South Africa	2004	South Africa KwaZulu-Natal Income Dynamics Study 2004	794	0	1	31142
South Africa	2004	South Africa - Agincourt Integrated Family Survey 2004	301	0	1	135826
South Africa	2008	South Africa National Income Dynamics Study - Wave 1 2008	2161	0	47	27885
South Africa	2010–2011	South Africa National Income Dynamics Study - Wave 2 2010–2011	1700	0	55	133731

Country	Survey year(s)	Survey Name	Number of Individuals	Number of geo-positioned clusters	Number of polygons (areal)	GHDx NID
South Africa	2012	South Africa National Income Dynamics Study - Wave 3 2012	3190	0	52	133732
South Africa	2014–2015	South Africa National Income Dynamics Study - Wave 4 2014–2015	3878	0	52	265153
South Africa	2016	South Africa National Income Dynamics Study - Wave 5 2017	1139	475	0	157064
South Africa	2017	South Africa Demographic and Health Survey 2016	3874	0	52	369644
South Sudan	2000	Sudan - South Multiple Indicator Cluster Survey 1999	1130	0	45	12232
South Sudan	2010	Sudan - South Multiple Indicator Cluster Survey 2010	6807	0	10	32189
Sri Lanka	1999–2000	Sri Lanka Integrated Survey 1999–2000	2037	455	0	12201
Sudan	2000	Sudan Multiple Indicator Cluster Survey 2000	20561	0	16	12243
Sudan	2010	Sudan - North Multiple Indicator Cluster Survey 2010	12296	0	15	153643
Sudan	2014	Sudan Multiple Indicator Cluster Survey 2014	12999	0	18	200617
Suriname	1999–2000	Suriname Multiple Indicator Cluster Survey 1999–2000	1811	0	10	12280
Suriname	2006	Suriname Multiple Indicator Cluster Survey 2006	2017	0	5	12289
Suriname	2010	Suriname Multiple Indicator Cluster Survey 2010	2859	0	10	81203
Swaziland	2000	Swaziland Multiple Indicator Cluster Survey 2000	3411	0	4	12320
Swaziland	2006–2007	Swaziland Demographic and Health Survey 2006–2007	2080	268	0	20829
Swaziland	2010	Swaziland Multiple Indicator Cluster Survey 2010	2590	0	4	30325
Swaziland	2014	Swaziland Multiple Indicator Cluster Survey 2014	2668	0	4	200707
Syria	2006	Syria Multiple Indicator Cluster Survey 2006	10784	0	60	12399
Syria	2009	Syria Family Health Survey 2009	15305	0	14	126911†
Tajikistan	2005	Tajikistan Multiple Indicator Cluster Survey 2005	4239	0	5	12608
Tajikistan	2007	Tajikistan Living Standards Measurement Survey 2007	149	18	0	12584
Tajikistan	2012	Tajikistan Demographic and Health Survey 2012	4568	341	0	74460
Tajikistan	2017	Tajikistan Demographic and Health Survey 2017	5902	365	0	341838
Tanzania	2004	Tanzania - Kagera Living Standards Measurement Study 2004	1967	894	0	14341
Tanzania	2004–2005	Tanzania Demographic and Health Survey 2004–2005	7344	0	26	20875

Country	Survey year(s)	Survey Name	Number of Individuals	Number of geo-positioned clusters	Number of polygons (areal)	GHDx NID
Tanzania	2006–2007	Tanzania Core Welfare Indicators Questionnaire Survey 2006–2007	9989	0	28	31831
Tanzania	2009–2010	Tanzania Demographic and Health Survey 2009–2010	6766	458	0	21331
Tanzania	2010–2011	Tanzania National Panel Survey 2010–2011	2741	0	126	81005
Tanzania	2012–2013	Tanzania National Panel Survey 2012–2013	3364	0	135	224096†
Tanzania	2015–2016	Tanzania Demographic and Health Survey 2015–2016	9104	607	0	218593
Tanzania	2014–2016	Tanzania National Panel Survey 2014–2016	2463	417	0	311265
Thailand	2005–2006	Thailand Multiple Indicator Cluster Survey 2005–2006	9207	0	4	12732
Thailand	2012	Thailand Multiple Indicator Cluster Survey 2012	9246	0	5	148649
Thailand	2015–2016	Thailand Multiple Indicator Cluster Survey 2015–2016	11368	0	5	296646
Thailand	2016	Thailand - Bangkok Small Community Multiple Indicator Cluster Survey 2016	992	0	1	331377
The Gambia	2000	Gambia Multiple Indicator Cluster Survey 2000	3465	0	8	3922
The Gambia	2005–2006	Gambia Multiple Indicator Cluster Survey 2005–2006	6465	0	36	3935
The Gambia	2010	Gambia Multiple Indicator Cluster Survey 2010	11574	0	6	91506
The Gambia	2013	Gambia Demographic and Health Survey 2013	3408	0	37	77384
Timor-Leste	2003	Timor-Leste Demographic and Health Survey 2003	5321	287	92	20888†
Timor-Leste	2007–2008	Timor-Leste Living Standards and Measurement Survey 2007–2008	3531	0	64	46682†
Timor-Leste	2009–2010	Timor-Leste Demographic and Health Survey 2009–2010	8494	0	13	21274
Timor-Leste	2016	Timor-Leste Demographic and Health Survey 2016	6455	455	0	286785
Togo	2006	Togo Multiple Indicator Cluster Survey 2006	4018	0	6	12896
Togo	2010	Togo Multiple Indicator Cluster Survey 2010	4711	0	6	40021
Togo	2013	Togo Demographic and Health Survey 2013–2014	3238	328	0	77515
Trinidad and Tobago	2000	Trinidad and Tobago Multiple Indicator Cluster Survey 2000	806	0	15	12940
Trinidad and Tobago	2011	Trinidad and Tobago Multiple Indicator Cluster Survey 2011	1122	0	5	332558

Country	Survey year(s)	Survey Name	Number of Individuals	Number of geo-positioned clusters	Number of polygons (areal)	GHDx NID
Tunisia	2011–2012	Tunisia Multiple Indicator Cluster Survey 2011–2012	2758	0	9	76709
Tunisia	2018	Tunisia Multiple Indicator Cluster Survey 2018	3366	0	7	409558
Turkmenistan	2006	Turkmenistan Multiple Indicator Cluster Survey 2006	2048	0	6	13064
Turkmenistan	2015–2016	Turkmenistan Multiple Indicator Cluster Survey 2015–2016	3741	0	6	264583
Uganda	2000–2001	Uganda Demographic and Health Survey 2000–2001	4791	269	0	20993
Uganda	2006	Uganda Demographic and Health Survey 2006	2242	333	0	21014
Uganda	2009–2010	Uganda Living Standards Measurement Survey - Integrated Survey on Agriculture 2009–2010	1476	282	7	81004
Uganda	2011	Uganda Demographic and Health Survey 2011	2097	392	0	56021
Uganda	2010–2011	Uganda Living Standards Measurement Survey - Integrated Survey on Agriculture 2010–2011	1641	415	0	142934
Uganda	2011–2012	Uganda Living Standards Measurement Survey - Integrated Survey on Agriculture 2011–2012	1590	455	0	142935
Uganda	2013–2014	Uganda Living Standards Measurement Survey - Integrated Survey on Agriculture 2013–2014	1611	0	358	264959
Uganda	2016	Uganda Demographic and Health Survey 2016	4414	679	0	286780
Uzbekistan	2002	Uzbekistan Special Demographic and Health Survey 2002	2664	218	0	21039
Uzbekistan	2006	Uzbekistan Multiple Indicator Cluster Survey 2006	4925	0	6	13445
Vietnam	2000	Vietnam Multiple Indicator Cluster Survey 2000	3045	0	8	13708
Vietnam	2001–2002	Vietnam National Health Survey 2001–2002	11225	0	61	44586†
Vietnam	2010–2011	Vietnam Multiple Indicator Cluster Survey 2010–2011	3615	0	590	57999
Yemen	2005–2006	Yemen Household Budget Survey 2005–2006	12672	0	495	22882
Yemen	2012	Yemen - Aden Nutritional Status and Mortality Survey 2012	1120	0	8	244469
Yemen	2012	Yemen - Hajjah Nutritional Status and Mortality Survey 2012	1300	56	0	244471
Yemen	2012	Yemen - Rayma Nutritional Status and Mortality Survey 2012	639	0	6	244472
Yemen	2012	Yemen - Taiz Nutritional Status and Mortality Survey 2012	904	37	0	244473
Yemen	2012	Yemen - Ibb Nutritional Status and Mortality Survey 2012	1651	55	0	246249

Country	Survey year(s)	Survey Name	Number of Individuals	Number of geo-positioned clusters	Number of polygons (areal)	GHDx NID
Yemen	2012	Yemen - Lahj Nutritional Status and Mortality Survey 2012	1560	0	1	246254
Yemen	2013	Yemen Demographic and Health Survey 2013	14569	0	528	112500†
Yemen	2012–2013	Yemen - Abyan Nutritional Status and Mortality Survey 2012–2013	1442	32	0	246145
Yemen	2013	Yemen - Dhamar Nutritional Status and Mortality Survey 2013	1877	40	0	246209†
Yemen	2013	Yemen - Mahweet Nutritional Status and Mortality Survey 2013	1626	67	0	246250
Yemen	2012–2013	Yemen National Social Protection Monitoring Survey 2012–2013	23803	306	194	249499†
Yemen	2014	Yemen Comprehensive Food Security Survey 2014	13438	0	359	244480
Yemen	2014	Yemen - Hajjah Nutritional Status and Mortality Survey 2014	565	60	0	246246
Yemen	2014	Yemen - Hodeidah Nutritional Status and Mortality Survey 2014	1412	37	0	246248
Yemen	2015	Yemen - Aden Nutritional Status and Mortality Survey 2015	336	0	6	244463
Yemen	2015	Yemen - Al-Baidha Nutritional Status and Mortality Survey 2015	628	27	16	244464
Yemen	2015	Yemen - Hajjah Nutritional Status and Mortality Survey 2015	1018	45	0	244465
Yemen	2015	Yemen - Hodeidah Nutrition and Mortality Survey 2015	648	0	17	244467
Yemen	2015	Yemen - Lahj Nutritional Status and Mortality Survey 2015	978	0	1	244468
Zambia	2001–2002	Zambia Demographic and Health Survey 2001–2002	5684	0	72	21102
Zambia	2007	Zambia Demographic and Health Survey 2007	5450	319	0	21117
Zambia	2009	Zambia Access to ACT Initiative Survey 2009	1332	1322	0	162031†
Zambia	2013–2014	Zambia Demographic and Health Survey 2013–2014	11924	719	0	77516
Zimbabwe	2005–2006	Zimbabwe Demographic and Health Survey 2005–2006	4239	394	0	21163
Zimbabwe	2009	Zimbabwe Multiple Indicator Monitoring Survey 2009	6282	0	10	35493
Zimbabwe	2010–2011	Zimbabwe Demographic and Health Survey 2010–2011	4316	393	0	55992
Zimbabwe	2014	Zimbabwe Multiple Indicator Cluster Survey 2014	9651	0	10	152720
Zimbabwe	2015	Zimbabwe Demographic and Health Survey 2015	5040	399	0	157066

Supplementary Table 4: Survey Reports added to model.

Country	Survey year(s)	Survey Name	Number of Individuals	Number of geo-positioned clusters	Number of polygons (areal)	GHDx NID
Burkina Faso	2009	Burkina Faso National Nutrition Survey 2009	265,498	0	43	56884
Burkina Faso	2016	Burkina Faso National Nutrition Survey 2016	108,449	0	13	299307
China	2002	China National Nutrition Survey 2002 - China CDC	27,525	0	128	124479†
China	2009–2015	Secular Trends in Growth and Nutritional Outcomes of Children under Five Years Old in Xiamen, China	71,229	0	3	398585
Mauritania	2012	Mauritania National Nutrition Survey Using SMART Methodology July 2012	163,663	0	11	275121
Mauritania	2014	Mauritania National Nutrition Survey Using the SMART Methodology August 2014	166,139	0	11	275123
Mongolia	2004	Mongolia National Nutrition Survey 2004	45,316	0	21	137528†
Niger	2007	Niger Nutrition and Child Survival Survey 2007	454,450	0	8	160103
Niger	2008	Niger Nutrition and Child Survival Survey 2008	276,775	0	8	160198
Niger	2009	Niger Nutrition and Child Survival Survey 2009	470,951	0	8	160053
Niger	2011	Niger Nutrition and Child Survival Survey 2011	489,926	0	8	316438
Niger	2012	Niger Nutrition and Child Survival Survey 2012	597,220	0	8	316440
Niger	2013	Niger Nutrition and Child Survival Survey 2013	796,291	0	8	316442
Niger	2014	Niger Nutrition and Child Survival Survey 2014	975,149	0	8	316444
Nigeria	2010	Nigeria Standardized Monitoring and Assessment of Relief and Transitions Survey, December 2010	73,134	0	8	151724†
Nigeria	2011	Nigeria Standardized Monitoring and Assessment of Relief and Transitions Survey, July-August 2011	88,246	0	7	151725†
Nigeria	2012	Nigeria Standardized Monitoring and Assessment of Relief and Transitions Survey, February-March 2012	104,742	0	8	151727†
Nigeria	2014	Nigeria National Nutrition and Health Survey 2014	260,912	0	37	274708
Nigeria	2015	Nigeria National Nutrition and Health Survey 2015	264,297	0	37	274707

Country	Survey year(s)	Survey Name	Number of Individuals	Number of geo-positioned clusters	Number of polygons (areal)	GHDx NID
Nigeria	2018	Nigeria Demographic and Health Survey 2018	11,204	0	37	408484
Pakistan	2007–2008	Pakistan - Punjab Multiple Indicator Cluster Survey 2007–2008	71,721	0	175	387340
Sri Lanka	2009	Sri Lanka Nutrition and Food Security Assessment 2009	10,969	0	20	141592
Sri Lanka	2012	Sri Lanka National Nutrition and Micronutrient Survey 2012	4,753	0	10	153000
Sri Lanka	2016	Sri Lanka Demographic and Health Survey 2016	12,337	0	25	326837
Sri Lanka	2006–2007	Sri Lanka Demographic and Health Survey 2006–2007	13,528	0	25	18815
eSwatini	2008	Swaziland National Nutrition Survey 2008	10,376	0	4	141312
Tajikistan	2001	Tajikistan National Nutrition Survey 2001	46,075	0	4	141797
Thailand	2015–2016	Thailand 14 Provinces Multiple Indicator Cluster Survey 2015–2016	32,485	0	14	317305
Tunisia	2000	Tunisia Multiple Indicator Cluster Survey 2000	78,685	0	6	12983
Tunisia	2006	Tunisia Multiple Indicator Cluster Survey 2006	24,013	0	9	12985
Turkmenistan	2000	Turkmenistan Demographic and Health Survey 2000	148,724	0	6	20956
Venezuela	2016	Venezuela - Capital District, Vargas, Miranda and Zulia Baseline of Sentinel Monitoring of Nutrition Status in Children Under 5 Years, SAMAN System October-December 2016	8,622	0	4	289278
Vietnam	2009–2010	Vietnam General Nutrition Survey 2009–2010	286,085	0	63	152422
Vietnam	2011	Vietnam Annual National Nutrition Monitoring 2011	292,176	0	63	293979†
Vietnam	2012	Vietnam Nutrition Surveillance 2012	297,529	0	63	286277
Vietnam	2012	Vietnam Annual National Nutrition Monitoring 2012	293,659	0	63	293980†
Vietnam	2013	Vietnam Annual National Nutrition Monitoring 2013	299,685	0	63	293981†

Country	Survey year(s)	Survey Name	Number of Individuals	Number of geo-positioned clusters	Number of polygons (areal)	GHDx NID
Vietnam	2014	Vietnam Annual National Nutrition Monitoring 2014	98,433	0	63	293982†

Supplementary Table 5: Data excluded from model.

Country	Survey year(s)	Survey Name	Reason	GHDx NID
Afghanistan	2015–2016	*Afghanistan Demographic and Health Survey 2015–2016*	Age data is insufficiently granular (for calculations of height-for-age and weight-for-age z-scores)	157018
Bangladesh	2010–2013	*Bangladesh - Dhaka Malnutrition and Enteric Disease Study 2009–2014*	Anthropometric measurements not taken of a full set of under-fives	261683
Bangladesh	2000	*Bangladesh - Khulna and Dhaka Supporting Household Activities for Health, Assets, and Revenue Survey 2000*	Data does not have interview dates or age in months	153154
Bangladesh	2015	*Bangladesh Integrated Household Survey 2015*	Data does not have interview dates or age in months, insufficient sample weight data	283269
Bangladesh	2011–2012	*Bangladesh Integrated Household Survey 2011–2012*	Insufficient sample weight data	153062
Bangladesh	2002	*Bangladesh - Dinajpur Supporting Household Activities for Health, Assets, and Revenue Survey, Round 1 2002*	Insufficient sample weight data	163056
Bangladesh	2007–2011	*Bangladesh - Mizrapur Global Enteric Multicenter Study 2007–2011*	Insufficient sample weight data	224248
Bangladesh	2011–2013	*Bangladesh - Mizrapur Global Enteric Multicenter Study 2011–2013*	Insufficient sample weight data	224855
Bangladesh	2003	*Bangladesh - Dinajpur Supporting Household Activities for Health, Assets, and Revenue Survey, Round 2 2003*	Insufficient sample weight data	231832
Bangladesh	2003	*Bangladesh - Dinajpur Supporting Household Activities for Health, Assets, and Revenue Survey, Round 3 2003*	Insufficient sample weight data	231835
Bangladesh	2014–2017	*Bangladesh - Dhaka Cohort Study of Cryptosporidiosis in Children 2014*	Insufficient sample weight data	263389
Benin	2011–2012	*Benin Demographic and Health Survey 2011–2012*	Prevalence values for indicators were determined to be implausible	79839

Country	Survey year(s)	Survey Name	Reason	GHDx NID
Brazil	2010–2013	*Brazil - Fortaleza Malnutrition and Enteric Disease Study 2009–2014*	Anthropometric measurements not taken of a full set of under-fives	261873
Burkina Faso	2005	*Burkina Faso Core Welfare Indicators Questionnaire Survey 2005*	National prevalence values reported for one or more indicators were determined to be implausibly high based on country-level trend seen in 8 other Burkina Faso sources.	22950
Cameroon	2001	*Cameroon Household Survey 2001*	Anthropometric measurements not taken of a full set of under-fives	2039
Egypt	2015	*Egypt Special Demographic and Health Survey 2015*	Non-proportional sample allocation designed to estimate the prevalence of hepatitis and certain other NCD risk factors, such that the survey sampling was not comparable to the other surveys.	157026
Ethiopia	2004	*Ethiopia Rural Household Survey 2004*	Age data is insufficiently granular (for calculations of height-for-age and weight-for-age z-scores)	38496
Ghana	2009–2010	*Ghana Socioeconomic Panel Survey 2009–2010*	National prevalence values reported for one or more indicators were determined to be implausibly high based on country-level trend seen in 8 other country-level Ghana sources.	236205
India	2011–2013	*India Human Development Survey 2011–2013*	Sample size is not representative of its geography	165498
India	2010–2013	*India - Vellore Malnutrition and Enteric Disease Study 2009–2014*	Anthropometric measurements not taken of a full set of under-fives	261875
India	2007–2008	*India Tribal Second Repeat Survey of Diet and Nutritional Status 2007–2008*	Geographies could not be mapped	129783
Kenya	2004	*Kenya Greater Eldoret Health and Development Survey 2004*	Insufficient sample weight data	152561
Kenya	2005	*Kenya Greater Eldoret Health and Development Survey 2005*	Insufficient sample weight data	152562

Country	Survey year(s)	Survey Name	Reason	GHDx NID
Kenya	2006	*Kenya Greater Eldoret Health and Development Survey 2006*	Insufficient sample weight data	152563
Kenya	2008–2011	*Kenya - Nyanza Global Enteric Multicenter Study 2008–2011*	Insufficient sample weight data	224239
Kenya	2011–2012	*Kenya - Nyanza Global Enteric Multicenter Study 2011–2012*	Insufficient sample weight data	224853
Lebanon	2005–2006	*Palestinians in Lebanon Multiple Indicator Cluster Survey 2005–2006*	Data is not representative of its geography	7688
Lebanon	2011	*Palestinians in Lebanon Multiple Indicator Cluster Survey 2011*	Data is not representative of its geography	76708
Mali	2007–2011	*Mali - Bamako Global Enteric Multicenter Study 2007–2011*	Insufficient sample weight data	224233
Mali	2011–2013	*Mali - Bamako Global Enteric Multicenter Study 2011–2013*	Insufficient sample weight data	224848
Mexico	2002	*Mexico Family Life Survey 2002*	Age data is insufficiently granular (for calculations of height-for-age and weight-for-age z-scores)	8442
Mexico	2015	*Mexico Multiple Indicator Cluster Survey 2015*	Geographies could not be mapped	264590
Mozambique	2007–2011	*Mozambique - Manhiça Global Enteric Multicenter Study 2007–2011*	Insufficient sample weight data	224236
Mozambique	2011–2013	*Mozambique - Manhiça Global Enteric Multicenter Study 2011–2013*	Insufficient sample weight data	224849
Nepal	2010–2013	*Nepal - Bhaktapur Malnutrition and Enteric Disease Study 2009–2014*	Anthropometric measurements not taken of a full set of under-fives	261880
Nigeria	2008–2010	*Nigeria Living Standards Survey 2008–2010*	Age data is insufficiently granular (for calculations of height-for-age and weight-for-age z-scores)	151719
Nigeria	2010–2011	*Nigeria General Household Survey 2010–2011*	Age data is insufficiently granular (for calculations of height-for-age and weight-for-age z-scores)	151802

Country	Survey year(s)	Survey Name	Reason	GHDx NID
Nigeria	2006	*Nigeria Core Welfare Indicators Questionnaire Survey 2006*	Insufficient sample weight data	9522
Nigeria	2011	*Nigeria - Akwa Ibom Survey on Dietary Intakes, Vitamin A, and Iron Status of Women of Childbearing Age and Children 6-59 Months of Age 2011*	Insufficient sample weight data	283272
Pakistan	2010–2013	*Pakistan - Naushahro Feroze Malnutrition and Enteric Disease Study 2009–2014*	Anthropometric measurements not taken of a full set of under-fives	261883
Pakistan	2008–2011	*Pakistan - Karachi Global Enteric Multicenter Study 2008–2011*	Insufficient sample weight data	224251
Pakistan	2011–2013	*Pakistan - Karachi Global Enteric Multicenter Study 2011–2013*	Insufficient sample weight data	224856
Peru	2010–2013	*Peru - Loreto Malnutrition and Enteric Disease Study 2009–2014*	Anthropometric measurements not taken of a full set of under-fives	261879
Rwanda	2009	*Rwanda Comprehensive Food Security and Vulnerability Assessment and Nutrition Survey 2009*	Anthropometric measurements not taken of a full set of under-fives	58188
Somalia	2007	*Somalia Nutrition Surveillance and Assessment 2007*	Data is duplicative	358676
Somalia	2008	*Somalia Nutrition Surveillance and Assessment 2008*	Data is duplicative	358679
Somalia	2009	*Somalia Nutrition Surveillance and Assessment 2009*	Data is duplicative	358680
Somalia	2010	*Somalia Nutrition Surveillance and Assessment 2010*	Data is duplicative	358681
Somalia	2001	*Somalia Nutrition Surveillance and Assessment 2001–2006*	Geographies could not be mapped	358670-358675
South Africa	2010–2013	*South Africa - Venda Malnutrition and Enteric Disease Study 2009–2014*	Anthropometric measurements not taken of a full set of under-fives	261887
South Sudan	2009	*Sudan - South National Baseline Household Survey (NBHS) 2009*	Data does not have interview dates or age in months	30368
Tanzania	2008–2009	*Tanzania National Panel Survey 2008–2009*	Age data is insufficiently granular (for calculations of height-for-age and weight-for-age z-scores)	27297
Tanzania	2004	*Tanzania - Shinyanga Core Welfare Indicators Questionnaire Survey 2004*	Age data is insufficiently granular (for calculations of height-for-age and weight-for-age z-scores)	31786

Country	Survey year(s)	Survey Name	Reason	GHDx NID
Tanzania	2005	*Tanzania Core Welfare Indicators Questionnaire Survey 2005*	Age data is insufficiently granular (for calculations of height-for-age and weight-for-age z-scores)	31797
Tanzania	2010–2013	*Tanzania - Haydom Malnutrition and Enteric Disease Study 2009–2014*	Anthropometric measurements not taken of a full set of under-fives	261889
Tanzania	2010	*Tanzania - Kagera Living Standards Measurement Study 2010*	Data is not representative of its geography	93807
The Gambia	2007–2011	*Gambia - Basse Global Enteric Multicenter Study 2007–2011*	Insufficient sample weight data	222752
The Gambia	2011–2013	*Gambia - Basse Global Enteric Multicenter Study 2011–2013*	Insufficient sample weight data	223566

2.3 Covariates

A variety of environmental and socioeconomic variables were used to predict overweight and wasting outcomes. Where available, the finest spatiotemporal resolution of gridded datasets was used. In addition to the covariates detailed below, country-level variables of lag distributed income per capita and the proportion of the population with access to adequate sanitation were included in models for overweight and wasting.

Supplementary Table 6: Covariates used in mapping.

Covariate	Temporal resolution	Source	Reference
Travel time to nearest settlement >50,000 inhabitants* **(1)**	Static	Big Data Institute, Nuffield Department of Medicine, University of Oxford	Weiss, D. J. *et al*. A global map of travel time to cities to assess inequalities in accessibility in 2015. *Nature* **533**, 333-336 (2018).

**Covariate**	**Temporal resolution**	**Source**	**Reference**
Average daily mean rainfall (Precipitation) **(2)**	Annual	CRUTS	Harris, I., Jones, P. d., Osborn, T. j. & Lister, D. h. Updated high-resolution grids of monthly climatic observations – the CRU TS3.10 dataset. *Int. J. Climatol*. **34**, 623–642 (2014). University of East Anglia. Climatic Research Unit TS v. 3.24 dataset. Available at: https://crudata.uea.ac.uk/cru/data/hrg/cru_ts_3.24.01/. (Accessed: 24th July 2017).
Educational attainment in women of reproductive age (15–49 years old) **(3)**	Annual	Institute for Health Metrics and Evaluation, University of Washington	Graetz, N., *et al.* Mapping disparities in education across low- and middle-income countries. *Nature* **577,** 235–238 (2020).
Enhanced Vegetation Index (EVI) **(4)**	Annual	MODIS	Huete, A., Justice, C. & van Leeuwen, W. MODIS vegetation index (MOD 13) algorithm theoretical basis document. (1999).<br>USGS & NASA. Vegetation indices 16-Day L3 global 500m MOD13A1 dataset. Available at: https://lpdaac.usgs.gov/products/mod13a1v006/. (Accessed: 25th July 2017)<br>Weiss, D. J. et al. An effective approach for gap-filling continental scale remotely sensed time-series. *Isprs J. Photogramm. Remote Sens*. **98**, 106–118 (2014).

Covariate	Temporal resolution	Source	Reference
Fertility<br>**(5)**	Annual	WorldPop (derived)	Lloyd, C. T., Sorichetta, A. & Tatem, A. J. High resolution global gridded data for use in population studies. *Sci. Data* **4**, sdata20171 (2017).<br>World Pop. Get data. Available at: http://www.worldpop.org.uk/data/get_data/. (Accessed: 25th July 2017)
Growing season length*<br>**(6)**	Static	FAO	FAO. GAEZ - Global Agro-Ecological Zones data portal. Available at: http://www.fao.org/nr/gaez/about-data-portal/en/. (Accessed: 25th July 2017)<br>FAO. GAEZ - Global Agro-Ecological Zones users guide. (2012).
Urbanicity<br>**(7)**	Annual	European Commission/GHS	Pesaresi, M. et al. Operating procedure for the production of the Global Human Settlement Layer from Landsat data of the epochs 1975, 1990, 2000, and 2014. (Publications Office of the European Union, 2016).
Nutritional yield for vitamin A*<br>**(8)**	Static	Herrero et al (modelled)	Herrero, M. et al. Farming and the geography of nutrient production for human use: a transdisciplinary analysis. *Lancet Planet. Health* **1**, e33–e42 (2017).
Nutritional yield for protein*<br>**(9)**	Static	Herrero et al (modelled)	Herrero, M. et al. Farming and the geography of nutrient production for human use: a transdisciplinary analysis. *Lancet Planet. Health* **1**, e33–e42 (2017).
Irrigation*<br>**(10)**	Static	University of Frankfurt	Goethe-Universität. Generation of a digital global map of irrigation areas. Available at: https://www.uni-frankfurt.de/45218039/Global_Irrigation_Map. (Accessed: 25th July 2017)

Covariate	Temporal resolution	Source	Reference
Nutritional yield for Iron* **(11)**	Static	Herrero et al (modelled)	Herrero, M. et al. Farming and the geography of nutrient production for human use: a transdisciplinary analysis. *Lancet Planet. Health* **1**, e33–e42 (2017).
Population **(12)**	Annual	WorldPop	Lloyd, C. T., Sorichetta, A. & Tatem, A. J. High resolution global gridded data for use in population studies. *Sci. Data* **4**, sdata20171 (2017).<br><br>World Pop. Get data. Available at: http://www.worldpop.org.uk/data/get_data/. (Accessed: 25th July 2017)
*Temporally dynamic covariates which were reformatted as a synoptic mean over each estimation period or as a mid-period year estimate.			

Supplementary Table 7: Model prior sensitivity analyses. The models included 420 geo-referenced household surveys representing over 3 million under-5 children. Pearson correlation method was used to compute correlation values.

Indicator	Model results compared	Correlation			Mean absolute error			90th percentile absolute difference			Percent of overlapping uncertainty intervals		
		Grid cell	Admin 1	Admin 2	Grid cell	Admin 1	Admin 2	Grid cell	Admin 1	Admin 2	Grid cell	Admin 1	Admin 2
Overweight	Model 2	0.991	0.995	0.995	0.163	0.317	0.372	0.918	0.764	0.884	100	100	100
	Model 3	0.994	0.996	0.997	0.218	0.240	0.322	0.618	0.536	0.722	100	100	100
	Model 4	0.990	0.991	0.991	0.263	0.317	0.372	0.918	0.764	0.884	100	100	100
	Model 5	0.995	0.998	0.997	0.220	0.240	0.322	0.618	0.536	0.722	100	100	100
Wasting	Model 2	0.997	0.998	0.998	0.173	0.237	0.382	0.817	0.664	0.784	100	100	100
	Model 3	0.998	0.999	0.997	0.118	0.231	0.332	0.755	0.666	0.762	100	100	100
	Model 4	0.999	0.998	0.998	0.163	0.227	0.372	0.786	0.752	0.894	100	100	100
	Model 5	0.999	0.998	0.997	0.120	0.244	0.322	0.708	0.588	0.755	100	100	100
AROC	Model 2	0.998	0.998	0.998	0.126	0.287	0.373	0.968	0.732	0.899	100	100	100
	Model 3	0.998	0.999	0.999	0.168	0.321	0.368	0.788	0.636	0.912	100	100	100
	Model 4	0.994	0.997	0.998	0.195	0.347	0.375	0.891	0.954	1.250	100	100	100
	Model 5	0.996	0.998	0.997	0.175	0.348	0.382	0.918	0.936	1.172	100	100	100

Supplementary Table 8: Spatial hyperparameter priors by region.

Region	μ_{θ_1}	$\sigma^2_{\theta_1}$	μ_2	$\sigma^2_{\theta_2}$
Andean South America	0.011191	10	-1.2767	10
Central America and the Caribbean	0.209786	10	-1.4753	10
Central sub-Saharan Africa	-0.20487	10	-1.06064	10
East Asia	0.377015	10	-1.64253	10
Eastern sub-Saharan Africa	0.137024	10	-1.40254	10
Middle East	-0.22044	10	-1.04507	10
North Africa	0.296578	10	-1.56209	10
Southeast Asia and Oceania	0.423677	10	-1.68919	10
South and Central Asia	0.122237	10	-1.38775	10
Southern sub-Saharan Africa	-0.41501	10	-0.8505	10
Tropical South America	0.130726	10	-1.39624	10
Western sub-Saharan Africa	0.201186	10	-1.4667	10

3.0 Model Results

Supplementary Table 9: Overweight fitted parameters.

Lower, median, and upper quantiles (percentiles 0.025, 0.50, 0.975) are displayed for the main parameters from the overweight models by region. The fixed effects covariates corresponding to the predicted ensemble rasters are shown in the first five columns, while fitted values for the spatiotemporal field hyperparameters and the precisions (inverse variance) for our random effects are shown in the last five columns.

Regions	Percentiles	int	gam	gbm	lasso	Nominal Range	Nominal Variance	AR1 rho	Precision for NUG.ID	Precision for CTRY.ID
**North Africa**	**0.025**	-0.69	0.00	0.64	-0.01	1.79	0.16	0.01	4.93	2.10
	**0.50**	-0.43	0.12	0.76	0.12	2.40	0.22	0.45	5.70	6.04
	**0.975**	-0.17	0.25	0.88	0.25	3.15	0.27	0.66	6.74	20.06
**Central sub-Saharan Africa**	**0.025**	-0.28	-0.03	0.26	0.29	2.82	0.17	0.39	8.71	5.97
	**0.50**	-0.11	0.11	0.42	0.47	3.90	0.22	0.66	11.86	17.90
	**0.975**	0.06	0.25	0.59	0.65	5.22	0.30	0.80	16.29	53.58
**Middle East**	**0.025**	-0.58	-0.05	-0.14	0.60	2.03	0.28	0.54	2.91	$1.2\ x10^{2}$
	**0.50**	-0.28	0.11	0.09	0.80	3.19	0.47	0.80	3.55	$1.4\ x\ 10^{2}$
	**0.975**	0.02	0.27	0.32	1.00	5.06	0.81	0.92	4.34	6.9 x 10^{2}
**Central America and the Caribbean**	**0.025**	-0.18	-0.15	0.13	0.34	1.99	0.07	0.58	10.56	5.23
	**0.50**	0.01	0.08	0.34	0.57	3.40	0.11	0.81	15.31	17.73
	**0.975**	0.20	0.31	0.56	0.80	5.69	0.18	0.92	23.05	65.25
**Western sub-Saharan Africa**	**0.025**	-0.38	-0.18	0.23	0.58	3.75	0.25	0.54	2.65	4.10
	**0.50**	-0.19	-0.06	0.34	0.72	4.53	0.31	0.71	2.87	8.73
	**0.975**	0.01	0.06	0.45	0.85	5.78	0.39	0.80	3.12	18.82
**Andean South America**	**0.025**	0.07	0.32	0.74	-0.31	0.00	0.00	0.65	1.1 x 10^{2}	15.92
	**0.50**	0.14	0.40	0.83	-0.22	0.06	0.00	0.97	1.7 x 10^{3}	52.81
	**0.975**	0.21	0.48	0.91	-0.14	0.33	0.00	1.00	3.0 x 10^{5}	131.15
**Southern sub-Saharan Africa**	**0.025**	-0.21	0.13	0.03	0.33	3.83	0.06	0.30	1.5 x 10^{2}	8.56
	**0.50**	-0.03	0.30	0.20	0.50	5.80	0.10	0.67	1.4 x 10^{3}	27.24
	**0.975**	0.15	0.48	0.37	0.67	8.77	0.16	0.84	7.0 x 10^{4}	82.25

**Eastern sub-Saharan Africa**	**0.025**	-0.14	0.03	0.24	-0.18	3.83	0.01	0.65	1.4 x 10^2	1.1 x 10^3
	**0.50**	-0.01	0.34	0.55	0.11	9.96	0.04	0.92	1.3 x 10^4	1.3 x 10^4
	**0.975**	0.12	0.65	0.86	0.39	24.67	0.12	0.99	6.8 x10^4	6.9 x 10^4
**Tropical South America**	**0.025**	-0.91	-0.02	0.33	0.39	2.85	0.27	0.74	3.65	0.34
	**0.50**	-0.37	0.07	0.43	0.50	3.56	0.33	0.81	4.01	0.62
	**0.975**	0.17	0.16	0.52	0.61	4.31	0.40	0.86	4.44	1.27
**East Asia**	**0.025**	-0.61	-0.03	-0.11	0.57	7.62	0.33	0.57	10.72	1.32
	**0.50**	-0.17	0.09	0.11	0.79	10.02	0.46	0.78	14.72	3.69
	**0.975**	0.26	0.21	0.33	1.02	13.18	0.64	0.88	20.49	9.89
**Southeast Asia and Oceania**	**0.025**	-0.95	-0.10	0.30	0.29	4.81	0.55	0.47	1.84	2.89
	**0.50**	-0.63	0.04	0.49	0.47	5.62	0.69	0.78	1.93	7.99
	**0.975**	-0.31	0.18	0.67	0.66	6.67	0.84	0.87	2.01	18.87
**South and Central Asia**	**0.025**	-0.49	0.00	0.37	0.20	4.22	0.17	0.62	5.42	1.86
	**0.50**	-0.17	0.13	0.51	0.36	5.67	0.24	0.78	6.60	5.36
	**0.975**	0.15	0.27	0.65	0.51	7.71	0.34	0.87	8.44	14.70

Supplementary Table 10: Wasting fitted parameters.

Lower, median, and upper quantiles (percentiles 0.025, 0.50, 0.975) are displayed for the main parameters from the wasting models by region. The fixed effects covariates corresponding to the predicted ensemble rasters are shown in the first five columns, while fitted values for the spatiotemporal field hyperparameters and the precisions (inverse variance) for our random effects are shown in the last five columns.

Regions	Percentiles	int	gam	gbm	lasso	Nominal Range	Nominal Variance	AR1 rho	Precision for NUG.ID	Precision for CTRY.ID
**North Africa**	**0.025**	-0.14	0.30	0.41	0.00	2.33	0.18	0.01	1.3x10^3	19.15
	**0.50**	-0.04	0.39	0.50	0.11	2.77	0.22	0.39	1.5 x 10^4	119.64
	**0.975**	0.06	0.47	0.59	0.22	3.35	0.26	0.63	7.0 x 10^4	1662.94
**Central sub-Saharan Africa**	**0.025**	-0.24	-0.34	0.25	0.59	3.36	0.07	0.60	920.87	6.53
	**0.50**	-0.09	-0.19	0.41	0.77	4.41	0.10	0.77	1.2 x 10^4	16.79
	**0.975**	0.06	-0.03	0.58	0.96	5.91	0.14	0.87	6.3 x 10^4	41.49
**Middle East**	**0.025**	-0.25	0.05	0.28	0.17	2.08	0.13	0.18	11.44	3.77
	**0.50**	-0.01	0.20	0.45	0.35	3.09	0.18	0.58	14.28	14.62

	**0.975**	0.23	0.34	0.62	0.54	4.50	0.27	0.79	18.42	50.22
**Central America and the Caribbean**	**0.025**	-0.24	0.07	0.19	0.12	2.08	0.08	0.61	4.78	1053.77
	**0.50**	-0.10	0.28	0.40	0.31	4.00	0.13	0.83	6.72	1.2 x 10^4
	**0.975**	0.05	0.50	0.62	0.50	6.66	0.26	0.92	10.65	6.5 x 10^4
**Western sub-Saharan Africa**	**0.025**	-0.18	-0.11	0.37	0.38	2.68	0.07	0.65	17.22	13.16
	**0.50**	-0.08	-0.01	0.49	0.52	3.24	0.09	0.75	19.13	29.21
	**0.975**	0.02	0.10	0.60	0.66	3.82	0.10	0.82	21.62	61.46
**Andean South America**	**0.025**	-0.02	0.00	0.17	0.34	6.23	0.06	0.64	1.3 x 10^3	2.73
	**0.50**	0.32	0.15	0.34	0.51	12.38	0.13	0.88	1.3 x 10^4	10.56
	**0.975**	0.67	0.30	0.50	0.68	22.30	0.27	0.96	6.7 x 10^4	55.95
**Southern sub-Saharan Africa**	**0.025**	-0.11	-0.13	0.14	0.37	3.21	0.07	0.58	1.4 x 10^3	1093.99
	**0.50**	0.04	0.06	0.35	0.59	5.31	0.12	0.80	1.5 x 10^4	1.3 x 10^4
	**0.975**	0.18	0.26	0.56	0.81	8.44	0.20	0.91	7.1 x 10^4	6.8 x 10^4
**Eastern sub-Saharan Africa**	**0.025**	-0.64	-0.25	0.49	-0.06	21.80	0.04	0.48	1.4 x 10^3	1270.93
	**0.50**	-0.13	0.03	0.74	0.23	41.83	0.10	0.84	1.4 x 10^3	1.3 x 10^4
	**0.975**	0.38	0.30	0.99	0.53	79.12	0.30	0.96	6.7 x 10^4	6.7 x 10^4
**Tropical South America**	**0.025**	-0.20	-0.04	0.29	0.40	2.17	0.10	0.77	6.75	4.34
	**0.50**	-0.05	0.08	0.39	0.53	2.72	0.13	0.84	7.46	9.29
	**0.975**	0.10	0.20	0.50	0.65	3.31	0.15	0.89	8.22	17.88
**East Asia**	**0.025**	-0.50	-0.06	0.37	0.32	4.78	0.26	0.67	54.30	3.68
	**0.50**	-0.22	0.05	0.50	0.45	6.57	0.35	0.82	106.31	11.74
	**0.975**	0.06	0.17	0.63	0.57	9.08	0.51	0.90	229.03	34.39
**Southeast Asia and Oceania**	**0.025**	-0.61	-0.08	0.61	0.16	3.20	0.12	0.80	6.76	4.71
	**0.50**	-0.43	0.01	0.71	0.28	4.08	0.15	0.86	7.24	15.39
	**0.975**	-0.25	0.11	0.81	0.39	4.92	0.20	0.91	7.70	44.37
**South and Central Asia**	**0.025**	-0.07	0.18	0.57	-0.11	1.96	0.08	0.13	84.31	37.53
	**0.50**	0.01	0.29	0.69	0.03	2.51	0.10	0.51	115.59	222.74
	**0.975**	0.09	0.39	0.80	0.17	3.19	0.12	0.71	157.61	2118.83

Supplementary Table 11: Change of overweight and wasting from 2000 to 2017 at the first administrative level in all LMICs.

Country	Wasting increased, Overweight decreased	Wasting increased, Overweight increased	Wasting decreased, overweight decreased	Wasting decreased, overweight increased	Total number of units
Afghanistan	1	0	20	13	34
Algeria	0	0	5	43	48
Angola	0	0	6	12	18
Bangladesh	0	1	0	6	7
Belize	0	0	2	4	6
Benin	0	6	0	6	12
Bhutan	0	7	2	11	20
Bolivia	0	0	6	3	9
Botswana	0	0	0	16	16
Brazil	0	0	2	25	27
Burkina Faso	0	0	0	13	13
Burundi	0	2	1	14	17
Cambodia	0	0	4	21	25
Cameroon	0	0	7	3	10
Cape Verde	0	0	0	22	22
Central African Republic	1	0	16	0	17
Chad	2	4	8	9	23
China	0	0	13	20	33
Colombia	0	0	15	16	32
Comoros	0	0	3	0	3
Costa Rica	0	0	0	7	7
Cuba	0	0	0	16	16
Côte d'Ivoire	0	0	12	2	14
Democratic Republic of the Congo	0	0	7	19	26
Djibouti	0	0	0	5	5
Dominican Republic	0	1	4	27	32
Ecuador	0	0	0	24	24
Egypt	1	6	11	9	27
El Salvador	0	2	2	10	14
Equatorial Guinea	0	0	0	6	7
Eritrea	0	1	0	5	6
Ethiopia	0	0	0	11	11
French Guiana	0	0	0	2	2
Gabon	0	1	0	8	9
Gambia	0	3	1	2	6
Ghana	0	0	2	8	10
Guatemala	0	0	8	14	22
Guinea	0	0	5	3	8
Guinea-Bissau	0	0	6	3	9
Guyana	0	0	0	10	10
Haiti	0	0	2	8	10
Honduras	0	0	3	15	18
India	3	3	12	13	31
Indonesia	2	3	13	16	34

Iran	0	0	1	30	31
Iraq	0	2	0	16	18
Jamaica	0	0	0	14	14
Jordan	0	0	0	12	12
Kenya	0	0	19	28	47
Kyrgyzstan	0	0	5	4	9
Laos	0	7	0	11	18
Lesotho	0	0	10	0	10
Liberia	0	0	0	15	15
Libya	14	1	4	3	22
Madagascar	0	3	0	3	6
Malawi	0	0	3	0	3
Malaysia	2	9	0	4	16
Mali	0	1	3	5	9
Mauritania	0	1	8	4	13
Mexico	0	0	6	26	32
Mongolia	0	0	8	14	22
Morocco	0	0	0	15	15
Mozambique	0	0	0	11	11
Myanmar	0	0	4	11	15
Namibia	2	2	1	8	13
Nepal	0	0	0	5	5
Nicaragua	0	1	4	13	18
Niger	1	1	0	6	8
Nigeria	1	2	15	19	37
Pakistan	5	0	0	2	7
Palestine	0	0	1	1	2
Panama	0	0	0	13	13
Papua New Guinea	0	0	0	22	22
Paraguay	2	0	0	16	18
Peru	0	0	14	12	26
Philippines	0	0	0	81	82
Republic of Congo	8	4	0	0	12
Rwanda	0	0	0	5	5
Senegal	2	0	12	0	14
Sierra Leone	0	0	0	4	4
Somalia	0	1	9	8	18
South Africa	0	1	1	7	9
South Sudan	0	0	0	10	10
Sri Lanka	0	0	1	24	25
Sudan	0	0	9	9	18
Suriname	0	0	1	9	10
Swaziland	0	0	2	2	4
Syria	7	7	0	0	14
São Tomé and Príncipe	0	0	2	0	2
Tajikistan	1	4	0	0	5
Tanzania	0	2	1	27	30
Thailand	4	62	0	11	77
Timor-Leste	0	0	1	12	13
Togo	1	4	0	0	5

Trinidad and Tobago	0	0	0	13	15
Tunisia	0	0	0	24	24
Turkmenistan	0	0	2	4	6
Uganda	0	2	0	56	58
Uzbekistan	0	3	5	6	14
Venezuela	5	11	1	7	25
Vietnam	0	0	0	63	63
Western Sahara	2	0	2	0	4
Yemen	3	10	4	4	21
Zambia	0	6	0	4	10
Zimbabwe	0	0	10	0	10

Supplementary Table 12: Change of overweight and wasting from 2000 to 2017 at the second administrative level in all LMICs.

Country	Wasting increased, Overweight decreased	Wasting increased, Overweight increased	Wasting decreased, overweight decreased	Wasting decreased, overweight increased	Total number of units
Afghanistan	3	9	182	134	328
Algeria	0	0	102	1400	1504
Angola	0	0	56	106	163
Bangladesh	2	13	4	41	60
Belize	0	0	2	4	6
Benin	1	42	2	31	76
Bhutan	1	63	27	114	205
Bolivia	0	0	73	22	95
Botswana	0	0	0	30	30
Brazil	1	268	135	5096	5504
Burkina Faso	0	0	1	44	45
Burundi	0	8	21	104	133
Cambodia	0	0	33	145	178
Cameroon	0	0	38	20	58
Cape Verde	0	0	0	22	22
Central African Republic	7	1	41	2	51
Chad	7	13	14	21	55
China	1	5	132	223	364
Colombia	0	0	726	336	1065
Comoros	0	0	3	0	3
Costa Rica	0	0	0	81	81
Cuba	0	0	0	167	168
Côte d'Ivoire	0	0	27	6	33
Democratic Republic of the Congo	0	5	72	162	239
Djibouti	0	0	0	11	11

Dominican Republic	0	13	18	124	155
Ecuador	0	0	8	215	223
Egypt	29	91	83	134	343
El Salvador	0	22	30	214	266
Equatorial Guinea	0	0	0	29	32
Eritrea	0	2	1	43	46
Ethiopia	0	5	0	74	79
French Guiana	0	0	0	0	378
Gabon	0	6	3	28	37
Gambia	1	14	9	12	37
Ghana	0	0	43	94	137
Guatemala	0	0	143	211	354
Guinea	0	0	17	17	34
Guinea-Bissau	0	1	26	10	37
Guyana	0	0	0	111	116
Haiti	0	0	6	35	41
Honduras	0	0	39	259	298
India	106	61	166	388	722
Indonesia	40	44	176	240	502
Iran	0	2	20	366	388
Iraq	0	17	7	78	102
Jamaica	0	0	0	14	14
Jordan	0	2	1	49	52
Kenya	0	2	127	169	299
Kyrgyzstan	3	0	24	17	44
Laos	0	56	0	86	142
Lesotho	0	0	10	0	10
Liberia	0	0	0	66	66
Libya	14	1	4	3	22
Madagascar	1	6	0	15	22
Malawi	0	0	24	4	28
Malaysia	7	105	0	31	144
Mali	0	3	17	30	50
Mauritania	2	1	29	12	44
Mexico	0	0	353	2101	2454
Mongolia	1	1	128	197	327
Morocco	0	0	1	53	54
Mozambique	0	0	11	118	129
Myanmar	0	0	15	48	63
Namibia	7	31	5	64	107
Nepal	0	0	0	14	14

Nicaragua	0	19	23	97	139
Niger	7	3	0	26	36
Nigeria	26	37	330	382	775
Pakistan	19	8	2	3	32
Palestine	0	0	5	11	16
Panama	0	0	7	72	79
Papua New Guinea	0	0	0	87	87
Paraguay	13	0	4	201	218
Peru	0	0	97	98	195
Philippines	0	27	37	1562	1647
Republic of Congo	37	10	1	0	48
Rwanda	0	0	0	30	30
Senegal	7	1	33	4	45
Sierra Leone	0	0	0	14	14
Somalia	2	2	32	38	74
South Africa	0	3	10	39	52
South Sudan	0	0	0	45	45
Sri Lanka	0	0	7	315	323
Sudan	1	0	32	47	80
Suriname	0	0	2	60	62
Swaziland	0	0	29	26	55
Syria	30	30	0	0	60
São Tomé and Príncipe	0	0	6	0	7
Tajikistan	31	23	0	5	59
Tanzania	1	17	6	158	183
Thailand	32	776	0	120	928
Timor-Leste	0	0	4	58	62
Togo	5	13	0	3	21
Trinidad and Tobago	0	0	0	13	15
Tunisia	0	0	5	256	268
Turkmenistan	0	0	2	4	6
Uganda	1	3	0	79	83
Uzbekistan	8	30	56	67	161
Venezuela	87	159	23	65	338
Vietnam	0	0	9	698	710
Western Sahara	2	0	2	0	4
Yemen	44	147	95	43	333
Zambia	0	35	0	37	72
Zimbabwe	0	0	59	1	60

4.0 Model Validation

4.1 Overweight validation metrics

Supplementary Table 13: Predictive metrics for overweight aggregated to admin 0. The out-of-sample (OOS) column indicates whether the metric was calculated using in-sample or out-of-sample predictions. The models included 420 geo-referenced household surveys representing over 3 million under-5 children. Pearson correlation method was used to compute correlation values.

Year	OOS	Median SS	Mean Err.	RMSE	Corr.	95% Cov.
**2000**	FALSE	1798.500	-0.013	0.036	0.918	0.942
**2005**	FALSE	2022.000	-0.003	0.022	0.950	0.902
**2010**	FALSE	3177.339	-0.006	0.018	0.969	0.923
**2015**	FALSE	2387.885	-0.015	0.021	0.971	0.958
**2000**	TRUE	1798.500	-0.003	0.033	0.903	0.906
**2005**	TRUE	2022.000	0.000	0.021	0.944	0.889
**2010**	TRUE	3177.339	-0.003	0.019	0.964	0.912
**2015**	TRUE	2387.885	-0.015	0.021	0.956	0.952

Supplementary Table 14: Predictive metrics for overweight aggregated to admin 1. The out-of-sample (OOS) column indicates whether the metric was calculated using in-sample or out-of-sample predictions. The models included 420 geo-referenced household surveys representing over 3 million under-5 children. Pearson correlation method was used to compute correlation values.

Year	OOS	Median SS	Mean Err.	RMSE	Corr.	95% Cov.
**2000**	FALSE	211.609	0.001	0.041	0.936	0.977
**2005**	FALSE	308.102	-0.015	0.041	0.941	0.942
**2010**	FALSE	337.062	-0.014	0.046	0.923	0.940
**2015**	FALSE	377.000	-0.020	0.045	0.926	0.955
**2000**	TRUE	211.609	-0.003	0.057	0.881	0.965
**2005**	TRUE	308.102	-0.019	0.052	0.906	0.931
**2010**	TRUE	337.062	-0.020	0.057	0.885	0.922
**2015**	TRUE	377.000	-0.023	0.051	0.902	0.947

Supplementary Table 15: Predictive metrics for overweight aggregated to admin 2. The out-of-sample (OOS) column indicates whether the metric was calculated using in-sample or out-of-sample predictions. The models included 420 geo-referenced household surveys representing over 3 million under-5 children. Pearson correlation method was used to compute correlation values.

Year	OOS	Median SS	Mean Err.	RMSE	Corr.	95% Cov.
**2000**	FALSE	27.693	0.001	0.055	0.897	0.977
**2005**	FALSE	33.059	-0.015	0.059	0.885	0.941

**2010**	FALSE	41.054	-0.014	0.067	0.861	0.940
**2015**	FALSE	36.000	-0.020	0.074	0.827	0.954
**2000**	TRUE	27.693	-0.003	0.071	0.828	0.964
**2005**	TRUE	33.059	-0.019	0.072	0.834	0.931
**2010**	TRUE	41.054	-0.020	0.080	0.801	0.922
**2015**	TRUE	36.000	-0.023	0.083	0.776	0.947

4.2 Wasting validation metrics

Supplementary Table 16: Predictive metrics for wasting aggregated to admin 0.
The out-of-sample (OOS) column indicates whether the metric was calculated using in-sample or out-of-sample predictions. The models included 420 geo-referenced household surveys representing over 3 million under-5 children. Pearson correlation method was used to compute correlation values.

Year	OOS	Median SS	Mean Err.	RMSE	Corr.	95% Cov.
**2000**	FALSE	1775.442	-0.002	0.009	0.989	0.982
**2005**	FALSE	2022.000	0.000	0.013	0.983	0.938
**2010**	FALSE	3180.421	-0.003	0.011	0.980	0.967
**2015**	FALSE	2409.500	-0.001	0.009	0.990	0.973
**2000**	TRUE	1775.442	-0.003	0.028	0.862	0.941
**2005**	TRUE	2022.000	-0.008	0.026	0.948	0.908
**2010**	TRUE	3180.421	-0.011	0.028	0.898	0.926
**2015**	TRUE	2409.500	0.001	0.023	0.919	0.959

Supplementary Table 17: Predictive metrics for wasting aggregated to admin 1.
The out-of-sample (OOS) column indicates whether the metric was calculated using in-sample or out-of-sample predictions. The models included 420 geo-referenced household surveys representing over 3 million under-5 children. Pearson correlation method was used to compute correlation values.

Year	OOS	Median SS	Mean Err.	RMSE	Corr.	95% Cov.
**2000**	FALSE	96.000	-0.002	0.028	0.905	0.982
**2005**	FALSE	134.000	0.000	0.029	0.934	0.939
**2010**	FALSE	146.579	-0.003	0.030	0.902	0.966
**2015**	FALSE	181.000	-0.001	0.033	0.913	0.973
**2000**	TRUE	96.000	-0.002	0.046	0.735	0.937
**2005**	TRUE	134.000	-0.008	0.043	0.873	0.908
**2010**	TRUE	146.579	-0.011	0.043	0.803	0.924
**2015**	TRUE	181.000	0.001	0.052	0.749	0.960

Supplementary Table 18: Predictive metrics for wasting aggregated to admin 2.

The out-of-sample (OOS) column indicates whether the metric was calculated using in-sample or out-of-sample predictions. The models included 420 geo-referenced household surveys representing over 3 million under-5 children. Pearson correlation method was used to compute correlation values.

Year	OOS	Median SS	Mean Err.	RMSE	Corr.	95% Cov.
**2000**	FALSE	12.000	-0.002	0.042	0.817	0.982
**2005**	FALSE	14.047	0.000	0.048	0.851	0.938
**2010**	FALSE	17.453	-0.003	0.052	0.774	0.965
**2015**	FALSE	16.000	-0.001	0.065	0.747	0.973
**2000**	TRUE	12.000	-0.002	0.059	0.636	0.936
**2005**	TRUE	14.047	-0.008	0.060	0.777	0.907
**2010**	TRUE	17.447	-0.011	0.062	0.674	0.924
**2015**	TRUE	16.000	0.001	0.078	0.599	0.959

5.0 Supplementary References

1. Stevens, G. A. *et al.* Guidelines for Accurate and Transparent Health Estimates Reporting: the GATHER statement. *PLOS Med.* **13**, e1002056 (2016).
2. Dicker, D. *et al.* Global, regional, and national age-sex-specific mortality and life expectancy, 1950–2017: a systematic analysis for the Global Burden of Disease Study 2017. *Lancet* **392**, 1684–1735 (2018).

6.0 Author Contributions

Managing the estimation or publications process

Damaris K Kinyoki, Sandra B Munro, Laurie B Marczak, Megan F Schipp, Ashkan Afshin, and Simon I Hay

Writing the first draft of the manuscript

Damaris K Kinyoki, Jennifer M Ross, Sandra B Munro, Lauren E Schaeffer, Megan F Schipp, Ashkan Afshin, and Simon I Hay

Primary responsibility for this manuscript focused on: applying analytical methods to produce estimates

Damaris K Kinyoki, Aaron E Osgood-Zimmerman, Jonathan F Mosser, Aniruddha Deshpande

Primary responsibility for this manuscript focused on: seeking, cataloguing, extracting, or cleaning data; designing or coding figures and tables

Damaris K Kinyoki, Alice Lazzar-Atwood, Brandon V Pickering, Ian D Letourneau

Providing data or critical feedback on data sources

Hedayat Abbastabar, Ahmed Abdelalim, Amir Abdoli, Kanaan Abdullah, Hassan Abolhassani, Michael RM Abrigo, Manfred Accrombessi, Maryam Adabi, Daniel Adane, Akindele Adebiyi, Isaac Adedeji, Victor Adekanmbi, Olatunji Adetokunboh, Davoud Adham, Shailesh Advani, Mohsen Afarideh, Mohammad Aghaali, Tauseef Ahmad, Muktar Ahmed, Turki Alanzi, Jacqueline E Alcalde-Rabanal, Niguse Alema, Muhammad Ali, Beriwan Ali, Beriwan Ali, Saqib Ali, Cyrus Alinia, Vahid Alipour, Hesam Alizade, Syed Aljunid, Amir Almasi-Hashiani, Nelson Alvis-Guzman, Nelson J Alvis-Zakzuk, Azmeraw T Amare, Saeed Amini, Nahla Anber, Fereshteh Ansari, Carl Abelardo Antonio, Davood Anvari, Razique Anwer, Jalal Arabloo, Zohreh Arefi, Olatunde Aremu, Samaneh Asgari, Ashish Awasthi, Beatriz Paulina Ayala Quintanilla, Samad Azari, Ghasem Azarian, Ashish Badiye, Mohammad Amin Bahrami, Senthilkumar Balakrishnan, Maciej Banach, Palash Chandra Banik, Miguel A Barboza, Till Bärnighausen, Sanjay Basu, Dereje Bayissa, Kidanemaryam Berhe, Adam Berman, Ali Bijani, Boris Bikbov, Minyichil Birhanu, Somayeh Bohlouli, Mahdi Bohluli, Srinivasa Bolla, Nicola Luigi Bragazzi, Nikolay Briko, Alessandra C Goulart, Juan J Carrero, Deborah Carvalho Malta, Carlos Castañeda-Orjuela, Franz Castro, Vijay Kumar Chattu, Bal Govind Chauhan, Bal Govind Chauhan, Dinh Toi Chu, Abel Dadi, Giovanni Damiani, Aso Darweesh, Amira Hamed Darwish, Ahmad Daryani, Desta Debalkie Atnafu, Asmamaw Demis, Dereje Demissie, Gebre Demoz, Nebiyu Dereje, Kebede Deribe, Rupak Desai, Assefa Desalew, Preeti Dhillon, Meghnath Dhimal, Govinda Dhungana, Mostafa Dianatinasab, Girmaye Dinsa, Hoa Do Thi, Manisha Dubey, Bereket Duko, Susanna Dunachie, Aziz Eftekhari, Iman El Sayed, Varavikova Elena, Ziad El-Khatib, Iqbal Elyazar, Mohammad Hassan Emamian, Shymaa Enany, Tewodros Eshete, Khalil Eskandari, Saman Esmaeilnejad, Alireza Esteghamati, Atkilt Esaiyas Etisso, Anwar Faraj, Sajjad Farashi, Mohammad Fareed, Andrea Farioli, Andre Faro, Farshad Farzadfar, Nelsensius Klau Fauk, Seyed-Mohammad Fereshtehnejad, Joao Fernandes, Irina Filip, Carsten Flohr, Nataliya Foigt, Morenike Folayan, Artem Fomenkov, Artem Fomenkov, Takeshi Fukumoto, Ketema Bizuwork Gebremedhin, Gebretsadkan Gebretsadik, Getnet Gedefaw, Hailay Gesesew, Mansour Ghafourifard, Mansour Ghafourifard, Alireza Ghajar, Ahmad Ghashghaee, Ibrahim Ginawi, Yordanos Gizachew, Srinivas Goli, Nelson Gomes, Ayman Grada, Michal Grivna, Rajat Das Gupta, Nima Hafezi-Nejad, Michael Tamene Haile, Arvin Haj-Mirzaian, Arya Haj-Mirzaian, Hamidreza Haririan, Ninuk Hariyani, Edris Hasanpoor, Amir Hasanzadeh, Amir Hasanzadeh, Soheil Hassanipour, Hamid Y Hassen, Reza Heidari-Soureshjani, Nathaniel Henry, Mario Herrero, Hagos Hidru, Chi Hoang Linh, Hans Hoek, Praveen Hoogar, Mehdi Hosseinzadeh, Mohamed Hsairi, Guoqing Hu, Segun Emmanuel Ibitoye, Usman Iqbal, Sheikh Mohammed Shariful Islam, Nader Jafaribalalami, Morteza Jafarinia, Mihajlo Jakovljevic, Ahamarshan Jayaraman Nagarajan, Achala Jayatilleke, Panniyammakal Jeemon, John Ji, Peng Jia, Yetunde O John-Akinola, Kimberly Johnson, Jost B Jonas, Farahnaz Joukar, Jacek Jozwiak, Mikk Jürisson, Molla Kahssay, Leila R Kalankesh, Rohollah Kalhor, Tanuj Kanchan, Umesh Kapil, umesh kapil, Neeti Kapoor, Manoochehr Karami, André Karch, Surendra Karki, Amir Kasaeian, Habtamu Kasaye, Habtamu Kasaye, Hagazi Kassaye, Nicholas Kassebaum, Yousef Khader, Morteza Abdullatif Khafaie, Nauman Khalid, Ibrahim Khalil, Rovshan Khalilov, Md Nuruzzaman Khan, Muhammad Shahzeb Khan, Khaled Khatab, Amir khater, Milad Khorasani, Daniel Kiirithio, Yun Jin Kim, Young-Eun Kim, Adnan Kisa, Sezer Kisa, Hamidreza Komaki, Parvaiz Koul, Kewal Krishan, Kris Krohn, Pushpendra Kumar, Dharmesh Lal, Huong Lan

Nguyen, Savita Lasrado, Mostafa Leili, Chi Linh Hoang, Shiwei Liu, Joshua Longbottom, Jaifred Christian Lopez, Dhaval Maghavani, Narayan Mahotra, Navid Manafi, Ali Manafi, Farzad Manafi, Fariborz Mansour-Ghanaei, Borhan Mansouri, Mohammad Ali Mansournia, Francisco Rogerlândio Martins-Melo, Benjamin Mayala, Mohsen Mazidi, Man Mohan Mehndiratta, Fereshteh Mehri, Hagazi Gebre Meles, Addisu Melese, Walter Mendoza, Ritesh G Menezes, Tuomo Meretoja, Ted R Miller, Biniam Minuye, Hamed Mirjalali, Erkin Mirrakhimov, Prasanna Mithra, Babak Moazen, Amjad Mohamadi Bolbanabad, Karzan Mohammad, Karzan Mohammad, Shadieh Mohammadi, Reza Mohammadpourhodki, Shafiu Mohammed, Ammas Mohammed, Farnam Mohebi, Ali Mokdad, Yoshan Moodley, Maryam Moossavi, Masoud Moradi, Ghobad Moradi, Mohammad Moradi-Joo, Maziar Moradi-Lakeh, Abbas Mosapour, Simin Mouodi, Amin Mousavi Khaneghah, Ulrich Mueller, Kamarul Imran Musa, Ghulam Mustafa, Saravanan Muthupandian, Girish Nadkarni, Mohsen Naghavi, Seyed Sina Naghibi Irvani, Vinod C Nayak, Ionut Negoi, Ruxandra Irina Negoi, Josephine Ngunjiri, Dabere Nigatu, Yeshambel Nigatu, Rajan Nikbakhsh, Chukwudi Nnaji, Jean Jacques Noubiap, Christoph Nowak, Olanrewaju Oladimeji, Andrew T Olagunju, Tinuke O Olagunju, Bolajoko Olusanya, Jacob Olusanya, Ahmed Omar Bali, Obinna Onwujekwe, Alberto Ortiz, Mayowa Owolabi, Mahesh P A, Keyvan Pakshir, Songhomitra Panda-Jonas, Sangram Kishor Patel, Mona Pathak, Ajay Patle, Hamidreza Pazoki-Toroudi, Veincent Christian Pepito, William Petri, Huyen Phuc Do, David Pigott, Bakhtiar Piroozi, Maarten Postma, Hadi Pourjafar, Akram Pourshams, Hossein Poustchi, Sergio Prada, Dimas Ria Angga Pribadi, Amir Radfar, Alireza Rafiei, Fakher Rahim, Vafa Rahimi-Movaghar, Sajjad Rahman, Ali Rajabpour-Sanati, Kiana Ramezanzadeh, Saleem Rana, Chhabi Lal Ranabhat, Priya Rathi, Salman Rawaf, David Rawaf, Lal Rawal, Reza Rawassizadeh, Vishnu Renjith, Andre Renzaho, Bhageerathy Reshmi, Nima Rezaei, Ana Isabel Ribeiro, Leonardo Roever, Gholamreza Roshandel, Ali Rostami, Sadegh Rostamnia, Enrico Rubagotti, Seyedmohammad Saadatagah, Ehsan Sadeghi, Yahya Safari, Hamid Safarpour, S Mohammad Sajadi, Mohammad Reza Salahshoor, Nasir Salam, Marwa Salem, Hosni Salem, Evanson Z Sambala, Abdallah M Samy, Bruno Sao Jose, Sivan Saraswathy, Abdur Razzaque Sarker, Benn Sartorius, Arash Sarveazad, Brijesh Sathian, Maheswar Satpathy, David C Schwebel, Seyedmojtaba Seyedmousavi, Faramarz Shaahmadi, Masood Ali Shaikh, Morteza Shamsizadeh, Mohd Shannawaz, Mehdi Sharif, Wondimeneh Shiferaw, Rahman Shiri, Soraya Siabani, Tariq Jamal Siddiqi, Jasvinder Singh, Virendra Singh, Amin Soheili, Farzaneh Soltani, JB Soriano, Sergey Soshnikov, Chandrashekhar T Sreeramareddy, Antonina Starodubova, Antonina Starodubova, Agus Sudaryanto, Hafiz Ansar Rasul Suleria, Bryan L Sykes, Rafael Tabarés-Seisdedos, Birkneh Tilahun Tadesse, Amir Taherkhani, Cuong Tat Nguyen, Nuno Taveira, Girmay Teklay, Michael Temene, Maria Titova, Maria Titova, Marcos Roberto Tovani-Palone, Eugenio Traini, Bach Tran, Reta Tsegaye, Irfan Ullah, Bhaskaran Unnikrishnan, Era Upadhyay, Ushotanefe Useh, Muhammad Shariq Usman, Olalekan Uthman, Masoud Vaezghasemi, John VanderHeide, Tommi Vasankari, Yasser Vasseghian, Srinivasaraghavan Venkatesh, Narayanaswamy Venketasubramanian, Madhur Verma, Vasiliy Vlassov, Rade Vukovic, Yasir Waheed, Taweewat Wiangkham, Tissa Wijeratne, Haileab Fekadu Wolde, Dawit Zewdu Wondafrash, Gelin Xu, Abbas Yadegar, Ali Yadollahpour, Tomohide Yamada, Sanni Yaya, Alex Yeshaneh, Mekdes Yilma, Mekdes Yilma, Tesfaye Yitna, Naohiro Yonemoto, Seok-Jun Yoon, Zabihollah Yousefi, Chuanhua Yu, Mohammad Zamani, Hadi Zarafshan, Nejimu Zepro, Xiu-Ju Zhao, Arash Ziapour

Development of methods or computational machinery

Kanaan Abdullah, Isaac Adedeji, Davoud Adham, Muktar Ahmed, Saqib Ali, Hesam Alizade, Nahla Anber, Fereshteh Ansari, Zahra Bagheri-Hosseinabadi, Masoud Behzadifar, Meysam Behzadifar, Mahdi Bohluli, Michael Collison, Michael Cork, Giovanni Damiani, Aso Darweesh, Lucas Earl, Aziz Eftekhari, Anwar Faraj, Ali Akbar Fazaeli, Carsten Flohr, Rajat Das Gupta, Yadollah Hamidi, Amir Hasanzadeh, Nathaniel Henry, Mario Herrero, Mehdi Hosseinzadeh, Morteza Jafarinia, Jacek Jozwiak, Nicholas Kassebaum, Rovshan Khalilov, Mohammad Taghi Khodayari, Mohammad Hossein Khosravi, Adnan Kisa, Sezer Kisa, Kris Krohn, Georgy Lebedev, Jaifred Christian Lopez, Navid Manafi, Ali Manafi, Farzad Manafi, Borhan Mansouri, Dadi Marami, Mohsen Mazidi, Shadieh Mohammadi, Reza Mohammadpourhodki, Ali Mokdad, Masoud Moradi, Rahmatollah Moradzadeh, Jonathan Mosser, Zelalem Nigussie, Bogdan Oancea, Ahmed Omar Bali, Aaron Osgood-Zimmerman, David Pigott, Hadi Pourjafar, Dimas Ria Angga Pribadi, Ali Rajabpour-Sanati, Chhabi Lal Ranabhat, Priya Rathi, Reza Rawassizadeh, Vishnu Renjith, Seyed Mohammad Riahi, Yahya Safari, Abdallah M Samy, Maheswar Satpathy, Alyssa Sbarra, Faramarz Shaahmadi, Farzaneh Soltani, Mojtaba Soltani, John VanderHeide, Yasser Vasseghian, Sanni Yaya, Vahid Yazdi-Feyzabadi, Maryam Zamanian, Mojtaba Ziaee, Arash Ziapour

Providing critical feedback on methods or results

Jennifer Ross, Sandra Munro, Lauren Schaeffer, Masoumeh Abbasi, Hedayat Abbastabar, Ahmed Abdelalim, Mohammad Abdollahi, Ibrahim Abdollahpour, Kanaan Abdullah, Teshome Abuka Abebo, Lucas Guimarães Abreu, Michael RM Abrigo, Anemaw Asrat Achamyeleh, Dilaram Acharya, Maryam Adabi, Isaac Adedeji, Victor Adekanmbi, Abiodun Adeoye, Olatunji Adetokunboh, Davoud Adham, Pana Adrian, Posi Aduroja, Shailesh Advani, Mohsen Afarideh, Mohammad Aghaali, Anurag Agrawal, Tauseef Ahmad, Keivan Ahmadi, Sepideh Ahmadi, Muktar Ahmed, Olufemi Ajumobi, Temesgen Yihunie Akalu, Tomi Akinyemiju, Ziyad Al-Aly, Genet Melak Alamene, Jacqueline E Alcalde-Rabanal, Muktar Ali, Saqib Ali, Mehran Alijanzadeh, Cyrus Alinia, Vahid Alipour, Hesam Alizade, Syed Aljunid, Afshin Almasi, Amir Almasi-Hashiani, Hesham Al-Mekhlafi, Rajaa Al-Raddadi, Khalid Altirkawi, Nelson Alvis-Guzman, Nelson J Alvis-Zakzuk, Azmeraw T Amare, Saeed Amini, Mostafa Amini-Rarani, Arianna Maever Amit, Nahla Anber, Robert Ancuceanu, Catalina Liliana Andrei, Fereshteh Ansari, Alireza Ansari-Moghaddam, Zelalem Alamrew Anteneh, Ernoiz Antriyandarti, Davood Anvari, Razique Anwer, Seth Christopher Yaw Appiah, Jalal Arabloo, Morteza Arab-Zozani, Ephrem Mebrahtu Araya, Zohreh Arefi, Olatunde Aremu, Johan Ärnlöv, Ali Asadi-Pooya, Bahar Ataeinia, Hagos Tasrw Atalay, Marcel Ausloos, Euripide Avokpaho, Ashish Awasthi, Beatriz Paulina Ayala Quintanilla, Assefa Ayalew, Martin Ayanore, Yared Aynalem, Samad Azari, Ebrahim Babaee, Alaa Badawi, Ashish Badiye, Zahra Bagheri-Hosseinabadi, Ahad Bakhtiari, Senthilkumar Balakrishnan, Maciej Banach, Agegnehu Bante, Adhanom Gebreegziabher Baraki, Till Bärnighausen, Lingkan Barua, Sanjay Basu, Mohsen Bayati, Mulat Bayih, Dereje Bayissa, Habtamu Wondifraw Baynes, Neeraj Bedi, Meysam Behzadifar, Yibeltal Alemu Bekele, Derrick Bennett, Kidanemaryam Berhe, Adam Berman, Robert Bernstein, Pankaj Bhardwaj, Krittika Bhattacharyya, Boris Bikbov, Ver Bilano, Muhammad Shahdaat Bin Sayeed, Antonio Biondi, Setognal Birara, Raaj Kishore Biswas, Zebenay Workneh Bitew, Tone Bjørge, Kassawmar Angaw Bogale, Somayeh Bohlouli, Mahdi

Bohluli, Archith Boloor, Shiva Borzouei, Oliver Brady, Nicola Luigi Bragazzi, Gabrielle Britton, Zahid Butt, Luis Alberto Camera, Luis Cámera, Ismael Campos-Nonato, Jorge Cano, Rosario Cárdenas, Félix Carvalho, Deborah Carvalho Malta, João Mauricio Castaldelli-Maia, Carlos Castañeda-Orjuela, Franz Castro, Ester Cerin, Collins Chansa, Jaykaran Charan, Pranab Chatterjee, Vijay Kumar Chattu, Bal Govind Chauhan, Bal Govind Chauhan, Ali Reza Chavshin, Mohammad Chehrazi, Ken Chin, Yilma Chisha, Devasahayam Christopher, Dinh Toi Chu, Flavia Cicuttini, Michael Collison, Michael Cork, Paolo Angelo Cortesi, Vera M Costa, Ewerton Cousin, Elizabeth Cromwell, Baye Dagnew, Saad Dahlawi, Giovanni Damiani, Aso Darweesh, Amira Hamed Darwish, Ahmad Daryani, Jai Das, Claudio Davila, Nicole Davis Weaver, Diego De Leo, Jan-Walter De Neve, Desta Debalkie Atnafu, Asmamaw Demis, Dereje Demissie, Nebiyu Dereje, Kebede Deribe, Rupak Desai, Beruk Desalegn, Sagnik Dey, Preeti Dhillon, Meghnath Dhimal, Govinda Dhungana, Mostafa Dianatinasab, Daniel Diaz, Zahra Sadat Dibaji Forooshani, Girmaye Dinsa, Isaac Oluwafemi Dipeolu, Shirin Djalalinia, Hoa Do Thi, Fariba Dorostkar, Leila Doshmangir, Manisha Dubey, Bereket Duko, Bruce Duncan, Andre Duraes, Lucas Earl, Hamed Ebrahimzadeh Leylabadlo, Aziz Eftekhari, Iman El Sayed, Maysaa El Sayed Zaki, Maha El Tantawi, Demelash Elemineh, Varavikova Elena, Shaimaa El-Jaafary, Ziad El-Khatib, Mohammad Hassan Emamian, Tewodros Eshete, Khalil Eskandari, Sharareh Eskandarieh, Saman Esmaeilnejad, Arash Etemadi, Jessica Fanzo, Mohammad Farahmand, Anwar Faraj, Mohammad Fareed, Roghiyeh Faridnia, Mithila Faruque, Farshad Farzadfar, Hossein Farzam, Alebachew Fasil, Nazir Fattahi, Netsanet Fentahun, Seyed-Mohammad Fereshtehnejad, Joao Fernandes, Eduarda Fernandes, Garumma Tolu Feyissa, Irina Filip, Florian Fischer, Carsten Flohr, Nataliya Foigt, Morenike Folayan, Masoud Foroutan, Jana Förster, Joel Francis, Berhane Fseha, Takeshi Fukumoto, Tsegaye Gebrehiwot, Kidane Tadesse Gebremariam, Hadush Gebremariam, Ketema Bizuwork Gebremedhin, Gebrekiros Gebremichael, Gebretsadkan Gebretsadik, Ayele Geleto, Chalachew Genet, Hailay Gesesew, Kebede Embaye Gezae, Mansour Ghafourifard, Mansour Ghafourifard, Alireza Ghajar, Mahsa Ghajarzadeh, Ahmad Ghashghaee, Fariba Ghassemi, Hesam Ghiasvand, Asadollah Gholamian, Syed Amir Gilani, Srinivas Goli, Nelson Gomes, Sameer Gopalani, Houman Goudarzi, Ayman Grada, Michal Grivna, Rashid A Guled, Yuming Guo, Rajat Das Gupta, Rajeev Gupta, Rahul Gupta, Desta Haftu, Michael Tamene Haile, Arvin Haj-Mirzaian, Arya Haj-Mirzaian, Brian Hall, Iman Halvaei, Randah Hamadeh, Demelash Woldeyohannes Handiso, Ninuk Hariyani, Md Mehedi Hasan, Milad Hasankhani, Edris Hasanpoor, Amir Hasanzadeh, Amir Hasanzadeh, Soheil Hassanipour, Hamid Y Hassen, Corinna Hawkes, Khezar Hayat, Behnam Heidari, Delia Hendrie, Nathaniel Henry, Mario Herrero, Claudiu Herteliu, Fatemeh Heydarpour, Hagos Hidru, Chi Hoang Linh, Michael Hole, Enayatollah Homaie Rad, Sung Hwi Hong, Praveen Hoogar, Masako Horino, Naznin Hossain, Seyyed Nasrollah Hosseini, Mehdi Hosseinzadeh, Guoqing Hu, Tanvir Huda, Ayesha Humayun, Segun Emmanuel Ibitoye, Abdelrahman Ibrahim Abushouk, Olayinka Ilesanmi, Milena Ilic, Usman Iqbal, Sheikh Mohammed Shariful Islam, Chidozie Iwu, Jalil Jaafari, Anelisa Jaca, Farhad Jadidi-Niaragh, Nader Jafaribalalami, Morteza Jafarinia, Mohammad Ali Jahani, Mihajlo Jakovljevic, Amir Jalali, Farzad Jalilian, Farzad Jalilian, Ahamarshan Jayaraman Nagarajan, Panniyammakal Jeemon, Fyezah Jehan, Ensiyeh Jenabi, Ravi Prakash Jha, John Ji, Peng Jia, Oommen John, Yetunde O John-Akinola, Jost B Jonas, Nitin Joseph, Farahnaz Joukar, Jacek Jozwiak, Chinwe Juliana, Suresh Jungari, Mikk Jürisson, Zubair Kabir, Hossein Samadi Kafil, Amaha Kahsay, Molla Kahssay, Hamed Kalani, Leila R Kalankesh, Rohollah Kalhor, Tanuj Kanchan, Rajni Kant, Neeti Kapoor,

Manoochehr Karami, Behzad Karami Matin, Surendra Karki, Amir Kasaeian, Gebremicheal Gebreslassie Kasahun, Habtamu Kasaye, Habtamu Kasaye, Tesfaye Kassa, Nicholas Kassebaum, Ali Kazemi Karyani, Andre P Kengne, Daniel Ketema, Yousef Khader, Morteza Abdullatif Khafaie, Mojtaba Khaksarian, Nauman Khalid, Ibrahim Khalil, Rovshan Khalilov, Ejaz Khan, Asaduzzaman Khan, Muhammad Shahzeb Khan, Khaled Khatab, Mona Khater, Maryam Khayamzadeh, Maryam Khazaee-Pool, Salman Khazaei, Mohammad Hossein Khosravi, Roba Khundkar, Ali Kiadaliri, Neda Kianipour, Daniel Kiirithio, Yun Jin Kim, Young-Eun Kim, Ruth Kimokoti, Adnan Kisa, Sezer Kisa, Tufa Kolola, Hamidreza Komaki, Ali Koolivand, Ai Koyanagi, Kewal Krishan, Kris Krohn, Nuworza Kugbey, Manasi Kumar, Pushpendra Kumar, Om Kurmi, Oluwatosin Kuti, Carlo La Vecchia, Sheetal D Lad, Dharmesh Lal, Aparna Lal, Faris Lami, Prabhat Lamichhane, Huong Lan Nguyen, Justin Lang, Savita Lasrado, Georgy Lebedev, Shaun Lee, Paul Lee, Sonia Lewycka, Shanshan Li, Lee-Ling Lim, Melese Linger, Chi Linh Hoang, Shai Linn, Simin Liu, Rakesh Lodha, Jaifred Christian Lopez, Stefan Lorkowski, Erlyn Rachelle Macarayan, Mohamed Magdy Abdel Razek, Hassan Magdy Abdel Razek, Phetole Mahasha, Venkatesh Maled, Shokofeh Maleki, Navid Manafi, Ali Manafi, Farzad Manafi, Narendar Manohar, Fariborz Mansour-Ghanaei, Borhan Mansouri, Mohammad Ali Mansournia, Christopher Chabila Mapoma, Dadi Marami, Carlos Marrugo, Francisco Rogerlândio Martins-Melo, Anthony Masaka, Pallab K Maulik, Benjamin Mayala, Mohsen Mazidi, Birhanu Geta Meharie, Man Mohan Mehndiratta, Kala M Mehta, Wahengbam Bigyananda Meitei, Teferi Mekonnen, Feleke Mekonnen, Hagazi Gebre Meles, Addisu Melese, Walter Mendoza, Ritesh G Menezes, Meresa Mengesha, Tuomo Meretoja, Tomasz Miazgowski, Kebadnew Mulatu Mihretie, Neda Milevska Kostova, Ted R Miller, Edward Mills, GK Mini, Biniam Minuye, Mohammad Miri, Hamed Mirjalali, Roya Mirzaei, Hamed Mirzaei, Mehdi Mirzaei-Alavijeh, Prasanna Mithra, Babak Moazen, Efat Mohamadi, Amjad Mohamadi Bolbanabad, Dara Mohammad, Naser mohammad gholi mezerji, Shadieh Mohammadi, Abdollah Mohammadian-Hafshejani, Reza Mohammadpourhodki, Shafiu Mohammed, Ammas Mohammed, Yousef Mohammed, Salahuddin Mohammed, Jemal Abdu Mohammed, Farnam Mohebi, Amin Mokari Yamchi, Ali Mokdad, Julio Montañez, Pablo Montero-Zamora, Masoud Moradi, Yousef Moradi, Ghobad Moradi, Mohammad Moradi-Joo, Maziar Moradi-Lakeh, Farhad Moradpour, Rahmatollah Moradzadeh, Paula Moraga, Shane Morrison, Jonathan Mosser, Dariush Mozaffarian, Ulrich Mueller, Christopher J L Murray, GVS Murthy, Kamarul Imran Musa, Ghulam Mustafa, Saravanan Muthupandian, Mehdi Naderi, Mehdi Naderi, Seyed Sina Naghibi Irvani, Aliya Naheed, Gurudatta Naik, Farid Najafi, Jobert Richie Nansseu, K M Venkat Narayan, Vinod C Nayak, Duduzile Ndwandwe, Ionut Negoi, Ruxandra Irina Negoi, Josephine Ngunjiri, jing nie, Dabere Nigatu, Yeshambel Nigatu, Zelalem Nigussie, Dina Nur Anggraini Ningrum, Chukwudi Nnaji, Christoph Nowak, Richard Ofori-Asenso, In-Hwan Oh, Olanrewaju Oladimeji, Andrew T Olagunju, Tinuke O Olagunju, Bolajoko Olusanya, Jacob Olusanya, Mojisola Oluwasanu, Ahmed Omar Bali, Obinna Onwujekwe, Kwaku Oppong Asante, Orish Ebere Orisakwe, Alberto Ortiz, Osayomwanbo Osarenotor, Aaron Osgood-Zimmerman, Mayowa Owolabi, Mahesh P A, Dr Jagadish Rao Padubidri, Adrian Pana, Songhomitra Panda-Jonas, Tahereh Pashaei, Deepak Kumar Pasupula, Sangram Kishor Patel, Mona Pathak, Dr Sanghamitra Pati, George Patton, Kebreab Paulos, Hamidreza Pazoki, Veincent Christian Pepito, Norberto Perico, Huyen Phuc Do, David Pigott, Bakhtiar Piroozi, Meghdad Pirsaheb, Meghdad Pirsaheb, Khem Narayan Pokhrel, Maarten Postma, Hadi Pourjafar, Farshad Pourmalek, Sergio Prada, Liliana Lucia Preotescu, Dimas Ria Angga Pribadi, Navid Rabiee,

Mohammad Rabiee, Amir Radfar, Alireza Rafiei, Fakher Rahim, Vafa Rahimi-Movaghar, Muhammad Aziz Rahman, Rajesh Kumar Rai, Ali Rajabpour-Sanati, Fatemeh Rajati, Kiana Ramezanzadeh, Saleem Rana, Chhabi Lal Ranabhat, Sowmya J Rao, Vahid Rashedi, Prateek Rastogi, Priya Rathi, Salman Rawaf, David Rawaf, Lal Rawal, Reza Rawassizadeh, Giuseppe Remuzzi, Vishnu Renjith, Andre Renzaho, Bhageerathy Reshmi, Serge Resnikoff, Nima Rezaei, Shahab Rezaeian, Aziz Rezapour, Seyed Mohammad Riahi, Ana Isabel Ribeiro, Jennifer Rickard, Alina Rodriguez, Leonardo Roever, Elias Roro, Gholamreza Roshandel, Ali Rostami, Sadegh Rostamnia, Siamak Sabour, Ehsan Sadeghi, Yahya Safari, Rajesh Sagar, Amirhossein Sahebkar, Biniyam Sahiledengle, Mohammad Ali Sahraian, S Mohammad Sajadi, Marwa Salem, Hosni Salem, Marwa Salem, Yahya Salimi, Hamideh Salimzadeh, Evanson Z Sambala, Abdallah M Samy, Itamar Santos, Benn Sartorius, Arash Sarveazad, Brijesh Sathian, Maheswar Satpathy, Sonia Saxena, Mehdi Sayyah, Alyssa Sbarra, Maria Inês Schmidt, Aletta E Schutte, David C Schwebel, Anbissa Muleta Senbeta, Subramanian Senthilkumaran, Seyedmojtaba Seyedmousavi, Faramarz Shaahmadi, Omid Shafaat, Saeed Shahabi, Masood Ali Shaikh, Ali Shalash, Jebreil Shamseddin, Amir Shamshirian, Morteza Shamsizadeh, Mohd Shannawaz, Kiomars Sharafi, Mehdi Sharif, Hatem Shehata, Abbas Sheikhtaheri, Keni Shibuya, Wondimeneh Shiferaw, Mika Shigematsu, Jae Il Shin, Reza Shirkoohi, Ivy Shiue, Shivakumar KM Shivakumar KM, Kerem Shuval, Soraya Siabani, Tariq Jamal Siddiqi, Diego Augusto Santos Silva, Biagio Simonetti, Jasvinder Singh, Virendra Singh, Ambrish Singh, Dhirendra Narain Sinha, Yitagesu Sintayehu, Malede Mequanent Sisay, Amin Soheili, Bijan Soleymani, Farzaneh Soltani, Shahin Soltani, JB Soriano, Muluken Sorrie, Sergey Soshnikov, Ireneous Soyiri, Adel Spotin, Chandrashekhar T Sreeramareddy, Antonina Starodubova, Antonina Starodubova, Agus Sudaryanto, Muawiyyah Babale Sufiyan, Hafiz Ansar Rasul Suleria, Rizwan Suliankatchi Abdulkader, Bruno Sunguya, Bryan L Sykes, Takahiro Tabuchi, Birkneh Tilahun Tadesse, Zemenu Tadesse, Koku Tamirat, Segen Tassew, Cuong Tat Nguyen, Nuno Taveira, Girmay Teklay, Michael Temene, Mohamad-Hani Temsah, Abdullah Terkawi, Nihal Thomas, Kenean Getaneh Tlaye, Hamid Reza Tohidinik, Marcello Tonelli, Marcos Roberto Tovani-Palone, Bach Tran, Khanh Bao Tran, Manjari Tripathi, Reta Tsegaye, Riaz Uddin, Irfan Ullah, Bhaskaran Unnikrishnan, Era Upadhyay, Muhammad Shariq Usman, Olalekan Uthman, Marco Vacante, Masoud Vaezghasemi, Pascual Valdez, Santosh Varughese, Tommi Vasankari, Yasser Vasseghian, Narayanaswamy Venketasubramanian, Francesco Violante, Vasiliy Vlassov, Sebastian Vollmer, Yasir Waheed, Yuan-Pang Wang, Yafeng Wang, Haidong Wang, Mehran Shams-Beyranvand, Taweewat Wiangkham, Tissa Wijeratne, Haileab Fekadu Wolde, Dawit Zewdu Wondafrash, Adam Wondmieneh, Ai-Min Wu, Gelin Xu, Abbas Yadegar, Ali Yadollahpour, Yuichiro Yano, Sanni Yaya, Vahid Yazdi-Feyzabadi, Alex Yeshaneh, Yigizie Yeshaw, Mekdes Yilma, Mekdes Yilma, Nigus Bililign Yimer, Paul Yip, Naohiro Yonemoto, Seok-Jun Yoon, Yoosik Youm, Mustafa Younis, Zabihollah Yousefi, Chuanhua Yu, Hasan Yusefzadeh, Telma Zahirian Moghadam, Leila Zaki, Sojib Bin Zaman, Maryam Zamanian, Hamed Zandian, Alireza Zangeneh, Nejimu Zepro, Taddese Zerfu, Taye Abuhay Zewale, Yunquan Zhang, Zhi-Jiang Zhang, Arash Ziapour, Sanjay Zodpey, Kamiar Zomorodian, Francis Zotor

Drafting the manuscript or revising it critically for important intellectual content

Jennifer Ross, Sandra Munro, Lauren Schaeffer, Mahdieh Abbasalizad-Farhangi, Masoumeh Abbasi, Hedayat Abbastabar, Kedir Abegaz, Lucas Guimarães Abreu, Isaac Adedeji, Victor Adekanmbi, Abiodun Adeoye, Olatunji Adetokunboh, Davoud Adham, Pana Adrian, Mohsen Afarideh, Mohammad Aghaali, Sepideh Ahmadi, Muktar Ahmed, Rushdia Ahmed, Dessalegn Ajema, Tomi Akinyemiju, Blessing Akombi, Samiah Alam, Genet Melak Alamene, Beriwan Ali, Cyrus Alinia, Hesam Alizade, Syed Aljunid, Amir Almasi-Hashiani, Hesham Al-Mekhlafi, Azmeraw T Amare, Adeladza Amegah, Saeed Amini, Nahla Anber, Robert Ancuceanu, Catalina Liliana Andrei, Carl Abelardo Antonio, Jalal Arabloo, Olatunde Aremu, Johan Ärnlöv, Afsaneh Arzani, Mehran Asadi-Aliabadi, Ali Asadi-Pooya, Samaneh Asgari, Maha Atout, Marcel Ausloos, Euripide Avokpaho, Ashish Awasthi, Beatriz Paulina Ayala Quintanilla, Abbas Azadmehr, Ghasem Azarian, Ebrahim Babaee, Alaa Badawi, Shankar Bakkannavar, Senthilkumar Balakrishnan, Maciej Banach, Agegnehu Bante, Till Bärnighausen, Huda Basaleem, Sanjay Basu, Mohsen Bayati, Dereje Bayissa, Neeraj Bedi, Derrick Bennett, Kidanemaryam Berhe, Adam Berman, Krittika Bhattacharyya, Boris Bikbov, Antonio Biondi, Zebenay Workneh Bitew, Tone Bjørge, Antonio Maria Borzì, Oliver Brady, Dejana Braithwaite, Gabrielle Britton, Shyam Sundar Budhathoki, Sharath Burugina Nagaraja, Reinhard Busse, Alessandra C Goulart, Lucero Cahuana-Hurtado, Luis Alberto Camera, Ismael Campos-Nonato, Jorge Cano, Josip Car, Juan J Carrero, Félix Carvalho, João Mauricio Castaldelli-Maia, Franz Castro, Ester Cerin, Pranab Chatterjee, Bal Govind Chauhan, Bal Govind Chauhan, Mohammad Chehrazi, Devasahayam Christopher, Flavia Cicuttini, Paolo Angelo Cortesi, Vera M Costa, Ewerton Cousin, Giovanni Damiani, Amira Hamed Darwish, Ahmad Daryani, Nicole Davis Weaver, Jan-Walter De Neve, Desta Debalkie Atnafu, Asmamaw Demis, Dereje Demissie, Edgar Denova-Gutiérrez, Nebiyu Dereje, Rupak Desai, Samath Dharmaratne, Govinda Dhungana, Mostafa Dianatinasab, Daniel Diaz, Zahra Sadat Dibaji Forooshani, Isaac Oluwafemi Dipeolu, Hoa Do Thi, Paul Narh Doku, Leila Doshmangir, Manisha Dubey, Bruce Duncan, Aziz Eftekhari, Iman El Sayed, Maysaa El Sayed Zaki, Maha El Tantawi, Iffat Elbarazi, Aisha Elsharkawy, Mohammad Hassan Emamian, Shymaa Enany, Tewodros Eshete, Khalil Eskandari, Sharareh Eskandarieh, Saman Esmaeilnejad, Alireza Esteghamati, Arash Etemadi, Andrea Farioli, Andre Faro, Hossein Farzam, Joao Fernandes, Eduarda Fernandes, Irina Filip, Florian Fischer, Carsten Flohr, Nataliya Foigt, Morenike Folayan, Artem Fomenkov, Artem Fomenkov, Masoud Foroutan, Joel Francis, Takeshi Fukumoto, Kidane Tadesse Gebremariam, Gebreamlak Gebremeskel, Gebrekiros Gebremichael, Gebretsadkan Gebretsadik, Getnet Gedefaw, Ayele Geleto, Hailay Gesesew, Fatemeh Ghaffarifar, Mansour Ghafourifard, Ahmad Ghashghaee, Fariba Ghassemi, Tiffany K Gill, Yordanos Gizachew, Sameer Gopalani, Ayman Grada, Michal Grivna, Rafael Guimarães, Yuming Guo, Rajat Das Gupta, Rajeev Gupta, Rahul Gupta, Nima Hafezi-Nejad, Michael Tamene Haile, Arya Haj-Mirzaian, Brian Hall, Demelash Woldeyohannes Handiso, Graeme Hankey, Ahmed Hasaballah, Milad Hasankhani, Amir Hasanzadeh, Amir Hasanzadeh, Maryam Hashemian, Hamid Y Hassen, Rasmus Havmoeller, Corinna Hawkes, Andualem Henok, Claudiu Herteliu, Hagos Hidru, Chi Hoang Linh, Michael Hole, Ramesh Holla, Enayatollah Homaie Rad, Praveen Hoogar, Masako Horino, Naznin Hossain, Mostafa Hosseini, Sorin Hostiuc, Mihaela Hostiuc, Guoqing Hu, Ayesha Humayun, Bing-Fang Hwang, Segun Emmanuel Ibitoye, Abdelrahman Ibrahim Abushouk, Olayinka Ilesanmi, Milena Ilic, Mohammad-Hasan Imani-Nasab, Sheikh Mohammed Shariful Islam, Chidozie Iwu, Neda Izadi, Nader Jafaribalalami, Morteza Jafarinia, Mihajlo Jakovljevic, Ahamarshan Jayaraman

Nagarajan, Achala Jayatilleke, Ensiyeh Jenabi, Ravi Prakash Jha, Vivekanand Jha, John Ji, Jost B Jonas, Nitin Joseph, Jacek Jozwiak, Mikk Jürisson, Ali Kabir, Hossein Samadi Kafil, André Karch, Mohd Anisul Karim, Amir Kasaeian, Habtamu Kasaye, Habtamu Kasaye, Nicholas Kassebaum, Yousef Khader, Morteza Abdullatif Khafaie, Rovshan Khalilov, Md Nuruzzaman Khan, Ejaz Khan, Mohammad Saud Khan, Muhammad Shahzeb Khan, Mona Khater, Mahalaqua Nazli Khatib, Mohammad Khazaei, Mohammad Taghi Khodayari, Ali Kiadaliri, Neda Kianipour, Daniel Kiirithio, Yun Jin Kim, Adnan Kisa, Tufa Kolola, Hamidreza Komaki, Parvaiz Koul, Ai Koyanagi, Moritz Kraemer, Kewal Krishan, Kris Krohn, Nuworza Kugbey, Vivek Kumar, Om Kurmi, Ben Lacey, Huong Lan Nguyen, Justin Lang, Savita Lasrado, Paul Lee, Shanshan Li, Lee-Ling Lim, Chi Linh Hoang, Simin Liu, Joshua Longbottom, Jaifred Christian Lopez, Mohammed Madadin, Mohamed Magdy Abdel Razek, Hassan Magdy Abdel Razek, Phetole Mahasha, Venkatesh Maled, Shokofeh Maleki, Afshin Maleki, Navid Manafi, Ali Manafi, Farzad Manafi, Borhan Mansouri, Dadi Marami, Francisco Rogerlândio Martins-Melo, Benjamin Massenburg, Mohsen Mazidi, Wahengbam Bigyananda Meitei, Feleke Mekonnen, Walter Mendoza, Ritesh G Menezes, Tuomo Meretoja, Tomasz Miazgowski, Ted R Miller, Edward Mills, Seyed Mostafa Mir, Mohammad Miri, Roya Mirzaei, Prasanna Mithra, Babak Moazen, Dara Mohammad, Karzan Mohammad, Shadieh Mohammadi, Abdollah Mohammadian-Hafshejani, Mousa Mohammadnia-Afrouzi, Reza Mohammadpourhodki, Shafiu Mohammed, Yousef Mohammed, Jemal Abdu Mohammed, Farnam Mohebi, Ali Mokdad, Masoud Moradi, Maziar Moradi-Lakeh, Rahmatollah Moradzadeh, Paula Moraga, Shane Morrison, Abbas Mosapour, Jonathan Mosser, Simin Mouodi, Dariush Mozaffarian, Christopher J L Murray, Kamarul Imran Musa, Ghulam Mustafa, Behnam Nabavizadeh, Mehdi Naderi, Mehdi Naderi, Girish Nadkarni, Seyed Sina Naghibi Irvani, Farid Najafi, Jobert Richie Nansseu, Bruno Nascimento, Vinod C Nayak, Ionut Negoi, Ruxandra Irina Negoi, Josephine Ngunjiri, Dabere Nigatu, Yeshambel Nigatu, Zelalem Nigussie, Jean Jacques Noubiap, Christoph Nowak, Onome Oghenetega, In-Hwan Oh, Olanrewaju Oladimeji, Morteza Oladnabi, Andrew T Olagunju, Tinuke O Olagunju, Bolajoko Olusanya, Jacob Olusanya, Mojisola Oluwasanu, Obinna Onwujekwe, Eyal Oren, Alberto Ortiz, Osayomwanbo Osarenotor, Mayowa Owolabi, Mahesh P A, Dr Jagadish Rao Padubidri, Adrian Pana, Songhomitra Panda-Jonas, Hadi Parsian, Mona Pathak, Dr Sanghamitra Pati, Veincent Christian Pepito, Huyen Phuc Do, David Pigott, Majid Pirestani, Maarten Postma, Farshad Pourmalek, Sergio Prada, Liliana Lucia Preotescu, Zahiruddin Syed Quazi, Navid Rabiee, Mohammad Rabiee, Amir Radfar, Fakher Rahim, Vafa Rahimi-Movaghar, Muhammad Aziz Rahman, Ali Rajabpour-Sanati, Fatemeh Rajati, Saleem Rana, Chhabi Lal Ranabhat, Sowmya J Rao, Davide Rasella, Priya Rathi, Lal Rawal, Sarah Ray, Vishnu Renjith, Andre Renzaho, Bhageerathy Reshmi, Nima Rezaei, Mohammad Sadegh Rezai, Seyed Mohammad Riahi, Ana Isabel Ribeiro, Jennifer Rickard, Leonardo Roever, Sadegh Rostamnia, Anas Saad, Yogesh Sabde, Siamak Sabour, Masoumeh Sadeghi, Saeed Safari, Yahya Safari, Rajesh Sagar, Biniyam Sahiledengle, Mohammad Ali Sahraian, Nasir Salam, Farkhonde Salehi, Saleh Salehi Zahabi, Marwa Salem, Hosni Salem, Hamideh Salimzadeh, Abdallah M Samy, Itamar Santos, Abdur Razzaque Sarker, Arash Sarveazad, Maheswar Satpathy, Sonia Saxena, Maria Inês Schmidt, Aletta E Schutte, David C Schwebel, Seyedmojtaba Seyedmousavi, Omid Shafaat, Saeed Shahabi, Ali Shalash, Mehdi Sharif, Rajesh Sharma, Abbas Sheikhtaheri, Reza Shirkoohi, Shivakumar KM Shivakumar KM, Kerem Shuval, Tariq Jamal Siddiqi, Inga Dora Sigfusdottir, Rannveig Sigurvinsdottir, Diego Augusto Santos Silva, Biagio Simonetti, Pankaj Kumar Singh,

Jasvinder Singh, Yitagesu Sintayehu, Amin Soheili, Farzaneh Soltani, Mojtaba Soltani, JB Soriano, Muluken Sorrie, Chandrashekhar T Sreeramareddy, Antonina Starodubova, Muawiyyah Babale Sufiyan, Gerhard Sulo, Bryan L Sykes, Rafael Tabarés-Seisdedos, Takahiro Tabuchi, Segen Tassew, Cuong Tat Nguyen, Nuno Taveira, Gebretsadkan Hintsa Tekulu, Michael Temene, Nihal Thomas, Maria Titova, Maria Titova, Hamid Reza Tohidinik, Marcello Tonelli, Marcos Roberto Tovani-Palone, Bach Tran, Khanh Bao Tran, Riaz Uddin, Irfan Ullah, Bhaskaran Unnikrishnan, Era Upadhyay, Muhammad Shariq Usman, Olalekan Uthman, Marco Vacante, Sahel Valadan Tahbaz, Santosh Varughese, Tommi Vasankari, Yasser Vasseghian, Yousef Veisani, Narayanaswamy Venketasubramanian, Simone Vidale, Vasiliy Vlassov, Rade Vukovic, Yuan-Pang Wang, Mehran Shams-Beyranvand, Haileab Fekadu Wolde, Dawit Zewdu Wondafrash, Ai-Min Wu, Hossein Yahyazadeh, Vahid Yazdi-Feyzabadi, Tomas Yeheyis, Mekdes Yilma, Mekdes Yilma, Naohiro Yonemoto, Hebat-Allah Yousof, Telma Zahirian Moghadam, Sojib Bin Zaman, Maryam Zamanian, Alireza Zangeneh, Zhi-Jiang Zhang, Xiu-Ju Zhao, Mojtaba Ziaee

Management of the overall research enterprise (for example, through membership in the Scientific Council)

Davoud Adham, Mohammad Aghaali, Hesam Alizade, Fatemeh Amiri, Nahla Anber, Govindakarnavar Arunkumar, Babak Asghari, Ghasem Azarian, Atif Amin Baig, Deborah Carvalho Malta, Giovanni Damiani, Desta Debalkie Atnafu, Aziz Eftekhari, Arash Etemadi, Fatemeh Ezzatifar, Artem Fomenkov, Artem Fomenkov, Yordanos Gizachew, Rezvan Goodarzi, Amir Hasanzadeh, Morteza Jafarinia, Fyezah Jehan, Maryam Khayamzadeh, Kris Krohn, Van Lansingh, Borhan Mansouri, Mohsen Mazidi, George Mensah, Maryam Mirzaei, Shadieh Mohammadi, Ali Mokdad, Masoud Moradi, Girish Nadkarni, Mohsen Naghavi, Javad Nazari, Bolajoko Olusanya, Jacob Olusanya, Deepak Kumar Pasupula, George Patton, Ali Rajabpour-Sanati, Saleem Rana, Priya Rathi, Vishnu Renjith, Seyed Mohammad Riahi, Yahya Safari, Rajesh Sagar, Abdur Razzaque Sarker, Benn Sartorius, Mehdi Sayyah, Faramarz Shaahmadi, Keni Shibuya, Pushpendra Singh, Yitagesu Sintayehu, Farzaneh Soltani, Degena Tadesse, Maria Titova, Maria Titova, Era Upadhyay, Yasser Vasseghian
