# Supplementary material for: Mapping local patterns of childhood overweight and wasting in low- and middle-income countries between 2000 and 2017
Source: Nat Med. 2020 Apr 20;26(5):750–9. doi: 10.1038/s41591-020-0807-6 (PMC7220891; doi:10.1038/s41591-020-0807-6)
Supplement: Supplementary file 2 — Reporting Summary [file 41591_2020_807_MOESM2_ESM.pdf]

nature research

Corresponding author(s): Simon I. Hay

Last updated by author(s): 01/10/2020

Reporting Summary

Nature Research wishes to improve the reproducibility of the work that we publish. This form provides structure for consistency and transparency in reporting. For further information on Nature Research policies, see Authors & Referees and the Editorial Policy Checklist.

Statistics

For all statistical analyses, confirm that the following items are present in the figure legend, table legend, main text, or Methods section.

n/a	Confirmed	
☐	☒	The exact sample size (*n*) for each experimental group/condition, given as a discrete number and unit of measurement
☐	☒	A statement on whether measurements were taken from distinct samples or whether the same sample was measured repeatedly
☒	☐	The statistical test(s) used AND whether they are one- or two-sided<br>*Only common tests should be described solely by name; describe more complex techniques in the Methods section.*
☐	☒	A description of all covariates tested
☐	☒	A description of any assumptions or corrections, such as tests of normality and adjustment for multiple comparisons
☐	☒	A full description of the statistical parameters including central tendency (e.g. means) or other basic estimates (e.g. regression coefficient) AND variation (e.g. standard deviation) or associated estimates of uncertainty (e.g. confidence intervals)
☒	☐	For null hypothesis testing, the test statistic (e.g. *F*, *t*, *r*) with confidence intervals, effect sizes, degrees of freedom and *P* value noted<br>*Give P values as exact values whenever suitable.*
☐	☒	For Bayesian analysis, information on the choice of priors and Markov chain Monte Carlo settings
☐	☒	For hierarchical and complex designs, identification of the appropriate level for tests and full reporting of outcomes
☒	☐	Estimates of effect sizes (e.g. Cohen's *d*, Pearson's *r*), indicating how they were calculated

Our web collection on statistics for biologists contains articles on many of the points above.

Software and code

Policy information about availability of computer code

Data collection	No primary data collection was carried out for this analysis
Data analysis	This analysis was carried out using R version 3.5.0. The main geostatistical models were fit using R-INLA version 18.07.12. Additional adjustments were performed using the mgcv package in R (v. 3.5.0). All code used for these analyses is publicly available online at http://ghdx.healthdata.org/. Maps were generated using ArcGIS Desktop 10.6.

For manuscripts utilizing custom algorithms or software that are central to the research but not yet described in published literature, software must be made available to editors/reviewers. We strongly encourage code deposition in a community repository (e.g. GitHub). See the Nature Research guidelines for submitting code & software for further information.

Data

Policy information about availability of data

All manuscripts must include a data availability statement. This statement should provide the following information, where applicable:

- Accession codes, unique identifiers, or web links for publicly available datasets
- A list of figures that have associated raw data
- A description of any restrictions on data availability

The findings of this study are supported by data available in public online repositories, data that are publicly available upon request from the data provider, and data that are not publicly available due to restrictions by the data provider and which were used under license for the current study. A detailed table of data sources and availability can be found in Supplementary Table 2, and online at ghdx.healthdata.org.

Field-specific reporting

Please select the one below that is the best fit for your research. If you are not sure, read the appropriate sections before making your selection.

☒ Life sciences ☐ Behavioural & social sciences ☐ Ecological, evolutionary & environmental sciences

For a reference copy of the document with all sections, see nature.com/documents/nr-reporting-summary-flat.pdf

Life sciences study design

All studies must disclose on these points even when the disclosure is negative.

Sample size	Sample size was calculated as the number of unique data source-location pairs with observations of overweight and wasting prevalence. This sample size is reported in the main text under Global and location variation in malnutrition trends,"...using data from 420 household surveys representing more than 3 million children, we map the relative burdens of overweight and wasting among under-5 children in 105 LMICs from 2000 to 2017."
Data exclusions	Reasons for data exclusion were pre-established and are described in supplementary table 5. For a survey to be considered for this analysis, we required information on height, weight, age and sex. Select data sources were excluded from the analysis due to: missing survey weights, missing sex and age variable, incomplete sampling (e.g., only a specific age range), or untrustworthy data (as determined by the survey administrator or by inspection).
Replication	This is an observational study using many years of survey and surveillance data and could be replicated.
Randomization	This analysis is an observational mapping study and there were no experimental groups.
Blinding	Blinding was not relevant to this study, as it was an observational study using survey and surveillance data.

Reporting for specific materials, systems and methods

We require information from authors about some types of materials, experimental systems and methods used in many studies. Here, indicate whether each material, system or method listed is relevant to your study. If you are not sure if a list item applies to your research, read the appropriate section before selecting a response.

Materials & experimental systems

n/a	Involved in the study	
☒	☐	Antibodies
☒	☐	Eukaryotic cell lines
☒	☐	Palaeontology
☒	☐	Animals and other organisms
☒	☐	Human research participants
☒	☐	Clinical data

Methods

n/a	Involved in the study	
☒	☐	ChIP-seq
☒	☐	Flow cytometry
☒	☐	MRI-based neuroimaging
